# Disulfated Ophiuroid Type Steroids from the Far Eastern Starfish *Pteraster marsippus* and Their Cytotoxic Activity on the Models of 2D and 3D Cultures

**DOI:** 10.3390/md20030164

**Published:** 2022-02-24

**Authors:** Alla A. Kicha, Anatoly I. Kalinovsky, Timofey V. Malyarenko, Olesya S. Malyarenko, Svetlana P. Ermakova, Roman S. Popov, Valentin A. Stonik, Natalia V. Ivanchina

**Affiliations:** G.B. Elyakov Pacific Institute of Bioorganic Chemistry, Far Eastern Branch of the Russian Academy of Sciences, Pr. 100-let Vladivostoku 159, 690022 Vladivostok, Russia; kaaniw@piboc.dvo.ru (A.I.K.); malyarenko-tv@mail.ru (T.V.M.); malyarenko.os@gmail.com (O.S.M.); svetlana_ermakova@hotmail.com (S.P.E.); prs_90@mail.ru (R.S.P.); stonik@piboc.dvo.ru (V.A.S.); ivanchina@piboc.dvo.ru (N.V.I.)

**Keywords:** disulfated steroids, NMR spectra, starfish, *Pteraster marsippus*, cytotoxic activity, 3D culture

## Abstract

New steroidal 3β,21-disulfates (**2**–**4**), steroidal 3β,22-disulfate (**5**), and the previously known related steroidal 3β,21-disulfate (**1**) were isolated from the ethanolic extract of the Far Eastern starfish *Pteraster marsippus*, collected off Urup Island in the Sea of Okhotsk. The structures of these compounds were determined by intensive NMR and HRESIMS techniques as well as by chemical transformations. Steroids **2** and **3** have an oxo-group in the tetracyclic nucleus at position C-7 and differ from each other by the presence of the 5(6)-double bond. The Δ^24^-22-sulfoxycholestane side chain of the steroid **5** has not been found previously in the starfish or ophiuroid steroids. The cytotoxic activities of **1**, **4**, **5**, and the mixture of **2** and **3** were determined on the models of 2D and 3D cultures of human epithelial kidney cells (HEK293), melanoma cells (SK-MEL-28), small intestine carcinoma cells (HuTu80), and breast carcinoma cells (ZR-75-1). The mixture of **2** and **3** revealed a significant inhibitory effect on the cell viability of human breast carcinoma ZR-75-1 cells, but other tested compounds were less effective.

## 1. Introduction

Marine sulfated steroids are often found in representatives of two classes of marine echinoderms, namely ophiuroids and particularly starfish (the phylum Echinodermata), and in sponges (the phylum Porifera) [1,2]. These compounds have been reported to exhibit various biological activities, including anticancer, antimicrobial, cardiovascular, and antifouling properties [3]. Steroidal monosulfates, encountered in different species of starfish, are represented by sterol sulfates and polyhydroxysteroids, containing from four to nine hydroxy groups and a sulfate group at different positions of the tetracyclic core and side chains. In that position, the polyhydroxysteroids were found in both free and glycosylated forms with one to three monosaccharide units and also were found in sulfated form. Moreover, the most common steroidal oligoglycosides of starfish are known as classical asterosaponins and contain an oligosaccharide chain, attached to C-6 and including five or six monosaccharide residues and a sulfate group at C-3 [4,5,6,7,8,9,10]. On the other hand, characteristic secondary metabolites of ophiuroids are mainly steroidal disulfates that differ from other sulfated compounds of echinoderms in some structural peculiarities, namely in the presence of sulfoxy groups at 3α and 21 positions in 5β-, or Δ^5^-, and very rarely 5α-cholestane cores. It is of interest, that similar steroidal disulfates, containing sulfoxy groups at 3β (or 3α) and 21 positions in 5α-, or Δ^5^-cholestane nuclei were found in some species of the Pterasteridae family belonging to the Asteroidea class. From six species of starfish belonging to the Pterasteridae family, in particular *Euretaster insignis* [11], *Pteraster* sp. and *Pteraster tessellatus* [12,13], *Diplopteraster multipes* [14], *Pteraster pulvillus* [15], and *Pteraster obscurus* [16], nine new disulfated steroidal compounds and six compounds studied as desulfated derivatives, obtained after solvolytic desulfation have been structurally described. At the same time, the polyhydroxylated steroids and asterosaponins, common in starfish, were absent in the studied starfish species belonging to the family Pterasteridae. Based on the structural similarity of steroidal disulfates isolated from this family of starfish and from different ophiuroids, it was assumed that there is a closer phylogenetic relationship between the Asteroidea and Ophiuroidea classes than other classes of Echinodermata [13].

At the present time, structural studies on steroidal disulfates from starfish and ophiuroids are somewhat ahead of the investigation of their biological activities. Nevertheless, the steroidal metabolites of ophiuroids were reported to inhibit the protein tyrosine kinase (PTK) [17], to show antiviral activity against HIV-1 and HIV-2 [18], and to be potent antagonists of farnesoid-X-receptor (FXR), a ligand-regulated transcription factor involved in the supporting of the lipid and glucose homeostasis in mammals [19]. These compounds enhanced oxygen-dependent metabolism, increased adhesive and phagocytic properties, induced the expression of pro-inflammatory cytokines TNF-α and IL-8 in neutrophils, and enhanced the production of antibody-forming cells in the mouse spleen [20]. Biological activities of steroidal disulfates from Pterasteridae starfish were less studied, but hemolytic activity on mouse erythrocytes was reported [15]. Thereby, the investigation of the biological activities of steroidal disulfates of starfish and ophiuroids requires further continuation.

In the present article, we describe the results of our study on the fraction of sulfated steroids from the ethanolic extract of the Far Eastern starfish *Pteraster marsippus* Fisher, 1910 (order Velatida, family Pterasteridae) collected by trawling at a depth of 84–88 m in the Sea of Okhotsk near Urup Island. We have isolated and structurally elucidated four new disulfated steroids **2**–**5** along with one previously known related compound **1**. Additionally, the cytotoxic activities of **1**, **4**, **5**, and the mixture of **2** and **3** on the models of 2D and 3D cancer cell cultures have been determined.

## 2. Results

### 2.1. The Isolation and Structure Elucidation of Compounds ***1***–***5*** from P. marsippus

**Figure 1:**
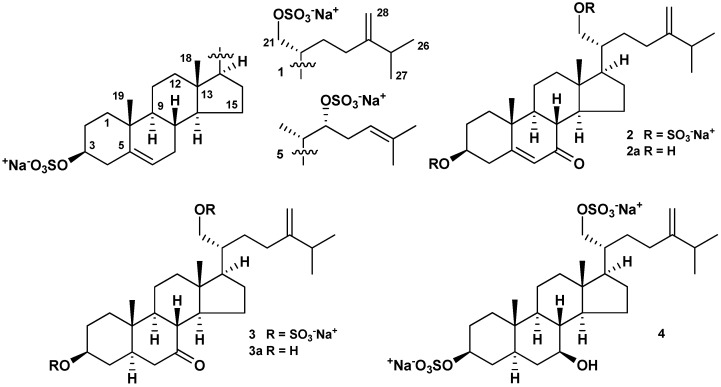
Structures of compounds **1**–**5** isolated from *P. marsippus*.

The ethanol extract of the starfish *P. marsippus* was separated by column chromatography on Polychrome 1, Si gel, and Florisil followed by reversed-phase HPLC on semi-preparative Discovery C18 and analytical YMC-Pack Pro C18 columns to give four new disulfated steroids **2**–**5** along with one previously known related compound **1** (Figure 1).

The molecular formula of steroid **1** was established to be C_28_H_44_Na_2_O_8_S_2_ from the [M − Na]^−^ and [M − 2Na]^2−^ ion peaks at *m*/*z* 595.2388 and 286.1253 in the (−)HRESIMS, respectively, and from the [M + Na]^+^ sodium adduct ion peak at *m*/*z* 641.2154 in the (+)HRESIMS (Figure S1). The presence of sulfate groups in **1** is confirmed by HRESIMS as well as by the presence in the (−)HRESIMS/MS spectrum of [M−2Na]^2−^ ion of fragment ions at *m*/*z* 96.9610 [HSO_4_]^−^, 136.9917 [C_3_H_5_O_4_S]^−^, 391.1958 [M − Na − NaHSO_4_ − C_6_H_12_]^−^, 459.2584 [M − Na − NaHSO_4_ − CH_4_]^−^, and 475.2898 [M − Na − NaHSO_4_]^−^. The ^1^H- and ^13^C-NMR spectroscopic data attributable to the tetracyclic nucleus of **1** revealed the proton and carbon chemical shifts of two angular methyl groups CH_3_-18 (*δ*_H_ 0.75 s, *δ*_C_ 12.5) and CH_3_-19 (*δ*_H_ 1.03 s, *δ*_C_ 19.7), an oxygenated methine CH-3 (*δ*_H_ 4.13 m, *δ*_C_ 79.9), and the 5(6) double bond (*δ*_H_ 5.38 m; *δ*_C_ 141.7, 123.2). The proton and carbon resonances of CH_3_-18, CH_3_-19, CH-3, C-5, CH-6 and the broad multiplet of H-3 (Δ*W* = 39.3 Hz) indicated a Δ^5^-3β-sulfoxy steroidal nucleus in **1** [11].

The proton and carbon signals belonging to the side chain of **1** showed the presence of two secondary methyls CH_3_-26 [*δ*_H_ 1.02 d (*J* = 6.8); *δ*_C_ 22.4] and CH_3_-27 [*δ*_H_ 1.03 d (*J* = 6.8); *δ*_C_ 22.5], an distinctive oxygenated methylene CH_2_-21 [*δ*_H_ 4.21 dd (*J* = 9.8, 3.7), 3.94 dd (*J* = 9.8, 6.4); *δ*_C_ 69.3], and the 24(28) double bond [*δ*_H_ 4.71 br s, 4.68 br d (*J* = 1.3); *δ*_C_ 157.9, 106.9]. These data testified about the Δ^24(28)^-21-sulfoxy-24-methylcholestane side chain in **1** [11,16]. An analysis of the COSY, HSQC, HMBC, and ROESY spectra supported the proposed structure of tetracyclic and side-chain moieties and allowed us to define all the proton and carbon signals in **1** (Table 1 and Table 2, Figures S2–S7). The COSY and HSQC experiments showed a spin coupling system of the protons at C-1 to C-4, C-6 to C-12 through C-11, C-14 to C-17, C-17 to C-21 through C-20, C-20 to C-23, and at C-25 to C-26 and C-27 (Figures S4 and S5). The overall steroid structure of **1** was confirmed by the key HMBC correlations H_3_-18/C-12, C-13, C-14, C-17; H_3_-19/C-1, C-5, C-9, C-10; H_2_-21/C-17, C-20; H-25/C-26, C-27; H_3_-26/C-24; and H_2_-28/C-25, C-26 (Figure S6). The presence of the key ROESY cross-peaks Hα-4/H-6; H-9/Hα-1, Hα-7; Hα-16/H-17; Hβ-16/H-20; H_3_-18/H-8, Hβ-12, Hβ-15, Hβ-16; H_3_-19/Hβ-1, Hβ-2, Hβ-4; H-28/H_2_-22, H_2_-23; and H′-28/H-25, H_3_-26, H_3_-27 exhibited the Δ^5,24(28)^-24-methylcholestane skeleton in **1** (Figure S7). The 20*R* configuration was determined based on the ROESY correlations of Hβ-12/H-20, H_3_-18/H-20, H_2_-21 [21] and the coupling constants and chemical shifts of the methylene group CH_2_-21, which were close to similar values in the ^1^H- and ^13^C-NMR spectra of related previously studied (20*R*)-21-sulfoxysteroids from starfish and ophiuroids [11,13,14,15,16,22]. Thus, the structure (20*R*)-24-methylcholesta-5,24(28)-diene-3β,21-diyl disulfate disodium salt was assigned for **1**. Compound **1** was previously found in the mixture of disulfated 3β,21-dihydroxysteroids from the starfish *Euretaster insignis* [11]. Its structure was proposed on the basis of the structure definition of the desulfated derivative, (20*R*)-24-methylcholesta-5,24(28)-diene-3,21-diol, obtained by solvolysis of the steroid mixture followed by HPLC separation. The ^1^H- and ^13^C-NMR spectroscopic data of **1** itself are presented here for the first time.

An attempt to separate compounds **2** and **3** using repeated reversed-phase HPLC were failed. However, structures of **2** and **3** were established in the mixture by the thorough analysis of the 1D and 2D NMR spectra, including ^1^H- and ^13^C-NMR, 1D TOCSY, COSY, HSQC, HMBC, and ROESY experiments (Figures S8–S13). The molecular formula of steroid **2** was determined to be C_28_H_42_Na_2_O_9_S_2_ from the [M − Na]^−^ and [M − 2Na]^2−^ ion peaks at *m*/*z* 609.2177 and 293.1148 in the (−)HRESIMS, respectively, and from the [M + Na]^+^ sodium adduct ion peak at *m*/*z* 655.1943 in the (+)HRESIMS (Figure S14). The presence of sulfate groups in **2** is confirmed by HRESIMS as well as by the presence in the (−)HRESIMS/MS spectrum of [M−2Na]^2^^−^ of fragment ions at *m*/*z* 96.9612 [HSO_4_]^−^, 136.9917 [C_3_H_5_O_4_S]^−^, 405.1745 [M − Na − NaHSO_4_ − C_6_H_12_]^−^ and 489.2682 [M − Na − NaHSO_4_]^−^. The molecular formula of steroid **3** was established to be C_28_H_44_Na_2_O_9_S_2_ from the [M − Na]^−^ and [M − 2Na]^2^^−^ ion peaks at *m*/*z* 611.2299 and 294.1222 in the (−)HRESIMS, respectively, and from the [M + Na]^+^ sodium adduct ion peak at *m*/*z* 657.2077 in the (+)HRESIMS (Figure S14). The presence of sulfate groups in **3** is confirmed by HRESIMS as well as by the presence in the (−)HRESIMS/MS spectrum of [M − 2Na]^2−^ of fragment ions at *m*/*z* 96.9612 [HSO_4_]^−^, 136.9917 [C_3_H_5_O_4_S]^−^, 407.1891 [M − Na − NaHSO_4_ − C_6_H_12_]^−^, and 491.2825 [M − Na − NaHSO_4_]^−^. The detailed comparison of the ^1^H- and ^13^C-NMR, mass spectra of **1,** and the mixture of **2** and **3** clearly indicated that these compounds have the same Δ^24(28)^-21-sulfoxy-24-methylcholestane side chain, and steroids **2** and **3** differ from **1** by the existence of an additional oxo-group in tetracyclic pattern (Table 1 and Table 2). Moreover, it followed from the chemical shifts and intensities of the proton signals in the ^1^H-NMR spectrum that **2** unlike **3** has a supplementary double bond in the steroidal nucleus, which agreed with the molecular mass difference of 2 amu between **2** and **3** in the mass-spectra.

**Figure 2:**
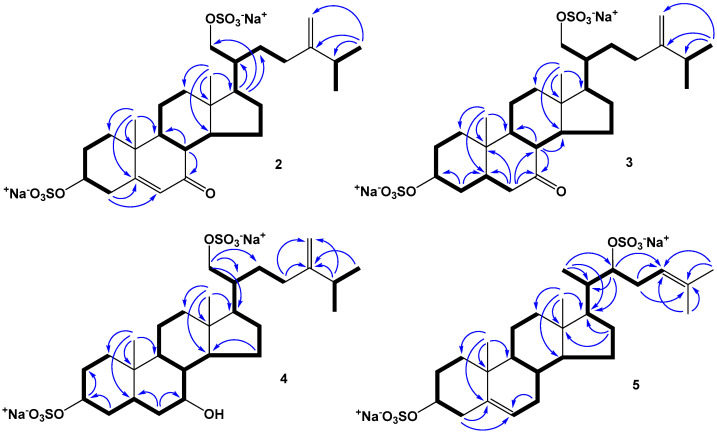
COSY and key HMBC correlations of compounds **2**–**5**.

**Figure 3:**
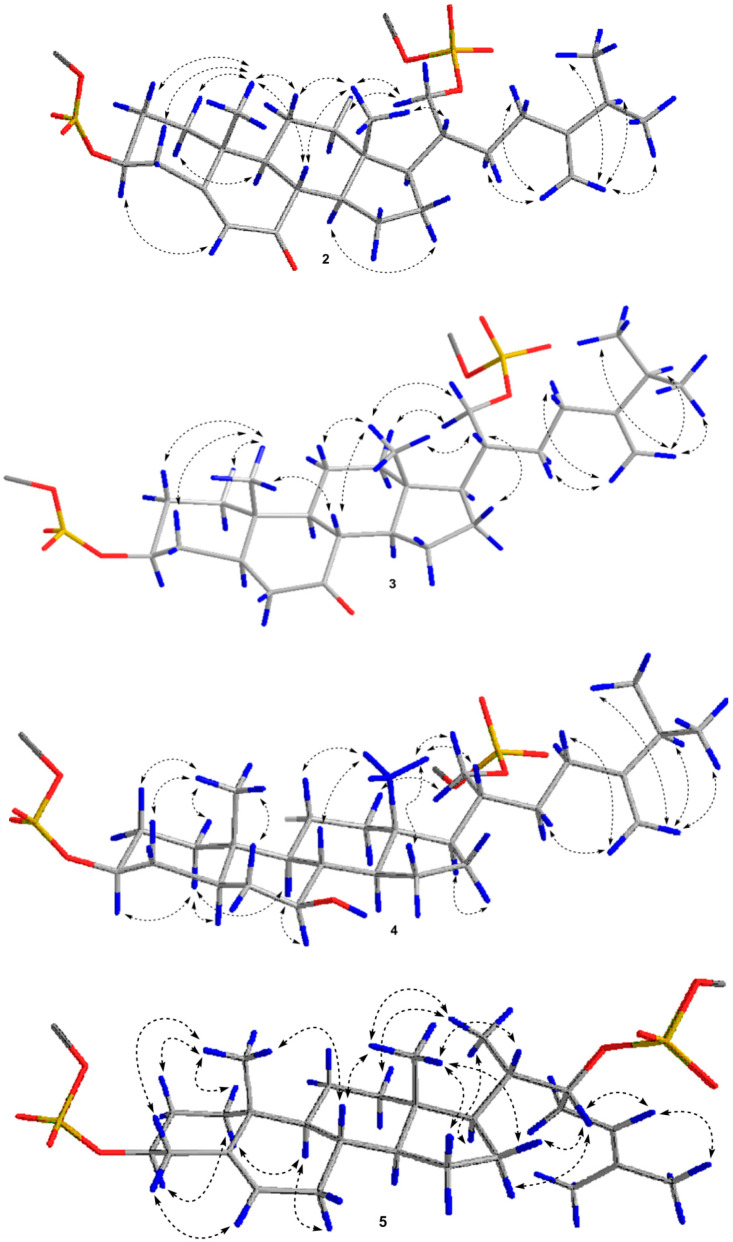
Key ROESY correlations for compounds **2**–**5**. Colors reveal the atoms of hydrogen (blue), oxygen (red), sulfur (yellow), and carbon (grey) and their bonds.

The proton and carbon resonances of two angular methyl groups CH_3_-18 (*δ*_H_ 0.75 s, *δ*_C_ 12.7) and CH_3_-19 (*δ*_H_ 1.24 s, *δ*_C_ 17.6), an oxygenated methine CH-3 (*δ*_H_ 4.26 m, *δ*_C_ 78.0), a 5(6) double bond [*δ*_H_ 5.68 br d (*J* = 1.6); *δ*_C_ 168.3, 126.8], and a 7-oxo group (*δ*_C_ 204.4) attributable to the tetracyclic moiety of **2** were observed in the ^1^H- and ^13^C-NMR spectra. These values of chemical shifts allowed us to suppose a Δ^5^-7-oxo-3β-sulfoxy steroidal nucleus in **2**. The COSY and HSQC experiments led to the identification of the proton sequences at C-1 to C-4, C-8 to C-12 through C-11, C-8 to C-17 through C-14, C-17 to C-21 through C-20, C-20 to C-23, and at C-25 to C-26 and C-27 (Figure 2). Since it is difficult to identify some proton signals in the ^1^H-NMR spectrum of a mixture of two compounds only using 2D NMR experiments, the irradiation of protons Hα-2, Hβ-4, H-6, and H-8 of **2** in the 1D TOCSY experiments was additionally performed, which gave enhancing signals of the neighboring protons H_2_-1, Hβ-2, H-3, and H_2_-4; H_2_-1, H_2_-2, H-3, and Hα-4; H-3 and H_2_-4; and H-9, H-11, H-14, and Hβ-15, respectively. The key HMBC correlations H-4/C-5, C-6; H-8/C-7, C-9; H-17/C-20, C-21, C-22; H_3_-18/C-12, C-13, C-14, C-17; H_3_-19/C-1, C-5, C-9, C-10; and H_3_-26/C-24, C-25, C-28; and the key ROESY correlations Hα-1/H-9; Hα-4/H-6; Hβ-12/H_2_-21; H-14/Hα-16; H_3_-18/H-8, Hβ-11, H-20, H_2_-21; H_3_-19/Hβ-1, Hβ-2, Hβ-4, H-8, Hβ-11; H-28/H_2_-22, H_2_-23; and H′-28/H-25, H_3_-26, H_3_-27 exhibited a 3β,21-disulfoxy-7-oxo pattern in the Δ^5,24(28)^-24-methylcholestane skeleton in **2** (Figure 2 and Figure 3). Based on the above-mentioned data, the structure of **2** was defined as (20*R*)-7-oxo-24-methylcholesta-5,24(28)-diene-3β,21-diyl disulfate disodium salt.

The ^1^H- and ^13^C-NMR spectroscopic data belonging to the steroidal nucleus of **3** displayed the proton and carbon resonances of two angular methyl groups CH_3_-18 (*δ*_H_ 0.72 s, *δ*_C_ 12.6) and CH_3_-19 (*δ*_H_ 1.12 s, *δ*_C_ 12.0), an oxygenated methine CH-3 (*δ*_H_ 4.25 m, *δ*_C_ 78.8), and a 7-oxo group (*δ*_C_ 214.3). These chemical resonances and the absence of a 5(6) double bond corresponded to a 7-oxo-3β-sulfoxy tetracyclic pattern in **3** (Table 1 and Table 2). The COSY and HSQC experiments revealed a spin coupling system of the protons at C-1 to C-6, C-8 to C-12 through C-11, C-8 to C-17 through C-14, C-17 to C-21 through C-20, C-20 to C-23, and at C-25 to C-26 and C-27 (Figure 2). In addition, the irradiation of protons Hβ-1 and Hα-6 of **3** in the 1D TOCSY experiments gave the chemical shifts of neighboring protons: Hα-1, Hα-2, H-3, Hα-4, and H_2_-6; H_2_-1, Hα-2, H-3, Hα-4, and Hβ-6, respectively. In the HMBC spectrum the correlations H-4/C-3, C-5; H-6/C-5, C-7, C-8, C-10; H-8/C-9, C-14; H_3_-18/C-12, C-13, C-14, C-17; H_3_-19/C-1, C-5, C-9, C-10; and H_3_-26/C-24, C-25, C-28, and, in the ROESY spectrum, the cross-peaks Hβ-12/H_2_-21; Hβ-17/H-20; H_3_-18/H-8, Hβ-11, H-20, H_2_-21; H_3_-19/Hβ-1, Hβ-2, Hβ-4, H-8; H-28/H_2_-22, H_2_-23; and H′-28/H-25, H_3_-26, H_3_-27 indicated a 3β,21-disulfoxy-7-oxo substitution in the Δ^24(28)^-24-methyl-5α-cholestane skeleton in **3** (Figure 2 and Figure 3). Thus, the structure of **3** was determined as (20*R*)-7-oxo-24-methyl-5α-cholest-24(28)-ene-3β,21-diyl disulfate disodium salt. Evaluation of the intensities of the CH_3_-18 and CH_3_-19 signals in the ^1^H- and ^13^C-NMR spectra showed a ratio **2** and **3** in the mixture of approximately 1:1 for with a slight advantage of **2**.

Solvolysis of the mixture of **2** and **3** in dioxane/pyridine afforded the mixture of desulfated derivatives **2a** and **3a**, which were separated by HPLC on YMC-Pack-Pro C18 column to give individual compounds. The molecular formula of desulfated steroid **2a** was established to be C_28_H_44_O_3_ from the [M − H]^−^ ion peak at *m*/*z* 427.3215 in the (−)HRESIMS and from the [M + Na]^+^ sodium adduct ion peak at *m*/*z* 451.3175 in the (+)HRESIMS, respectively (Figure S15). Along with mass spectra, the presence of the proton and carbon signals characteristic of two angular methyl groups CH_3_-18 (*δ*_H_ 0.73 s, *δ*_C_ 12.7) and CH_3_-19 (*δ*_H_ 1.23 s, *δ*_C_ 17.8), an oxygenated methine CH-3 (*δ*_H_ 3.54 m, *δ*_C_ 71.2), a 5(6) double bond [*δ*_H_ 5.65 m; *δ*_C_ 169.1, 126.3], a 7-oxo group (*δ*_C_ 204.6), an oxygenated methylene CH_2_-21 [*δ*_H_ 3.69 dd (*J* = 10.7, 4.2), 3.54 dd (*J* = 10.7, 5.5); *δ*_C_ 63.2], and a 24(28) double bond [*δ*_H_ 4.73 br s, 4.69 br d (*J* = 1.4); *δ*_C_ 157.8, 106.9], two secondary methyls CH_3_-26 [*δ*_H_ 1.03 d (*J* = 6.8); *δ*_C_ 22.5] and CH_3_-27 [*δ*_H_ 1.03 d (*J* = 6.8); *δ*_C_ 22.3] in the ^1^H- and ^13^C-NMR spectra revealed the structure of **2a** as (20*R*)-7-oxo-24-methylcholesta-5,24(28)-diene-3β,21-diol. The molecular formula of desulfated steroid **3a** was established to be C_28_H_46_O_3_ from the [M − H]^−^ ion peak at *m*/*z* 429.3376 in the (−)HRESIMS and from the [M + Na]^+^ sodium adduct ion peak at *m*/*z* 453.3333 in the (+)HRESIMS, respectively (Figure S16). The ^1^H- and ^13^C-NMR spectra of **3a** contained signals for two angular methyl groups CH_3_-18 (*δ*_H_ 0.70 s, *δ*_C_ 12.8) and CH_3_-19 (*δ*_H_ 1.11 s, *δ*_C_ 12.1), an oxygenated methine CH-3 (*δ*_H_ 3.52 m, *δ*_C_ 71.3), a 7-oxo group (*δ*_C_ 214.4), an oxygenated methylene CH_2_-21 [*δ*_H_ 3.68 dd (*J* = 10.9, 3.8), 3.53 dd (*J* = 10.9, 5.6); *δ*_C_ 63.2], and a 24(28) double bond [*δ*_H_ 4.72 br s, 4.68 br d (*J* = 1.4); *δ*_C_ 157.5, 106.9], two secondary methyls CH_3_-26 [*δ*_H_ 1.03 d (*J* = 6.7); *δ*_C_ 22.5] and CH_3_-27 [*δ*_H_ 1.03 d (*J* = 6.7); *δ*_C_ 22.3] that matched structure **3a** as (20*R*)-7-oxo-24-methyl-5α-cholest-24(28)-ene-3β,21-diol. All the proton and carbon signals belonging to **2a** and **3a** were derived from COSY, HSQC, HMBC, and ROESY experiments (Table 3, Figures S17–S29). The isolation of individual desulfated derivatives **2a** and **3a** additionally confirmed the structures of steroids **2** and **3**.

The molecular formula of steroid **4** was established to be C_28_H_46_Na_2_O_9_S_2_ from the [M − Na]^−^ and [M − 2Na]^2^^−^ ion peaks at *m*/*z* 613.2483 and *m*/*z* 295.1304 in the (−)HRESIMS, respectively, and from the [M + Na]^+^ sodium adduct ion peak at *m*/*z* 659.2243 in the (+)HRESIMS (Figure S30). The presence of sulfate groups in **4** is confirmed by HRESIMS as well as by the presence in the (−)HRESIMS/MS spectrum of [M − 2Na]^2−^ of fragment ions at *m*/*z* 96.9604 [HSO_4_]^−^, 136.9909 [C_3_H_5_O_4_S]^−^, 191.0380 [C_7_H_11_O_4_S]^−^, 409.2047 [M − Na − NaHSO_4_ − C_6_H_12_], and 493.2987 [M − Na − NaHSO_4_]^−^. The detailed comparison of the ^1^H- and ^13^C-NMR spectroscopic data of compounds **4** and **3** revealed that the proton and carbon resonances belonging to the steroidal A, C, and D rings and side chains of **4** are close to those of **3**, indicating the 3β-hydroxy substitution in tetracyclic nucleus and Δ^24(28)^-21-sulfoxy-24-methyl-cholestane side chain in **4**, while the proton and carbon signals of the steroid B ring of **4** substantially differed from those of **3** (Table 1 and Table 2, Figures S31 and S32). The absence of a carbon signal of the oxo group in the ^13^C-NMR spectrum of **4** in comparison with the ^13^C-NMR spectrum of **3** and the appearance of a triplet of doublets at *δ*_H_ 3.25 (*J* = 10.6, 5.2) in the ^1^H-NMR spectrum of **4** in comparison with the ^1^H-NMR spectrum of **3** indicated the presence of a hydroxyl function in the ring B. The attachment of the hydroxyl group at C-7 was deduced from proton and carbon correlations in the COSY, HSQC, and HMBC spectra (Figure 2 and Figures S33–S35). The key ROESY cross-peaks Hα-1/H-3, H-5, H-9; H-7/H-9; Hβ-12/H_2_-21; Hα-16/H-17; H_3_-18/H-8, Hβ-11, Hβ-15, H-20, H_2_-21; and H_3_-19/Hβ-1, Hβ-2, Hβ-4, Hβ-6; broad signal of H-3 and coupling constant *J* = 10.6 Hz of the triplet of doublets of axial proton H-7 confirmed the 3β,7β relative configurations of the oxygenated carbons in the Δ^24(28)^-24-methyl-5α-cholestane skeleton in **4** (Figure 3 and Figure S36). As a result, the structure of **4** was established as (20*R*)-24-methyl-7β-hydroxy-5α-cholest-24(28)-ene-3β,21-diyl disulfate disodium salt.

The molecular formula of steroid **5** was established to be C_27_H_42_Na_2_O_8_S_2_ from the [M − Na]^−^ and [M − 2Na]^2−^ ion peaks at *m*/*z* 581.2216 and *m*/*z* 279.1171 in the (−)HRESIMS, respectively, and from the [M + Na]^+^ sodium adduct ion peak at *m*/*z* 627.1993 in the (+)HRESIMS (Figure S37). The presence of sulfate groups in **5** is confirmed by HRESIMS as well as by the presence in the (−)HRESIMS/MS spectrum of [M − 2Na]^2^^−^ of fragment ions at *m*/*z* 96.9601 [HSO_4_]^−^, 409.2041 [M − Na − NaHSO_4_ − C_5_H_8_]^−^, and 461.2722 [M − Na − NaHSO_4_]^−^. The examination of the ^1^H-, ^13^C-, and 2D NMR spectra of **5** and **1** revealed that both compounds have the identical Δ^5(6)^-3β-sulfoxy tetracyclic moiety, but the proton and carbon resonances of the steroid side chain of **5** differed from those of **1** (Table 1 and Table 2, Figures S38–S43). The proton and carbon signals in the ^1^H- and ^13^C-NMR spectra belonging to the side chain of **1** showed the presence of three methyls CH_3_-21 [*δ*_H_ 0.96 d (*J* = 6.7); *δ*_C_ 12.8], CH_3_-26 (*δ*_H_ 1.69 s; *δ*_C_ 26.0), and CH_3_-27 (*δ*_H_ 1.65 s; *δ*_C_ 18.1), an oxygenated methine CH-22 [*δ*_H_ 4.36 dd (*J* = 10.6, 4.5); *δ*_C_ 82.6], and the 24-double bond [*δ*_H_ 5.05 t (*J* = 7.7); *δ*_C_ 121.2, 134.8]. The proton connectivities from C-21 through C-20 to C-24 in the side chain were ascertained using the COSY and HSQC experiments. The attachment of the sulfoxy group at C-22 and a 24-double bond were supported from the HMBC cross-peaks H_3_-21/C-17, C-20, C-22; H-22/C-17, C-20, C-24; H-23/C-24, C-25; H_3_-26/C-24, C-25; and H_3_-27/C-24, C-25 (Figure 2). The 20*S* configuration was elucidated by the ROESY correlations of Hβ-12/H-20, H-17/H_3_-21, and H_3_-18/H-20, H_3_-21, and the downfield chemical shift of H_3_-21 at *δ*_H_ 0.96 [21,23,24,25]. Based on the 20*S* configuration, we suggested the 22*R* configuration because the ROESY correlations of H-22/H_2_-16 were observed (Figure 3). Similar correlations were observed in the NOEs spectrum of a natural steroid with a (20*S*,22*R*)-22-hydroxycholestane side chain [23]. Accordingly, the structure of **5** was elucidated as (20*S*)-cholesta-5,24-diene-3β,22-diyl disulfate disodium salt. The Δ^24^-22-sulfoxycholestane side chain of the compound **5** has not been known earlier in other starfish or ophiuroid steroids. It’s interesting that the desulfated derivative (20*S*,22*R*)-cholesta-5,24-diene-3β,22-diol or 22*R*-hydroxydesmosterol, related to compound **5**, is a derivative of desmosterol, a biosynthetic precursor of cholesterol. 22*R*-Hydroxydesmosterol was earlier obtained by stereospecific synthesis and shown to have a cytotoxic effect on tumor and hepatoma cells [26,27].

Previously reported feeding experiments labeled with deuterium precursors have shown that polyhydroxysteroids and related steroidal glycosides of starfish are biosynthesized from dietary cholesterol and cholesterol sulfate [28]. Obviously, the precursors of the biosynthesis of steroidal disulfates **1**–**5** are presumably cholesterol or cholesterol sulfate. The biosynthesis of these compounds takes place with the participation of enzymatic systems such as oxygenases, NAD and NADP-dependent dehydrogenases, SAM-methyltransferase, etc. The following hypothetical pathways for the biosynthesis of compounds **2**–**4** are proposed. Compound **1** undergoes changes only in the steroidal side chain in comparison with cholesterol sulfate by oxidation at CH_3_-21 followed by sulfation and introduction of a methylene group by SAM-methyltransferase at C-24 with loss of a proton. The introduction of a hydroxyl group at C-7 of ring B of the steroidal nucleus of **1** gives an intermediate. Oxidation of the hydroxyl group at C-7 in the intermediate leads to the formation of steroid disulfate **2**, and reduction of the 5(6)-double bond leads to the formation of **4**. The end product, obviously, is the steroid disulfate **3**, which can be obtained from both compounds **2** and **4** (by oxidation or reduction). In compound **5**, as well as in **1**, there are no changes in the steroid nucleus, and only the side chain is modified by oxidation with the following sulfation at the C-22 position.

### 2.2. In Vitro Anticancer Activity of Compounds **1**–**5**

Currently, the main cellular model of cell biology is a two-dimensional (2D) monolayer. However, the cell growth in a monolayer does not reflect the true picture of tumor growth in a living organism by many parameters, where interactions not only between the cells of the tumor but also with the surrounding extracellular matrix are of great importance in its progression. The three-dimensional (3D cell culture) model is represented by spheroids, and proved to be the most effective system that is as close as possible in properties and organization to a natural tumor, which is used for screening the potential anticancer drugs [29]. So, the cytotoxic activity of **1**, **4**, and **5** and the mixture of **2** and **3** was determined on the models of 2D and 3D cultures of human epithelial kidney cells (HEK293), melanoma cells (SK-MEL-28), small intestine carcinoma cells (HuTu80), and breast carcinoma cells (ZR-75-1) using the MTS method.

**Figure 4:**
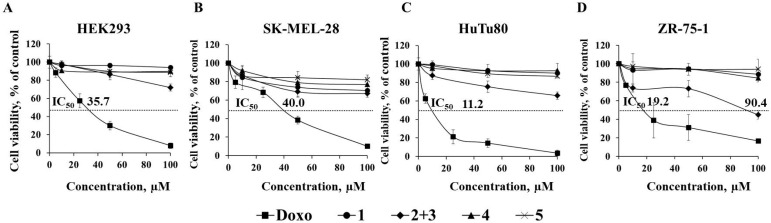
The cytotoxic effect of compounds **1**–**5** on the model of 2D (**A**) HEK293, (**B**) SK-MEL-28, (**C**) HuTu80, and (**D**) ZR-75-1 cells. Cells monolayer was treated with Doxo at concentrations of 5, 25, 50, and 100 μM or **1**, **4**, and **5** and the mixture of **2** and **3** at concentrations of 10, 50, and 100 μM and incubated for 24 h. Cell viability was assessed using the MTS test. Data are presented as means ± standard deviation, as determined in three experiments.

The investigated compounds **1**–**5** were determined to possess moderate cytotoxic activity against normal and cancer cells with the greater impact of the mixture of **2** and **3**. It was found that this mixture inhibited the cell viability of 2D HEK293, SK-MEL-28, HuTu80, and ZR-75-1 by 28, 33, 34, and 55%, respectively, at a concentration of 100 µM after 24 h of treatment (Figure 4A–D). The concentration of the mixture of **2** and **3**, which caused inhibition of 50% cell viability (IC_50_) was established against more sensitive breast carcinoma cells ZR-75-1 as 90.4 µM (Figure 4D). The IC_50_ of doxorubicin (Doxo), used as a positive control, was 35.7, 40.0, 11.2, and 19.2 µM against 2D HEK293, SK-MEL-28, HuTu80, and ZR-75-1, respectively (Figure 4A–D).

**Figure 5:**
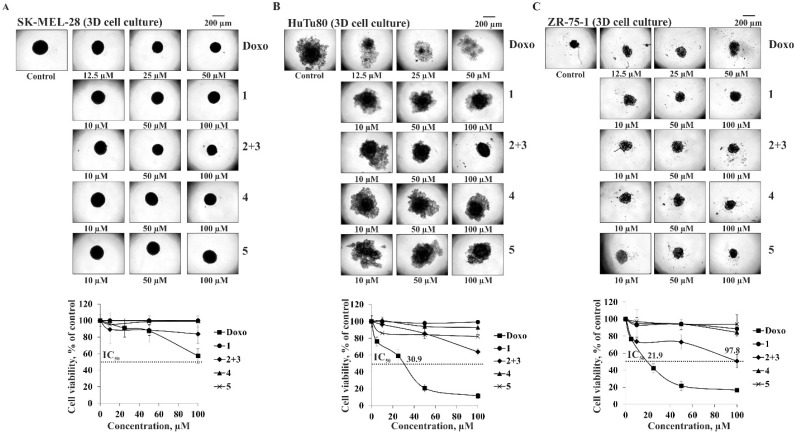
The cytotoxic effect of compounds **1**–**5** on the model of 3D (**A**) SK-MEL-28, (**B**) HuTu80, and (**C**) ZR-75-1 cells. Spheroids were treated with Doxo at concentrations of 5, 25, 50, and 100 μM or **1**, **4**, and **5** and the mixture of **2** and **3** at concentrations of 10, 50, and 100 μM and incubated for 24 h. Cell viability was assessed using the MTS test. Data are presented as means ± standard deviation, as determined in three experiments. Photographs (n = 6 for controls or cells treated with polysaccharides or derivatives, where n = number of photographs) of each spheroid were taken with the ZOE ™ Fluorescent Cell Imager. Spheroids were analyzed using ImageJ software.

The investigated compounds insignificantly affect the size of the spheroids but inhibit their viability to varying degrees (Figure 5A–C). It was determined that the mixture of **2** and **3** inhibited viability of SK-MEL-28, HuTu80, and ZR-75-1 spheroids by 16, 36, and 51%, respectively, at 100 µM after 24 h of treatment. As in the case of 2D culture cells, ZR-75-1 spheroids were the most sensitive to the cytotoxic action of the mixture of **2** and **3**. IC_50_ of Doxo was 30.9 µM and 21.9 µM against HuTu80 and ZR-75-1, respectively.

It should be noted that 3D cell cultures were more resistant to the action of compounds than 2D cultures, which can be explained by dynamic cellular interactions between neighboring cells in spheroids. Moreover, the increased resistance of 3D spheroids may be associated with limited diffusion of the tested substances into the spheroid and hypoxia of cells within the spheroid, which leads to the activation of genes involved in cell survival and the formation of drug resistance [30].

In summary, the results of the present study described the significant inhibiting effect of the mixture of compounds **2** and **3** on the cell viability of human breast carcinoma cells ZR-75-1 in 2D and 3D cell culture models and may contribute to the development of effective chemotherapeutic methods for cancer treatment.

## 3. Materials and Methods

### 3.1. General Procedures

Optical rotations were determined on a Perkin-Elmer 343 polarimeter (PerkinElmer, Waltham, MA, USA). The ^1^H- and ^13^C-NMR spectra were recorded on a Bruker Avance III 700 spectrometer (Bruker BioSpin, Bremen, Germany) at 700.13 and 176.04 MHz, respectively. Chemical shifts (ppm) were internally referenced to the corresponding residual solvent signals at *δ*_H_ 3.30/*δ*_C_ 49.0 for CD_3_OD. HRESIMS mass spectra were recorded on a Bruker Impact II Q-TOF mass spectrometer (Bruker, Bremen, Germany); the samples were dissolved in MeOH (c 0.001 mg/mL). HPLC separations were carried out on an Agilent 1100 Series chromatograph (Agilent Technologies, Santa Clara, CA, USA), equipped with a differential refractometer; Discovery C18 (5 µm, 250 × 10 mm, Supelco, Bellefonte, PA, USA) and YMC-Pack Pro C18 (5 µm, 250 × 4.6 mm, YMC CO., LTD., Kyoto, Japan) columns were used. Low-pressure column liquid chromatography was performed using Polychrom 1 (powdered Teflon, 0.25–0.50 mm; Biolar, Olaine, Latvia) and silica gel KSK (50–160 µm, Sorbpolimer, Krasnodar, Russia). Sorbfil silica gel plates (4.5 × 6.0 cm, 5–17 μm, Sorbpolimer, Krasnodar, Russia) were used for thin-layer chromatography.

### 3.2. Animal Material

Specimens of *Pteraster marsippus* Fisher, 1910 (order Velatida, family Pterasteridae) were collected at a depth of 84–88 m using a small trawl off Urup Island in the Sea of Okhotsk (45.5280 N, 149.4230 E) during the research vessel *Akademik Oparin* 51th scientific cruise in May 2017. Species identification was carried out by Mr. B.B. Grebnev (G.B. Elyakov Pacific Institute of Bioorganic Chemistry of the FEB RAS, Vladivostok, Russia). A voucher specimen [no. 051-039] is on deposit at the marine specimen collection of the G.B. Elyakov Pacific Institute of Bioorganic Chemistry of the FEB RAS, Vladivostok, Russia.

### 3.3. Extraction and Isolation

Freshly collected specimens of *P. marsippus* were frozen and stored at −21 °C until used. The frozen animals (2.1 kg) were cut into small pieces and extracted twice with EtOH at room temperature (2.0 L/kg). The extract was evaporated and the residue (150 g) was dissolved in H_2_O (1.0 L). The H_2_O-soluble fraction was passed through a Polychrom 1 column (8 × 62 cm) and eluted with distilled H_2_O until a negative chloride ion reaction was obtained, followed by elution with 50% aq. EtOH. The combined aq. EtOH eluate was evaporated to give a brownish residue (6.0 g). This fraction was chromatographed over a Si gel column (6.5 × 15 cm) using CHCl_3_/EtOH (stepwise gradient, 4:1 to 1:2, *v*/*v*), EtOH, and EtOH/H_2_O (9:1, *v*/*v*) and rechromatographed over a Florisil column (7 × 15 cm) using CHCl_3_/EtOH (stepwise gradient, 2:1 to 1:1, *v*/*v*) to yield eleven main fractions (1–11) that were analyzed by TLC on Si gel plates in the eluent systems toluene/EtOH (9:5, *v*/*v*) and n-BuOH/EtOH/H_2_O (4:1:2, *v*/*v*/*v*). Fractions 5 and 7 contained the mixtures of disulfated steroids. HPLC separation of fraction 5 (194 mg) on a Discovery C18 column with 54% aq. EtOH (2.0 mL/min) as an eluent system followed by the further separation on the same column with 62% aq. MeOH (2.0 mL/min) as an eluent system yielded pure **1** (1.1 mg, *t*_R_ 18.8 min). HPLC separation of fraction 7 (297 mg) on a Discovery C18 column with 60% aq. MeOH (2.0 mL/min) as an eluent system followed by the further separation on a YMC-Pack Pro C18 column with 60% aq. MeOH (0.6 mL/min) as an eluent system gave the mixture of **2** and **3** (16.1 mg, *t*_R_ 12.8 min), pure **4** (2.3 mg, *t*_R_ 8.5 min) and **5** (1.5 mg, *t*_R_ 16.5 min).

### 3.4. Compound Characterization Data

(20*R*)-24-Methylcholesta-5,24(28)-diene-3β,21-diyl disulfate disodium salt (**1**): Colorless amorphous powder; [α]_D_^25^: −14.5 (*c* 0.11, MeOH); (−)HRESIMS *m*/*z* 595.2388 [M − Na]^−^ (calcd for C_28_H_44_NaO_8_S_2_, 595.2381); (−)HRESIMS *m*/*z* 286.1253 [M − 2Na]^−^ (calcd for C_28_H_44_O_8_S_2_, 286.1244); (+)HRESIMS *m*/*z* 641.2154 [M + Na]^+^ (calcd for C_28_H_44_Na_3_O_8_S_2_, 641.2165); HRESIMS/MS of the [M − 2Na]^2^^−^ ion at *m*/*z* 286.1253: 475.2898 [M − Na − NaHSO_4_]^−^, 459.2584 [M − Na − NaHSO_4_ − CH_4_]^−^, 391.1958 [M − Na − NaHSO_4_ − C_6_H_12_]^−^, 136.9917 [C_3_H_5_O_4_S]^−^, 96.9610 [HSO_4_]^−^; ^1^H-NMR data (see Table 1); ^13^C-NMR data (see Table 2).

(20*R*)-7-Oxo-24-methylcholesta-5,24(28)-diene-3β,21-diyl disulfate disodium salt (**2**); Colorless amorphous powder; the mixture of **2** and **3** [α]_D_^25^: −28.8 (c 0.82, MeOH); (−)HRESIMS *m*/*z* 609.2177 [M − Na]^−^ (calcd for C_28_H_42_NaO_9_S_2_, 609.2173); (−)HRESIMS *m*/*z* 293.1148 [M − 2Na]^2−^ (calcd for C_28_H_42_O_9_S_2_, 293.1141); (+)HRESIMS *m*/*z* 655.1943 [M + Na]^+^ (calcd for C_28_H_42_Na_3_O_9_S_2_, 655.1958); HRESIMS/MS of the [M − 2Na]^2^^−^ ion at *m*/*z* 293.1148: 489.2682 [M − Na − NaHSO_4_]^−^, 405.1745 [M − Na − NaHSO_4_ − C_6_H_12_]^−^, 136.9917 [C_3_H_5_O_4_S]^−^, 96.9612 [HSO_4_]^−^; ^1^H-NMR data (see Table 1); ^13^C-NMR data (see Table 2).

(20*R*)-7-Oxo-24-methyl-5α-cholest-24(28)-ene-3β,21-diyl disulfate disodium salt (**3**); Colorless amorphous powder; the mixture of **2** and **3** [α]_D_^25^: −28.8 (*c* 0.82, MeOH); (−)HRESIMS *m*/*z* 611.2299 [M − Na]^−^ (calcd for C_28_H_44_NaO_9_S_2_, 611.2330); (−)HRESIMS *m*/*z* 294. 1222 [M − 2Na]^2−^ (calcd for C_28_H_44_O_9_S_2_, 294.1219); (+)HRESIMS *m*/*z* 657.2077 [M + Na]^+^ (calcd for C_28_H_44_Na_3_O_9_S_2_, 657.2114); HRESIMS/MS of the [M − 2Na]^2^^−^ ion at *m*/*z* 294.1222: 491.2825 [M − Na − NaHSO_4_]^−^, 407.1891 [M − Na − NaHSO_4_ − C_6_H_12_]^−^, 136.9917 [C_3_H_5_O_4_S]^−^, 96.9612 [HSO_4_]^−^; ^1^H-NMR data (see Table 1); ^13^C-NMR data (see Table 2).

(20*R*)-24-Methyl-7β-hydroxy-5α-cholest-24(28)-ene-3β,21-diyl disulfate disodium salt (**4**); Colorless amorphous powder; [α]_D_^25^: +9.6 (*c* 0.23, MeOH); (−)HRESIMS *m*/*z* 613.2483 [M − Na]^−^ (calcd for C_28_H_46_NaO_9_S_2_, 613.2486); (−)HRESIMS *m*/*z* 295.1304 [M − 2Na]^2−^ (calcd for C_28_H_46_O_9_S_2_, 295.1297); (+)HRESIMS *m*/*z* 659.2243 [M + Na]^+^ (calcd for C_28_H_46_Na_3_O_9_S_2_, 659.2271); HRESIMS/MS of the [M − 2Na]^2^^−^ ion at *m*/*z* 295.1304: 493.2987 [M − Na − NaHSO_4_]^−^, 409.2047 [M − Na − NaHSO_4_ − C_6_H_12_], 191.0380 [C_7_H_11_O_4_S]^−^, 136.9909 [C_3_H_5_O_4_S]^−^, 96.9604 [HSO_4_]^−^; ^1^H-NMR data (see Table 1); ^13^C-NMR data (see Table 2).

(20*S*)-Cholesta-5,24-diene-3β,22-diyl disulfate disodium salt (**5**); Colorless amorphous powder; [α]_D_^25^: −14.0 (c 0.15, MeOH); (−)HRESIMS *m*/*z* 581.2216 [M − Na]^−^ (calcd for C_27_H_42_NaO_8_S_2_, 581.2224); (−)HRESIMS *m*/*z* 279.1171 [M − 2Na]^2−^ (calcd for C_27_H_42_O_8_S_2_, 279.1166); (+)HRESIMS *m*/*z* 627.1993 [M + Na]^+^ (calcd for C_27_H_42_Na_3_O_8_S_2_, 627.2009); HRESIMS/MS of the [M − 2Na]^2^^−^ ion at *m*/*z* 279.1171: 461.2722 [M − Na − NaHSO_4_]^−^, 409.2041 [M − Na − NaHSO_4_ − C_5_H_8_]^−^, 96.9601 [HSO_4_]^−^. ^1^H-NMR data (see Table 1); ^13^C-NMR data (see Table 2).

### 3.5. Solvolysis of the Mixture of ***2*** and ***3***

A solution of the mixture of **2** and **3** (5.0 mg) in 2 mL of dioxane/pyridine (1:1) was heated at 100 °C for 4 h. The reaction mixture was evaporated under reduced pressure and separated by HPLC on a YMC-Pack Pro C18 column with 80% aq. MeOH (0.7 mL/min) as an eluent system to give pure desulfated derivatives **2a** (0.5 mg, *t*_R_ 40.6 min) and **3a** (0.4 mg, *t*_R_ 39.6 min).

(20*R*)-7-Oxo-24-methylcholesta-5,24(28)-diene-3β,21-diol (**2a**); Colorless amorphous powder; [α]_D_^25^: −38.0 (c 0.05, MeOH); (−)HRESIMS *m*/*z* 427.3215 [M − H]^−^ (calcd for C_28_H_43_O_3_, 427.3218); (+)HRESIMS *m*/*z* 451.3175 [M + Na]^+^ (calcd for C_28_H_44_NaO_3_, 451.3183); ^1^H- and ^13^C-NMR data (see Table 3).

(20*R*)-7-Oxo-24-methyl-5α-cholest-24(28)-ene-3β,21-diol (**3a**); Colorless amorphous powder; [α]_D_^25^: −5.0 (c 0.04, MeOH); (−)HRESIMS *m*/*z* 429.3376 [M − H]^−^ (calcd for C_28_H_45_O_3_, 429.3374); (+)HRESIMS *m*/*z* 453.3333 [M + Na]^+^ (calcd for C_28_H_46_NaO_3_, 453.3339); ^1^H- and ^13^C-NMR data (see Table 3). 

### 3.6. Bioactivity Assay

#### 3.6.1. Cell Lines

American Type Culture Collection (Manassas, VA, USA) provided human epithelial kidney cells HEK293 (ATCC^®^ no. CRL-1573™) and melanoma cells SK-MEL-28 (ATCC^®^ no. HTB-72™). Human small intestine carcinoma cells HuTu80 and breast carcinoma cells ZR-75-1 were obtained from the Shared Research Facility’s Vertebrate cell culture collection (Saint-Petersburg, Russia).

#### 3.6.2. Cell Culture Conditions

HEK293 and SK-MEL-28 cells were cultured in Dulbecco’s Modified Eagle Medium (DMEM), HuTu80 cells were maintained in Minimum Essential Medium (MEM), and ZR-75-1 cells were cultured in Roswell Park Memorial Institute Medium (RPMI-1640) in a humidified 5% CO_2_ incubator. The culture medium was supplemented with 10% of fetal bovine albumin (FBS), 100 mg/mL streptomycin, and 100 U/mL penicillin. At 90% confluence, cells were rinsed with PBS, detached from the tissue culture flask by 0.25% trypsin/0.5 mM EDTA, and 10–20% of the harvested cells were transferred to a new flask containing fresh complete appropriate medium. The passage number was carefully controlled and the mycoplasma contamination was monitored on a regular basis.

#### 3.6.3. Preparation of Compounds for the Determination of Cytotoxic Activity

Compounds **1**, **4**, and **5** and the mixture of **2** and **3** were dissolved in sterile dimethyl sulfoxide (DMSO) to prepare stock concentrations of 20 mM. Cells were treated with serially diluted **1**–**5** (10, 50, 100 µM) (culture medium used as diluent) (final concentration of DMSO was less than 0.5%).

Doxorubicin (Doxo) (Teva Pharmaceutical Industries, Ltd., Petah Tikva, Israel) was dissolved in sterile PBS to prepare stock concentrations of 10 mM. Cells were treated with serially diluted Doxo (5, 25, 50, 100 µM) (culture medium used as diluent).

The vehicle control is the cells treated with the equivalent volume of DMSO (final concentration was less than 0.5%) for all of the presented experiments.

#### 3.6.4. Formation of 3D Spheroids by Liquid Overlay Technique (LOT)

SK-ME-28, HuTu80, and ZR-75-1 spheroids were formed by the liquid overlay technique (LOT) method with slight modifications. Briefly, to create non-adherent surfaces for the efficient spheroids’ formation, 50 µL of preheated (60 °C) agarose (1.5%) was overlaid the bottom of 96-well plates and left to solidify for 1 h at room temperature under sterile conditions.

SK-MEL-28 cells (5.0 × 10^3^), HuTu80 (3.0 × 10^3^), and ZR-75-1 (3.0 × 10^3^) were inoculated in an agarose layer and cultured in 200 µL of a complete appropriate culture medium for 96 h at 37 °C in a 5% CO_2_ incubator. An image of each spheroid was made with a ZOE™ Fluorescent Cell Imager (Bio Rad, Hercules, CA, USA). ImageJ software bundled with 64-bit Java 1.8.0_112 (NIH, Bethesda, MD, USA) was used to measure the spheroid integrity, diameter, and volume.

#### 3.6.5. Cytotoxic Activity Assay (MTS)

##### 2D Cell Culture (Monolayer)

HEK293 (0.8 × 10^3^/200 µL), SK-MEL-28 (0.8 × 10^3^/200 µL), HuTu80 (1.0 × 10^3^/200 µL), and ZR-75-1 (1.2 × 10^3^/200 µL) cells were seeded into 96-well plates (Jet Biofil, Guangzhou, China) for 24 h at 37 °C in a 5% CO_2_ incubator. Then cell monolayer was treated either with DMSO (control), Doxo (positive control) (5, 25, 50, 100 µM) or various concentrations of compounds **1**, **4**, and **5** and the mixture of **2** and **3** (10, 50, 100 µM) in fresh appropriate culture medium for 24 h. Subsequently, the cells were incubated with 15 µL of 3-(4,5-dimethylthiazol-2-yl)-5-(3-carboxymethoxyphenyl)-2-(4-sulfophenyl)-2H-tetrazolium (MTS reagent) (Promega, Madison, WI, USA) for 3 h, and the absorbance of each well was measured at 490/630 nm using Power Wave XS microplate reader (BioTek, Wynusky, VT, USA). The concentration at which the compounds exert half of its maximal inhibitory effect on cell viability (IC_50_) was calculated by the AAT-Bioquest^®^ online calculator [31].

##### 3D Cell Culture (Spheroids)

The spheroids were treated by replacing 100 µL of supernatant with a complete medium containing DMSO (control), Doxo (positive control) at 5, 25, 50, 100 µM or compounds **1**, **4**, and **5** and the mixture of **2** and **3** at 10, 50, 100 µM for 24 h. Then, 15 µL of 3-(4,5-dimethylthiazol-2-yl)-5-(3-carboxymethoxyphenyl)-2-(4-sulfophenyl)-2H-tetrazolium (MTS) reagent (Promega, Madison, WI, USA) was added to each well with spheroids and incubated for 3 h at 37 °C in a 5% CO_2_ incubator. The absorbance of each well was measured at 490/630 nm using Power Wave XS microplate reader. A photo of the 3D spheroids (40 × 200 μm scale) was made with the aid of a microscope Motic AE 20 (XiangAn, Xiamen 361101, China) and the ImageJ software.

#### 3.6.6. Statistical Analysis

All of the assays were performed in at least three independent experiments. Results are expressed as the mean ± standard deviation (SD). The Student’s *t*-test was used to evaluate the data with the following significance levels: * *p* < 0.05, ** *p* < 0.01, *** *p* < 0.001.

## 4. Conclusions

Three new 3β,21-disulfated steroids and one new 3β,22-disulfated steroid, along with a previously known related compound, were isolated from the Far Eastern starfish *P. marsippus,* and their chemical structures were established. Two steroids have an oxo-group at position C-7 in steroid nucleus; moreover, one of them additionally includes the conjugated 5,6-double bond. The Δ^24^-22-sulfoxycholestane side chain, indicated in another new steroid, has not been earlier found in starfish and ophiuroid steroidal compounds. Thus, in one more species of starfish, *P. marsippus*, belonging to the Pterasteridae family, like the previously studied six species of starfish of the same family, disulfated steroids of «the ophiuroid type» were found. It should be noted that the polyhydroxylated compounds and asterosaponins common in starfish were absent in the *P. marsippus* as well as in the previously studied species of this family. This fact once again confirms the assumption about a closer phylogenetic relationship between Asteroidea and Ophiuroidea classes compared to other classes of Echinodermata. The mixture of two steroids, having an oxo-group at position C-7 in steroid nucleus, was found to possess the highest cytotoxic activity against 2D and 3D human breast carcinoma cells ZR-75-1 among other investigated by us compounds and can be a candidate for further examination of the molecular mechanism of its anticancer action.

## Figures and Tables

**Table 1:** ^1^H-NMR (700.13 MHz) chemical shifts of compounds **1**–**5** in CD_3_OD, with *δ* in ppm and *J* values in Hz *^a^*.

Position	1	2	3	4	5
1β α	1.89 dt (13.8, 3.7) 1.11 m	2.02 dt (13.9, 3.7) 1.26 td (13.9, 3.5)	1.81 dt (13.9, 3.5) 1.07 td (13.9, 3.7)	1.76 dt (13.8, 3.7) 0.98 td (13.8, 3.8)	1.89 dt (13.5, 3.5) 1.11 m
2α β	2.05 m 1.63 m	2.15 m 1.76 m	2.06 m 1.60 m	2.02 m 1.53 m	2.05 m 1.62 m
3	4.13 m (Δ*W* = 39.3 Hz)	4.26 m	4.25 m	4.24 m	4.13 m
4α β	2.53 ddd (13.2, 4.8, 2.2) 2.34 td (13.2, 2.0)	2.79 ddd (14.0, 5.0, 2.3) 2.54 ddd (14.0, 11.8, 1.9)	1.86 m 1.57 m	1.81 m 1.43 m	2.53 ddd (13.4, 4.8, 2.2) 2.33 m
5	–	–	1.52 m	1.22 m	–
6β α	5.38 m	5.68 br d (1.6)	2.46 t (12.3) 1.94 dd (12.3, 3.2)	1.33 t (12.8) 1.55 m	5.38 m
7β α	1.97 m 1.56 m	–	–	3.25 td (10.6, 5.2)	1.96 m 1.54 m
8	1.48 m	2.31 dd (12.6, 10.7)	2.47 m	1.40 m	1.47 m
9	0.97 td (11.7, 4.6)	1.53 m	1.09 m	0.71 m	0.96 m
10	–	–	–	–	–
11α β	1.57 m 1.04 m	1.63 m	1.62 m 1.55 m	1.58 m 1.35 m	1.54 m 1.05 m
12β α	2.04 m 1.25 td (13.0, 4.2)	2.07 m 1.22 m	2.02 m 1.16 m	1.99 m 1.19 m	2.01 dt (12.8, 3.5) 1.23 td (12.8, 4.2)
13	–	–	–	–	–
14	1.06 m	1.35 m	1.47 m	1.19 m	1.09 m
15α β	1.64 m 1.13 m	2.40 m 1.28 m	2.20 m 1.02 m	1.90 m 1.48 m	1.61 m 1.09 m
16α β	1.87 m 1.37 m	1.90 m 1.39 m	1.88 m 1.35 m	1.83 m 1.34 m	2.22 m 1.16 m
17	1.48 m	1.48 m	1.48 m	1.43 m	1.63 m
18	0.75 s	0.75 s	0.72 s	0.73 s	0.70 s
19	1.03 s	1.24 s	1.12 s	0.86 s	1.02 s
20	1.72 m	1.69 m	1.69 m	1.68 m	1.58 m
21	4.21 dd (9.8, 3.7) 3.94 dd (9.8, 6.4)	4.18 dd (9.6, 4.0) 3.99 dd (9.6, 5.7)	4.17 dd (9.6, 4.1) 3.96 dd (9.6, 5.7)	4.18 dd (9.7, 3.8) 3.94 dd (9.7, 6.2)	0.96 d (6.7)
22	1.64 m 1.48 m	1.64 m 1.49 m	1.64 m 1.49 m	1.64 m 1.48 m	4.36 dd (10.6, 4.5)
23	2.17 ddd (15.0, 11.1, 4.6) 2.04 m	2.19 m 2.02 m	2.19 m 2.02 m	2.17 m 2.03 m	2.66 m 2.34 m
24	–	–	–	–	5.05 t (7.7)
25	2.25 quin (6.7)	2.25 m	2.25 m	2.25 quin	–
26	1.02 d (6.8)	1.02 d (6.8)	1.02 d (6.8)	1.02 d (6.8)	1.69 s
27	1.03 d (6.8)	1.03 d (6.8)	1.03 d (6.8)	1.03 d (6.8)	1.65 s
28	4.71 br s 4.68 br d (1.3)	4.71 br d (1.2) 4.69 br d (1.2)	4.71 br d (1.2) 4.69 br d (1.2)	4.71 br s 4.68 br d (1.5)	

*^a^* Assignments from 700.13 MHz COSY, HSQC, HMBC (8 Hz), and ROESY (250 msec) data; s, singlet; d, doublet; t, triplet; m, multiplet; br s, broad singlet; br d, broad doublet; dd, doublet of doublets; ddd, doublet of doublet of doublets; dt, doublet of triplets; quin, quintet.

**Table 2:** ^13^C-NMR (176.04 MHz) chemical shifts of compounds **1**–**5** in CD_3_OD.

Position	1	2	3	4	5
1	38.5	37.4	37.1	38.1	38.4
2	30.0	29.6	29.4	29.7	30.0
3	79.9	78.0	78.8	79.4	79.9
4	40.4	40.1	36.2	35.9	40.4
5	141.7	168.3	48.2	43.6	141.6
6	123.2	126.8	46.9	39.9	123.3
7	33.0	204.4	214.3	75.7	33.0
8	33.3	46.6	51.1	44.1	33.3
9	51.7	51.4	56.7	54.0	51.6
10	37.7	39.7	37.0	36.0	37.7
11	22.1	22.2	22.9	22.6	22.1
12	40.2	39.1	39.2	40.5	41.0
13	43.4	44.2	43.6	44.5	43.3
14	58.0	51.3	50.4	57.3	58.0
15	25.2	27.3	25.9	27.9	25.4
16	28.7	28.9	28.8	29.0	29.2
17	51.8	50.7	50.7	51.2	53.2
18	12.5	12.7	12.6	12.9	12.1
19	19.7	17.6	12.0	12.7	19.7
20	41.1	41.0	41.0	41.0	39.4
21	69.3	69.2	69.2	69.4	12.8
22	29.7	29.8	29.8	29.7	82.6
23	31.6	31.8	31.8	31.8	31.7
24	157.9	157.8	157.8	157.9	121.2
25	34.9	34.8	34.8	34.9	134.8
26	22.4	22.3	22.4	22.3	26.0
27	22.5	22.5	22.5	22.5	18.1
28	106.9	107.0	107.8	106.9	

**Table 3:** ^1^H-(700.13 MHz) and ^13^C-(176.04 MHz) NMR chemical shifts of compounds **2a** and **3a** in CD_3_OD, with *δ* in ppm and *J* values in Hz *^a^*.

Position	2a		3a	
	*δ*_H_	*δ*_C_	*δ*_H_	*δ*_C_
1β α	1.98 m 1.22 m	37.6	1.77 m 1.03 m	37.3
2α β	1.89 m 1.61 m	31.9	1.80 m 1.46 m	31.8
3	3.54 m	71.2	3.52 m	71.3
4α β	2.48 ddd (13.5, 4.6, 2.1) 2.39 ddd (13.5, 11.5, 2.0)	42.8	1.56 m 1.44 m	48.4
5	–	169.1	1.48 m	169.1
6	5.65 m	126.3	2.45 t (13.0) 1.92 dd (13.0, 3.2)	47.0
7	–	204.6	–	214.4
8	2.31 dd (12.8, 10.8)	46.6	2.47 t (12.1)	51.1
9	1.51 m	51.6	1.08 m	57.0
10	–	39.7	–	37.2
11	1.64 m	22.3	1.62 m 1.56 m	22.9
12β α	1.97 m 1.19 m	39.3	1.92 m 1.14 m	39.4
13	–	44.2	–	43.6
14	1.32 m	51.3	1.40 m	50.4
15α β	2.39 m 1.28 m	27.3	2.19 m 1.01 m	25.9
16α β	1.87 m 1.39 m	28.4	1.86 m 1.37 m	28.4
17	1.45 m	50.6	1.45 m	50.9
18	0.73 s	12.7	0.70 s	12.8
19	1.23 s	17.8	1.11 s	12.1
20	1.51 m	43.2	1.50 m	43.2
21	3.69 dd (10.7, 4.2) 3.54 dd (10.7, 5.5)	63.2	3.68 dd (10.9, 3.8) 3.53 dd (10.9, 5.6)	63.2
22	1.63 m 1.44 m	29.4	1.61 m 1.43 m	29.3
23	2.15 m 1.98 m	32.3	2.13 m 1.97 m	32.3
24	–	157.8	–	157.5
25	2.25 quin	34.9	2.25 quin	34.9
26	1.03 d (6.8)	22.5	1.03 d (6.7)	22.5
27	1.03 d (6.8)	22.3	1.03 d (6.7)	22.3
28	4.73 br s 4.69 br d (1.4)	106.9	4.72 br s 4.68 br d (1.4)	106.9

*^a^* Assignments from 700.13 MHz COSY, HSQC, HMBC (8 Hz), and ROESY (250 msec) data.

## Data Availability

Not applicable.

## Supplementary Materials

The following are available online at https://www.mdpi.com/article/10.3390/md20030164/s1, Copies HRESIMS (Figures S1, S14–S18, S31 and S38), 1H-NMR (Figures S2, S8, S19, S25, S32, and S39), 13C-NMR (Figures S3, S9, S20, S26, S33, and S40), COSY (Figures S4, S10, S21, S27, S34, and S41), HSQC (Figures S5, S11, S22, S28, S20, S35, and S42), HMBC (Figures S6, S12, S23, S29, S36, and S43), and ROESY (Figures S7, S13, S24, S30, S37, and S44) spectra of compounds 1, the mixture of 2 and 3, 2a, 3a, 4, and 5, respectively. COSY, key HMBC, and key ROESY correlations of compounds 2a and 3a (Figure S18).
