# Supplementary material for: Disulfated Ophiuroid Type Steroids from the Far Eastern Starfish Pteraster marsippus and Their Cytotoxic Activity on the Models of 2D and 3D Cultures
Source: Mar Drugs. 2022 Feb 24;20(3):164. doi: 10.3390/md20030164 (PMC8951248; doi:10.3390/md20030164)

Supplementary Materials

Disulfated Ophiuroid Type Steroids from the Far Eastern Starfish *Pteraster marsippus* and their Cytotoxic Activity on the Models of 2D and 3D Cultures

Alla A. Kicha[1]*, Anatoly I. Kalinovsky[1], Timofey V. Malyarenko[1], Olesya S. Malyarenko[1], Svetlana P. Ermakova[1], Roman S. Popov[1], Valentin A. Stonik[1] and Natalia V. Ivanchina[1]

[1] G.B. Elyakov Pacific Institute of Bioorganic Chemistry, Far Eastern Branch of the Russian Academy of Sciences, Pr. 100-let Vladivostoku 159, 690022 Vladivostok, Russia; kaaniw@piboc.dvo.ru (A.I.K.); malyarenko-tv@mail.ru (T.V.M.); malyarenko.os@gmail.com (O.S.M.); svetlana_ermakova@hotmail.com (S.P.E.); prs_90@mail.ru (R.S.P.); stonik@piboc.dvo.ru (V.A.S.); ivanchina@piboc.dvo.ru (N.V.I.)

* Correspondence: kicha@piboc.dvo.ru (A.A.K.); Tel.: +7-423-2312-360; Fax: +7-423-2314-050.

List

Page

Figure S1. HRESIMS spectra of compound **1** 4
Figure S2. ^{1}H NMR (700.13 MHz, CD_3OD) spectrum of compound **1** 5
Figure S3. ^{13}C NMR (176.04 MHz, CD_3OD) spectrum of compound **1** 6
Figure S4. COSY (700.13 MHz, CD_3OD) spectrum of compound **1** 7
Figure S5. HSQC (700.13 MHz, CD_3OD) spectrum of compound **1** 8
Figure S6. HMBC (700.13 MHz, CD_3OD) spectrum of compound **1** 9
Figure S7. ROESY (700.13 MHz, CD_3OD) spectrum of compound **1** 10
Figure S8. ^{1}H NMR (700.13 MHz, CD_3OD) spectrum of the mixture of **2** and **3** 11
Figure S9. ^{13}C NMR (176.04 MHz, CD_3OD) spectrum of the mixture of **2** and **3** 12
Figure S10. COSY (700.13 MHz, CD_3OD) spectrum of the mixture of **2** and **3** 13
Figure S11. HSQC (700.13 MHz, CD_3OD) spectrum of the mixture of **2** and **3** 14
Figure S12. HMBC (700.13 MHz, CD_3OD) spectrum of the mixture of **2** and **3** 15
Figure S13. ROESY (700.13 MHz, CD_3OD) spectrum of the mixture of **2** and **3** 16
Figure S14. HRESIMS spectra of the mixture of **2** and **3** 17
Figure S15. HRESIMS spectra of compound **2a** 18
Figure S16. HRESIMS spectra of compound **3a** 19
Figure S17. (**A**) COSY and key HMBC correlations of the mixture of compounds **2a** and **3a**. (**B**) Key ROESY correlations of the mixture of compounds **2a** and **3a** 20
Figure S18. ^{1}H NMR (700.13 MHz, CD_3OD) spectrum of compound **2a** 21
Figure S19. ^{13}C NMR (176.04 MHz, CD_3OD) spectrum of compound **2a** 22
Figure S20. COSY (700.13 MHz, CD_3OD) spectrum of compound **2a** 23
Figure S21. HSQC (700.13 MHz, CD_3OD) spectrum of compound **2a** 24
Figure S22. HMBC (700.13 MHz, CD_3OD) spectrum of compound **2a** 25
Figure S23. ROESY (700.13 MHz, CD_3OD) spectrum of compound **2a** 26
Figure S24. ^{1}H NMR (700.13 MHz, CD_3OD) spectrum of compound **3a** 27
Figure S25. ^{13}C NMR (176.04 MHz, CD_3OD) spectrum of compound **3a** 28
Figure S26. COSY (700.13 MHz, CD_3OD) spectrum of compound **3a** 29
Figure S27. HSQC (700.13 MHz, CD_3OD) spectrum of compound **3a** 30
Figure S28. HMBC (700.13 MHz, CD_3OD) spectrum of compound **3a** 31
Figure S29. ROESY (700.13 MHz, CD_3OD) spectrum of compound **3a** 32

Figure S30. HRESIMS spectra of compound **4** 33

Figure S31. ^{1}H NMR (700.13 MHz, CD_3OD) spectrum of compound **4** 34

Figure S32. ^{13}C NMR (176.04 MHz, CD_3OD) spectrum of compound **4** 35

Figure S33. COSY (700.13 MHz, CD_3OD) spectrum of compound **4** 36

Figure S34. HSQC (700.13 MHz, CD_3OD) spectrum of compound **4** 37

Figure S35. HMBC (700.13 MHz, CD_3OD) spectrum of compound **4** 38

Figure S36. ROESY (700.13 MHz, CD_3OD) spectrum of compound **4** 39

Figure S37. HRESIMS spectra of compound **5** 40

Figure S38. ^{1}H NMR (700.13 MHz, CD_3OD) spectrum of compound **5** 41

Figure S39. ^{13}C NMR (176.04 MHz, CD_3OD) spectrum of compound **5** 42

Figure S40. COSY (700.13 MHz, CD_3OD) spectrum of compound **5** 43

Figure S41. HSQC (700.13 MHz, CD_3OD) spectrum of compound **5** 44

Figure S42. HMBC (700.13 MHz, CD_3OD) spectrum of compound **5** 45

Figure S43. ROESY (700.13 MHz, CD_3OD) spectrum of compound **5** 46

Figure S1. HRESIMS spectra of compound **1**.

(–)HRESIMS $[M – Na]^{–}$ ion

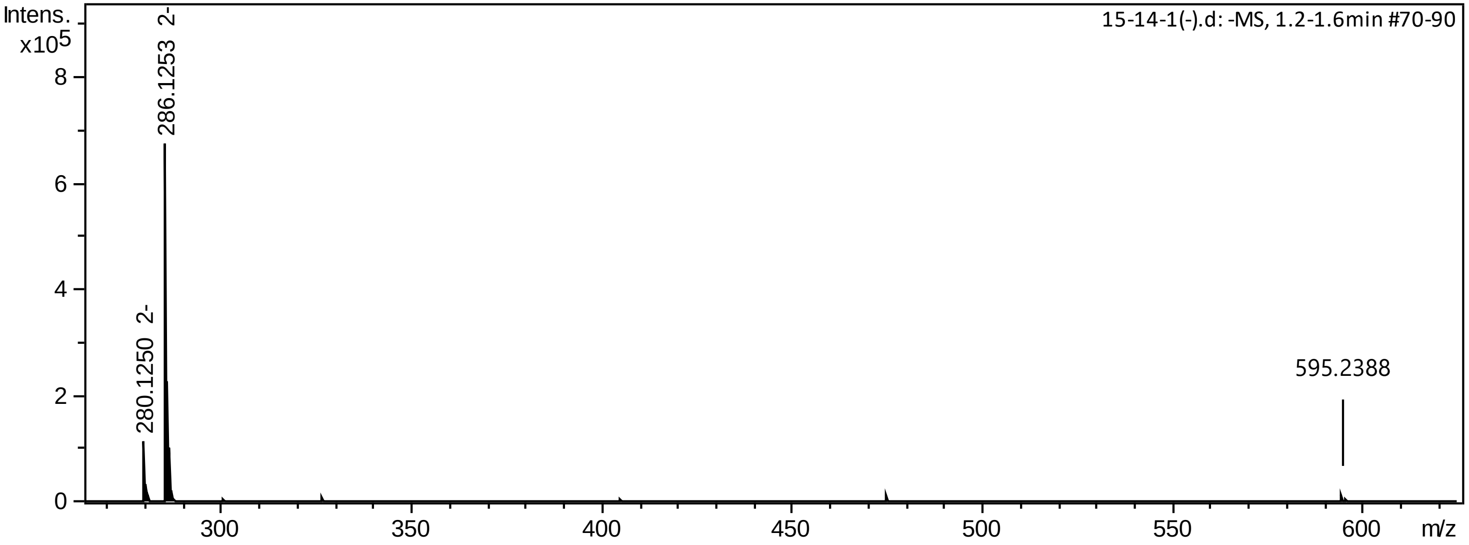

(+)HRESIMS $[M + Na]^{+}$ ion

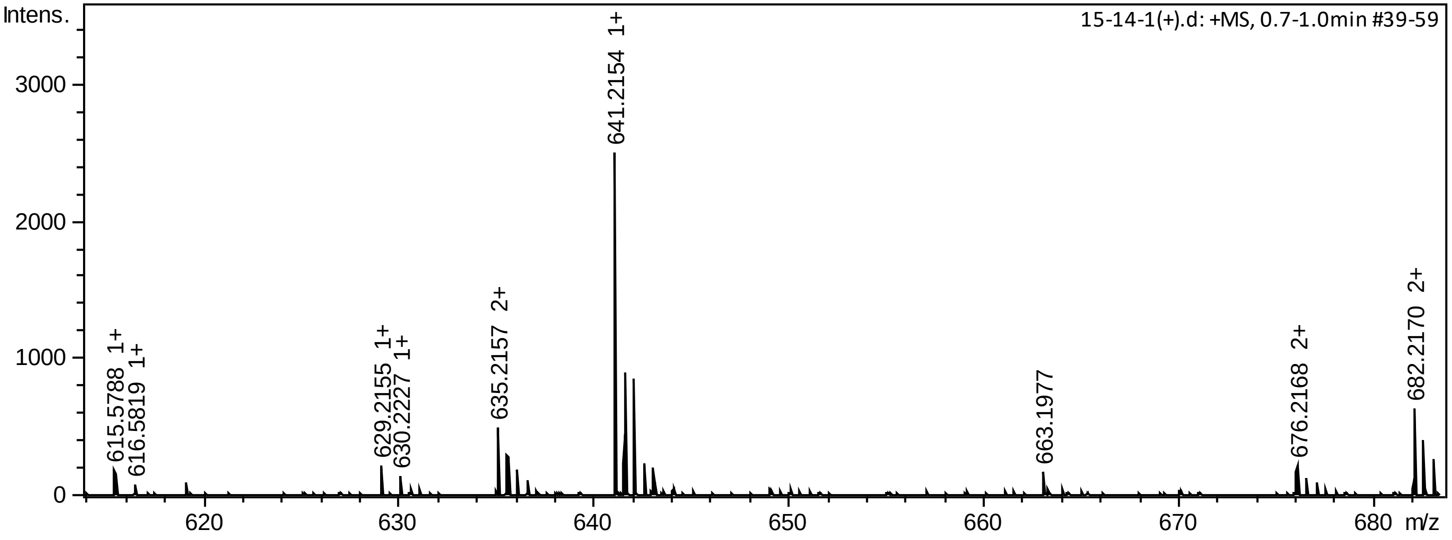

Figure S2. ^{1}H NMR (700.13 MHz, CD_3OD) spectrum of compound **1**.

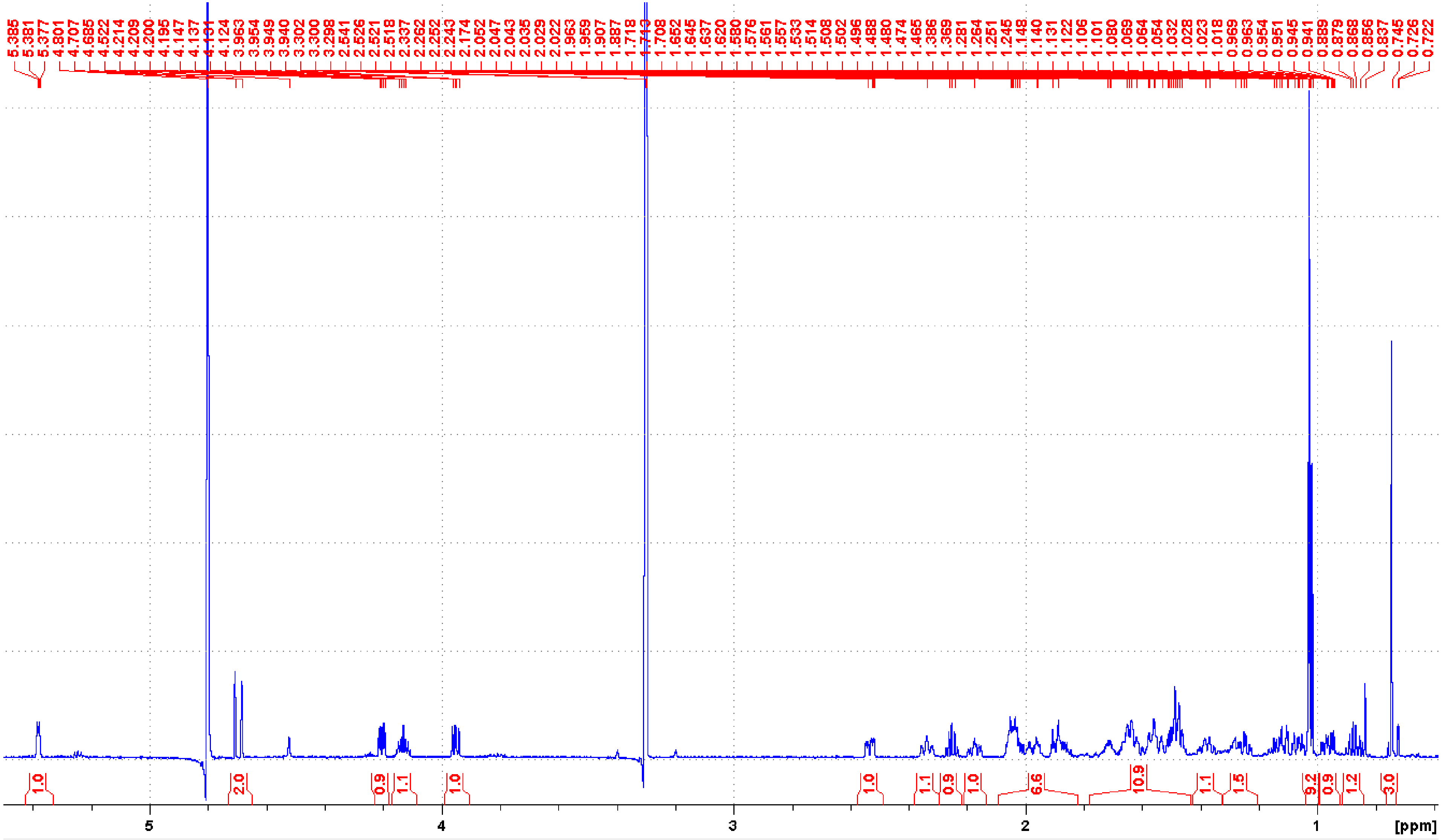

Figure S3. ^{13}C NMR (176.04 MHz, CD_3OD) spectrum of compound **1**.

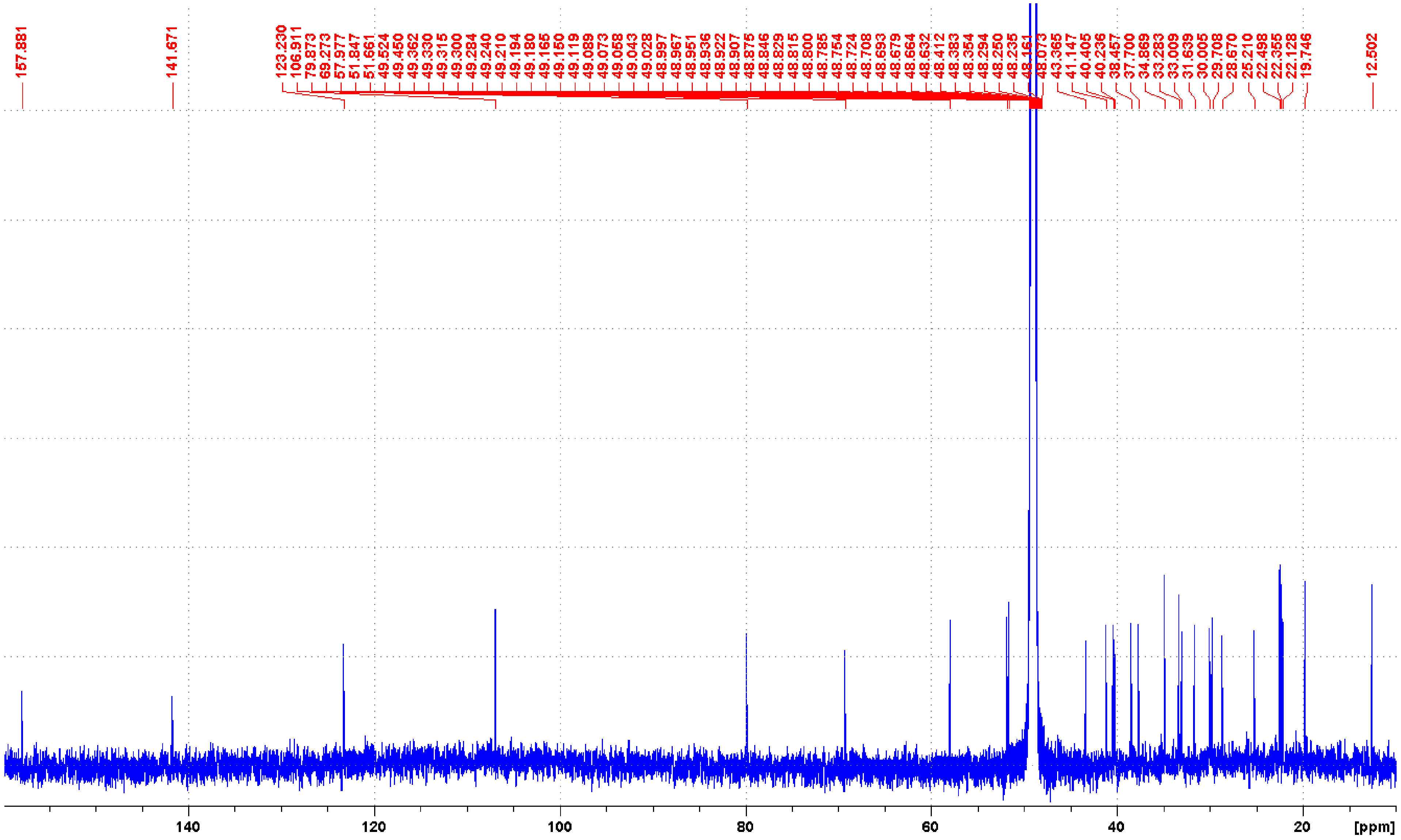

Figure S4. COSY (700.13 MHz, CD_3OD) spectrum of compound **1**.

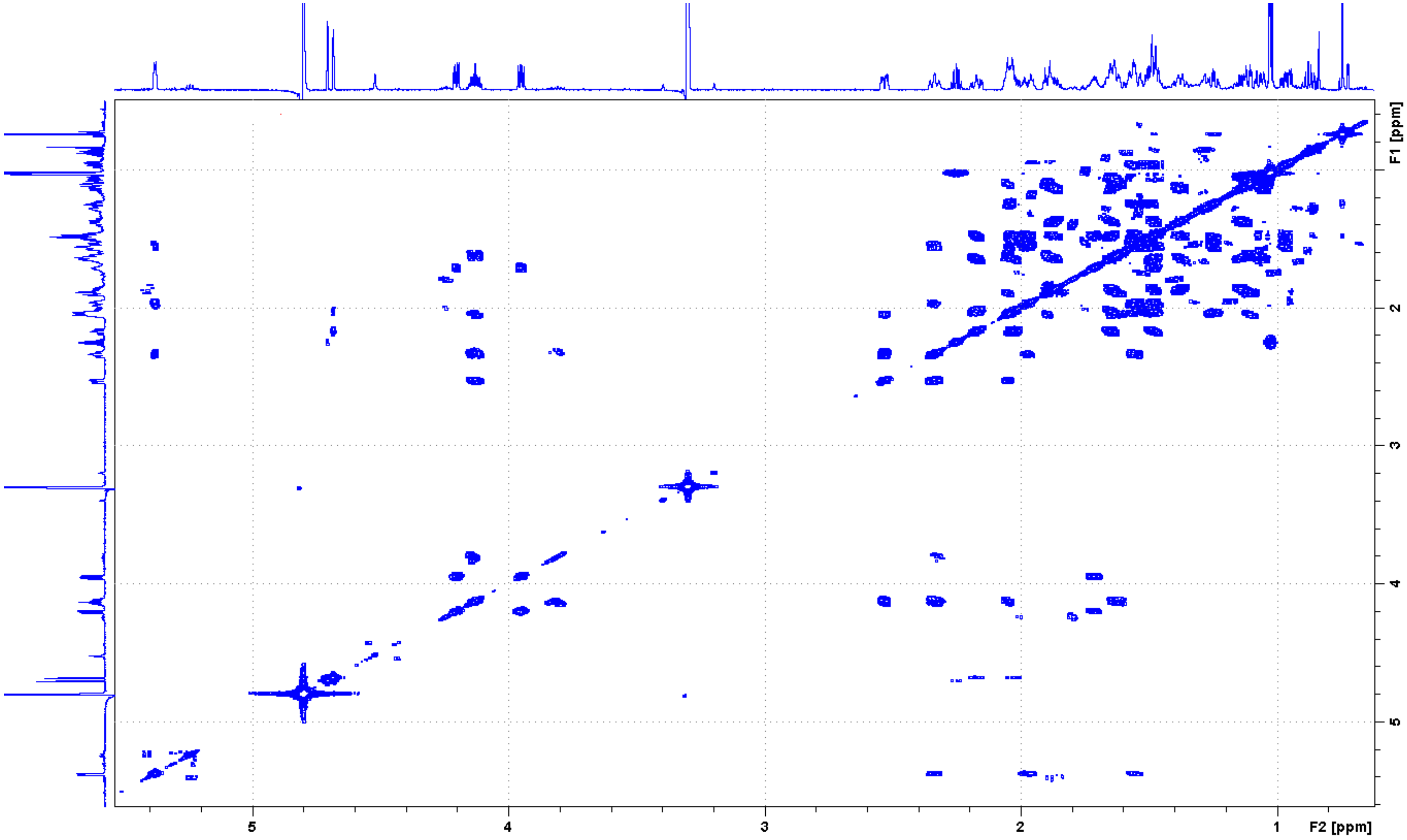

Figure S5. HSQC (700.13 MHz, CD_3OD) spectrum of compound **1**.

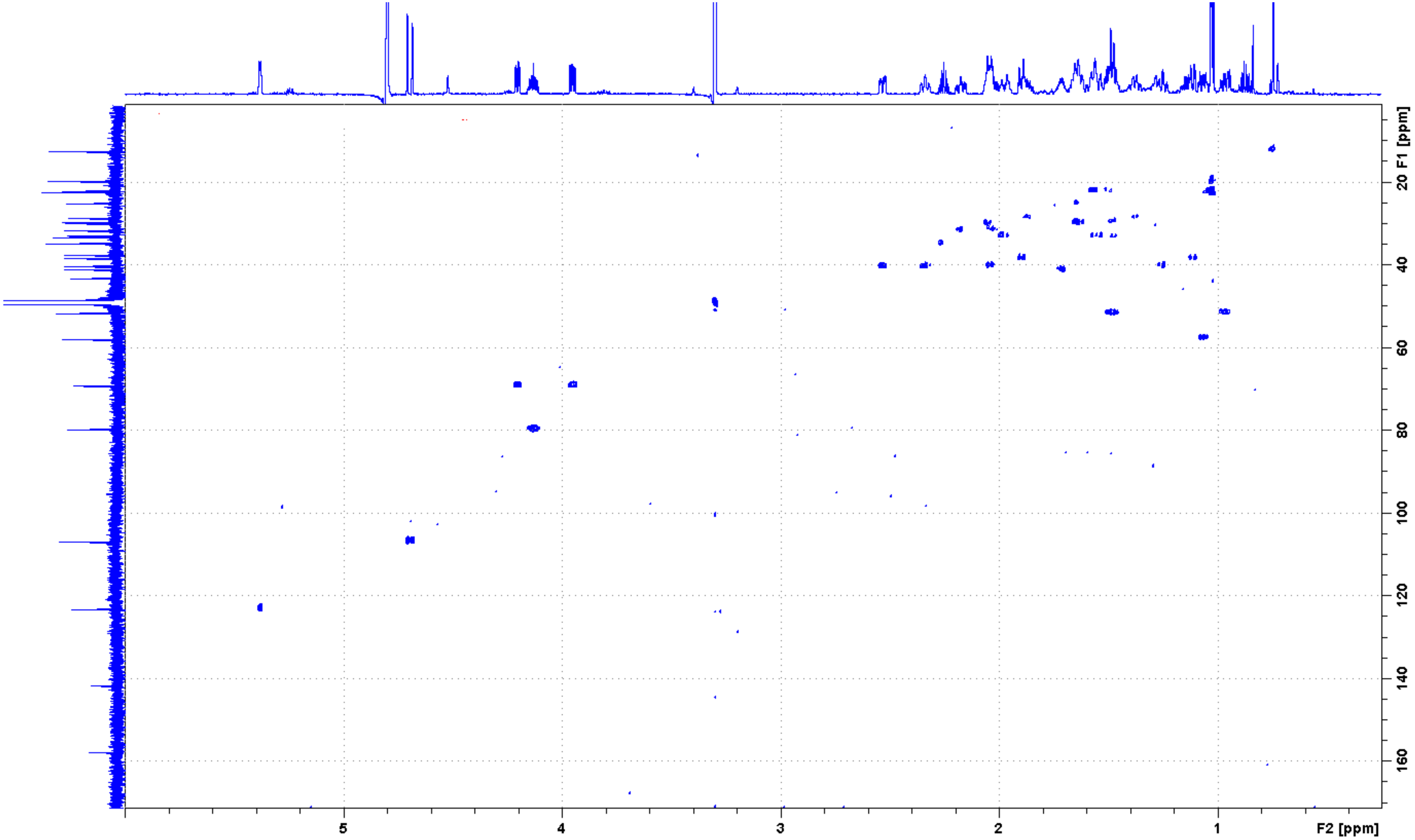

Figure S6. HMBC (700.13 MHz, CD_3OD) spectrum of compound **1**.

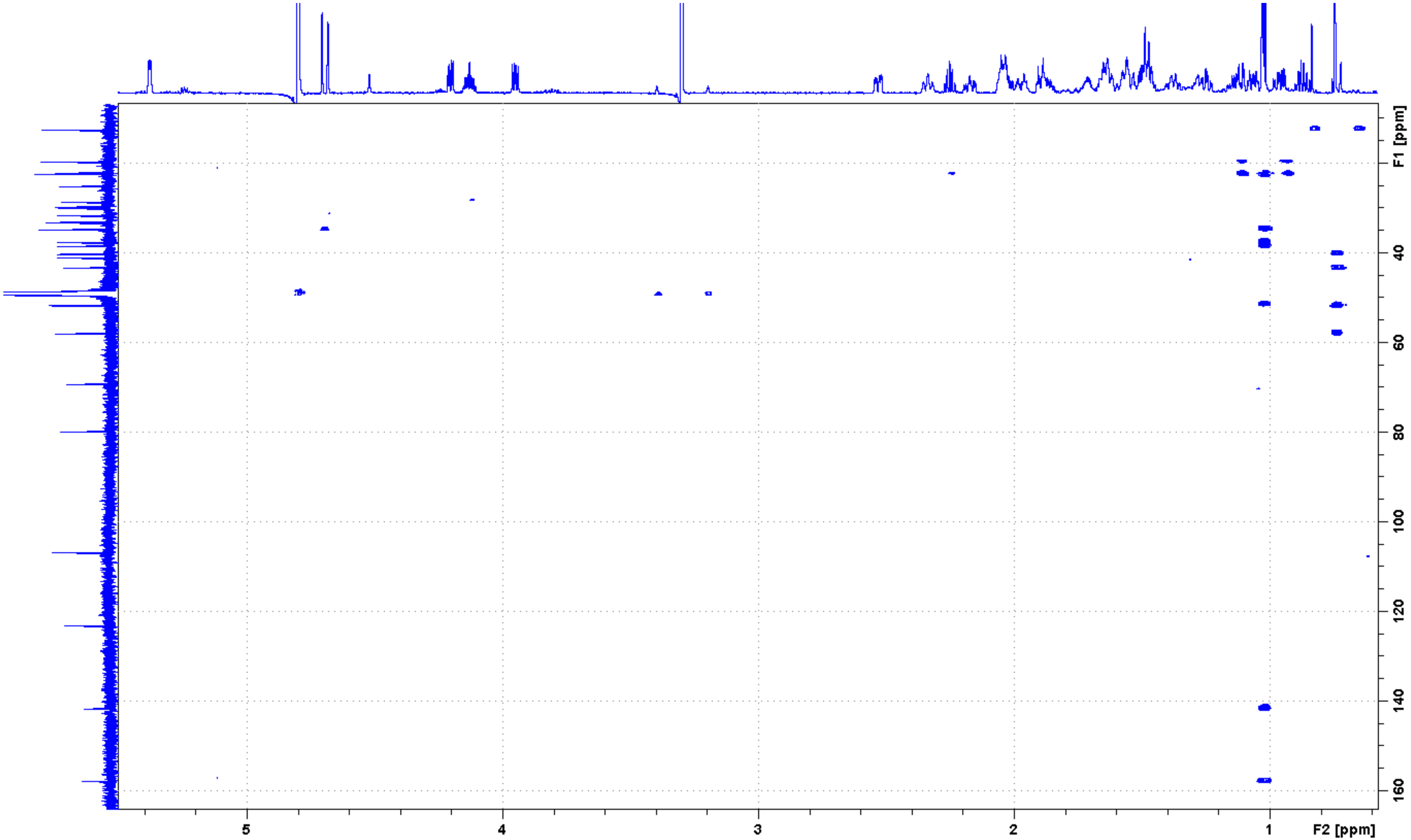

Figure S7. ROESY (700.13 MHz, CD_3OD) spectrum of compound **1**.

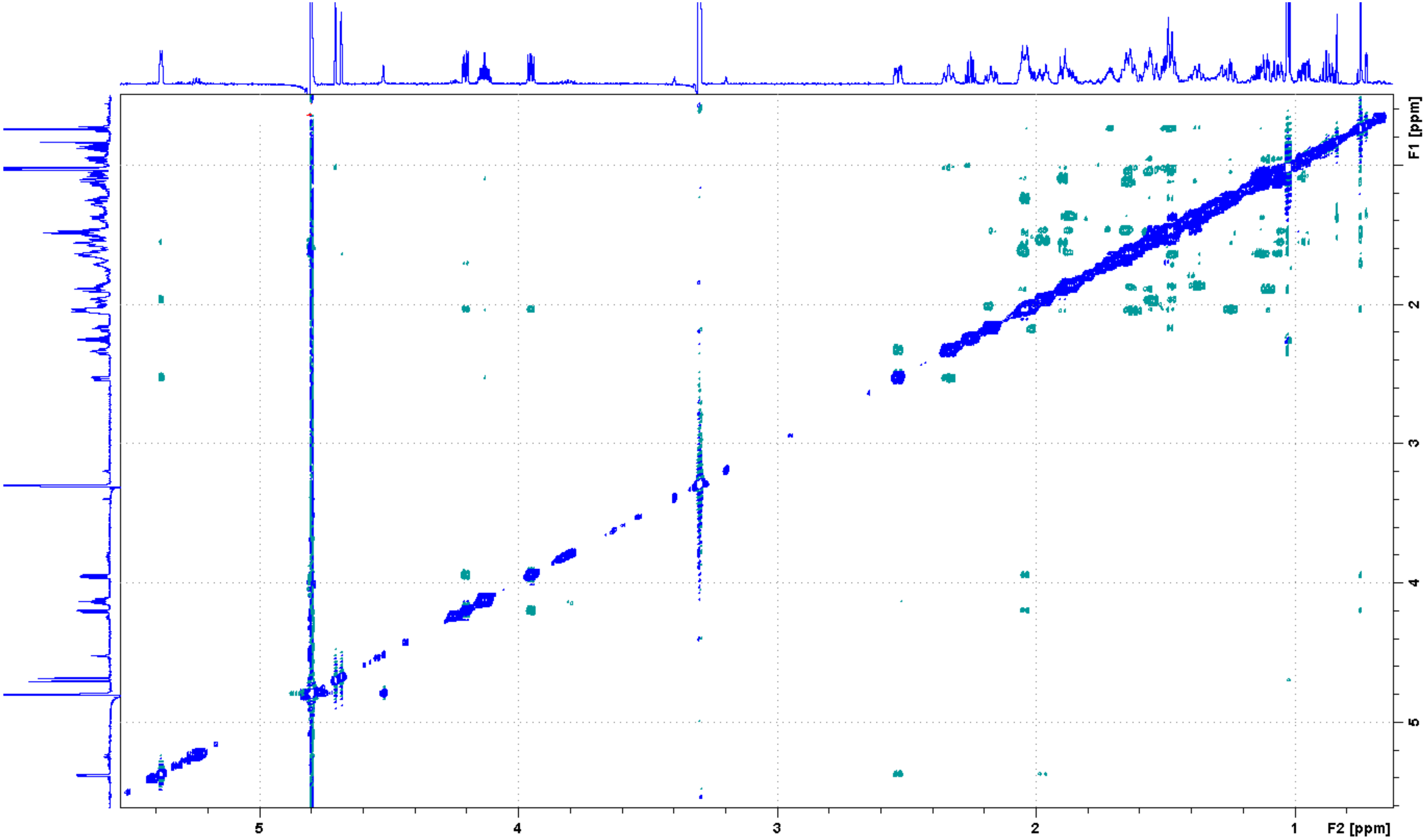

Figure S8. ^{1}H NMR (700.13 MHz, CD_3OD) spectrum of the mixture of **2** and **3**.

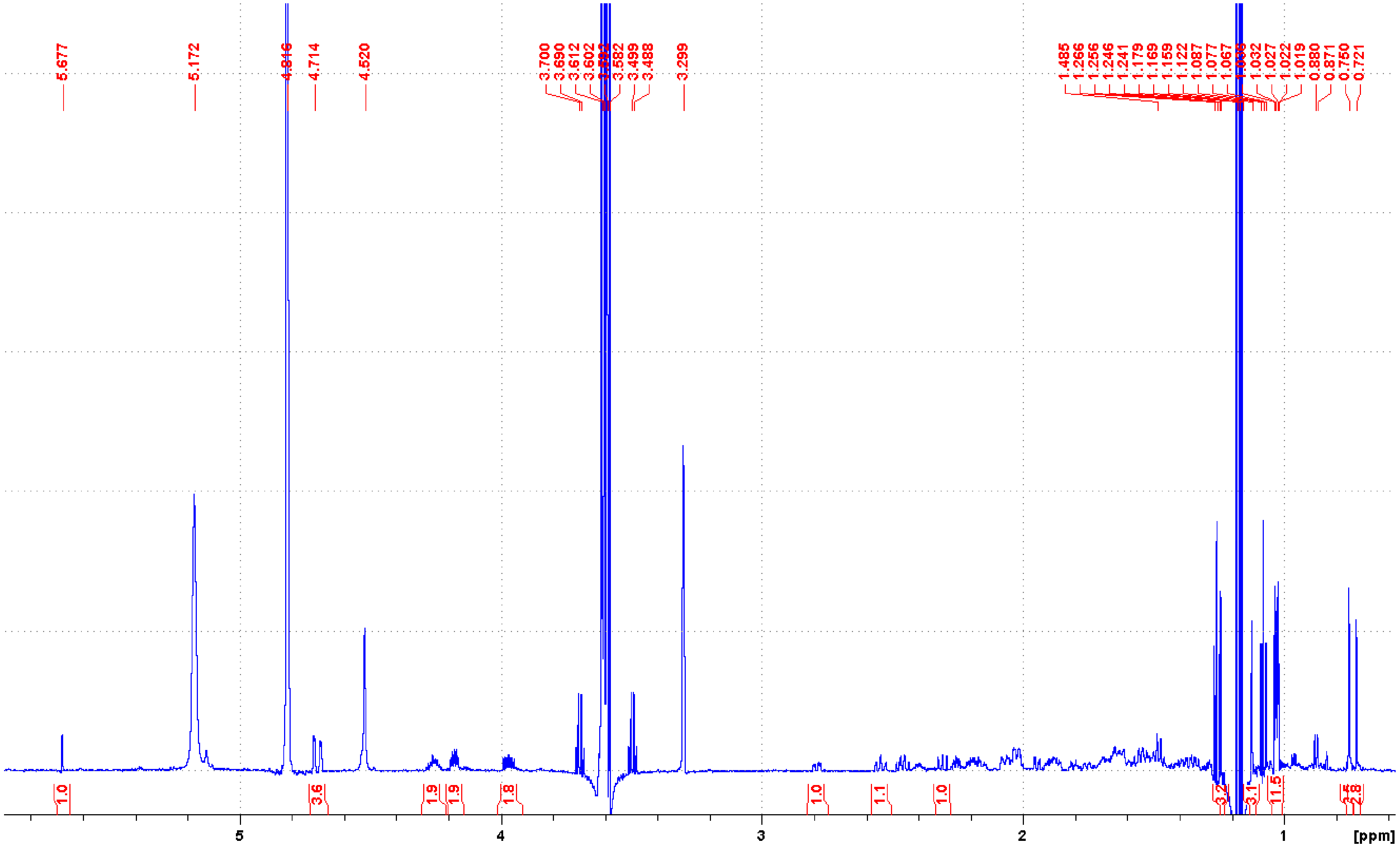

Figure S9. ^{13}C NMR (176.04 MHz, CD_3OD) spectrum of the mixture of **2** and **3**.

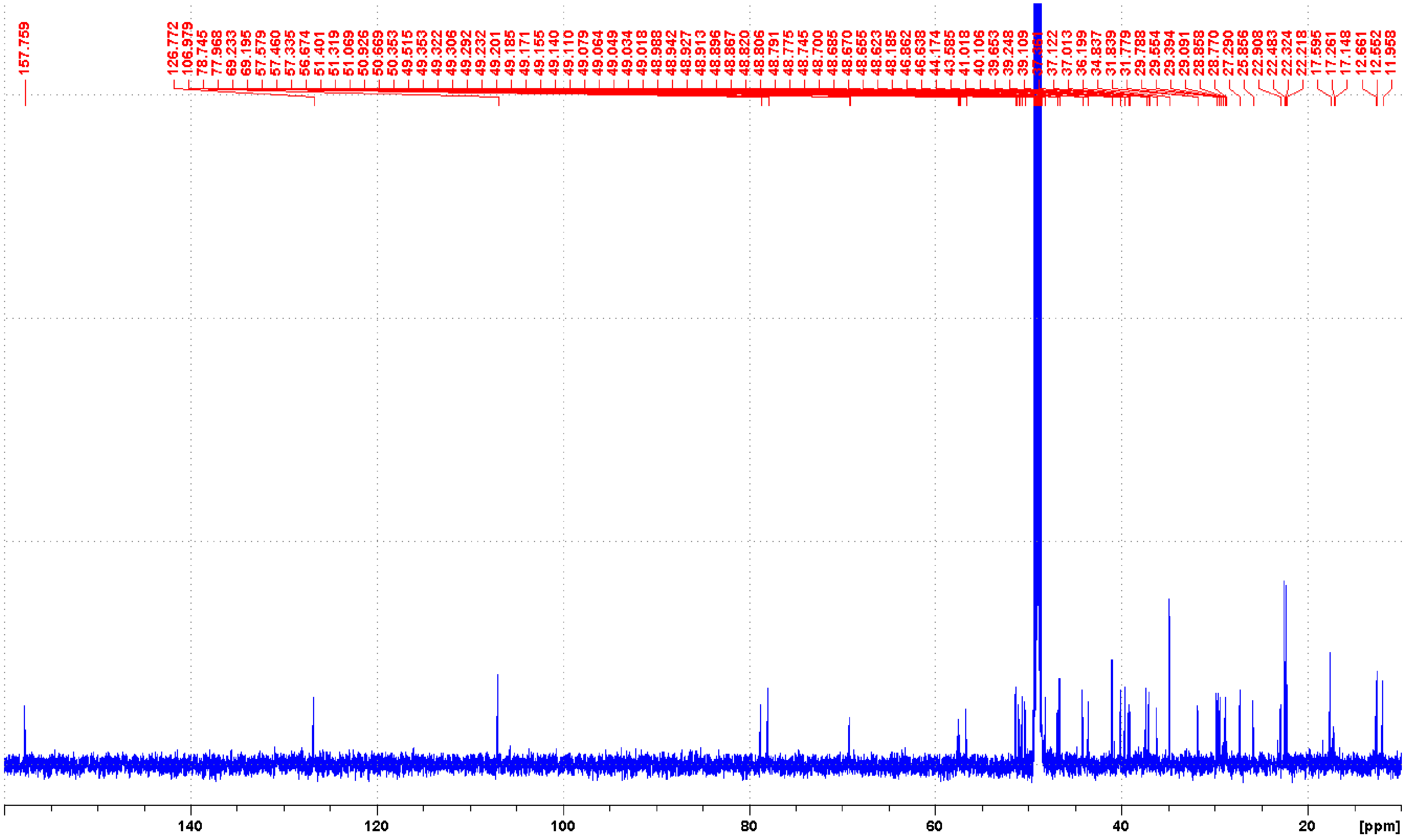

Figure S10. COSY (700.13 MHz, CD_3OD) spectrum of the mixture of **2** and **3**.

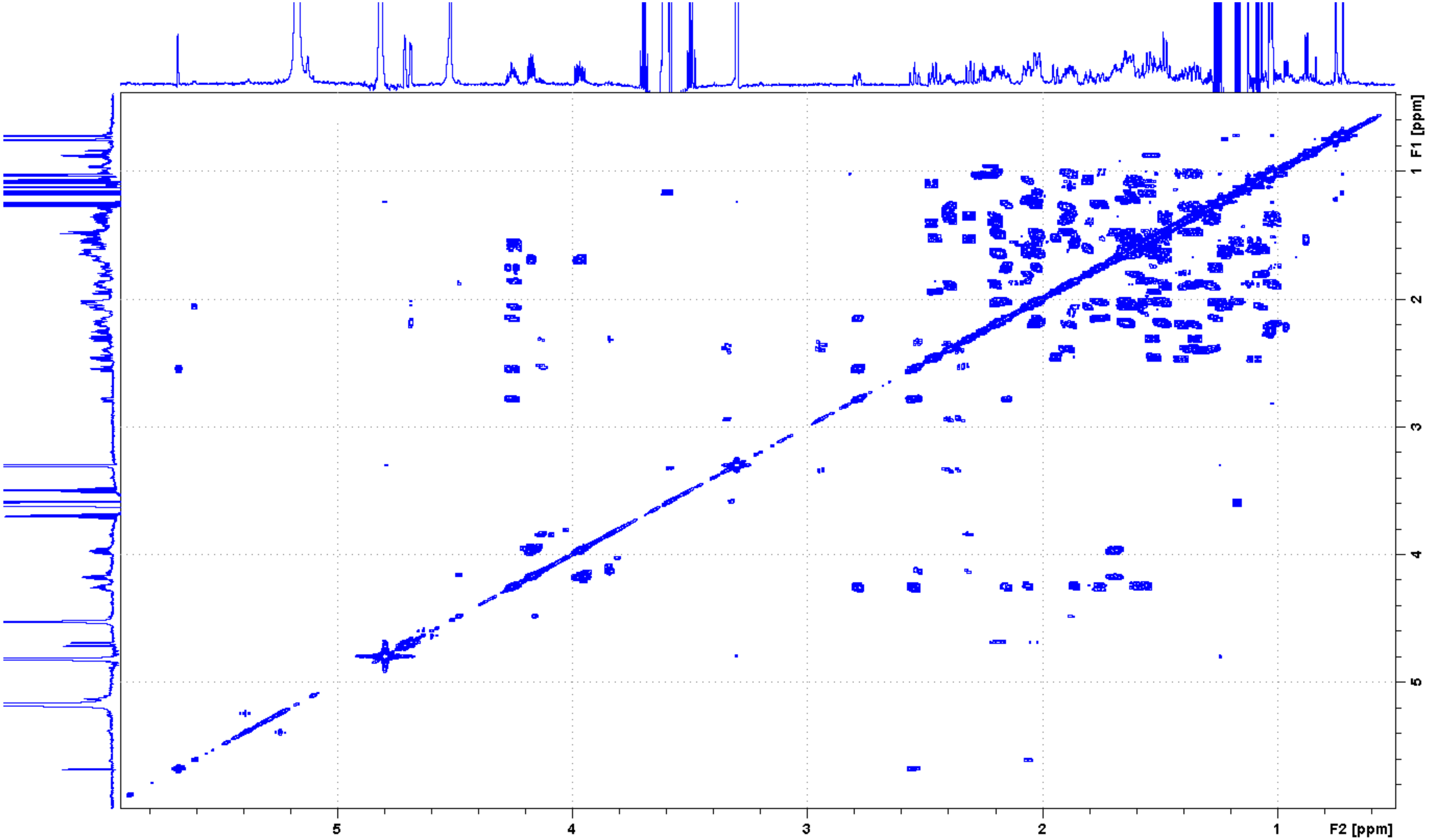

Figure S11. HSQC (700.13 MHz, CD_3OD) spectrum of the mixture of **2** and **3**.

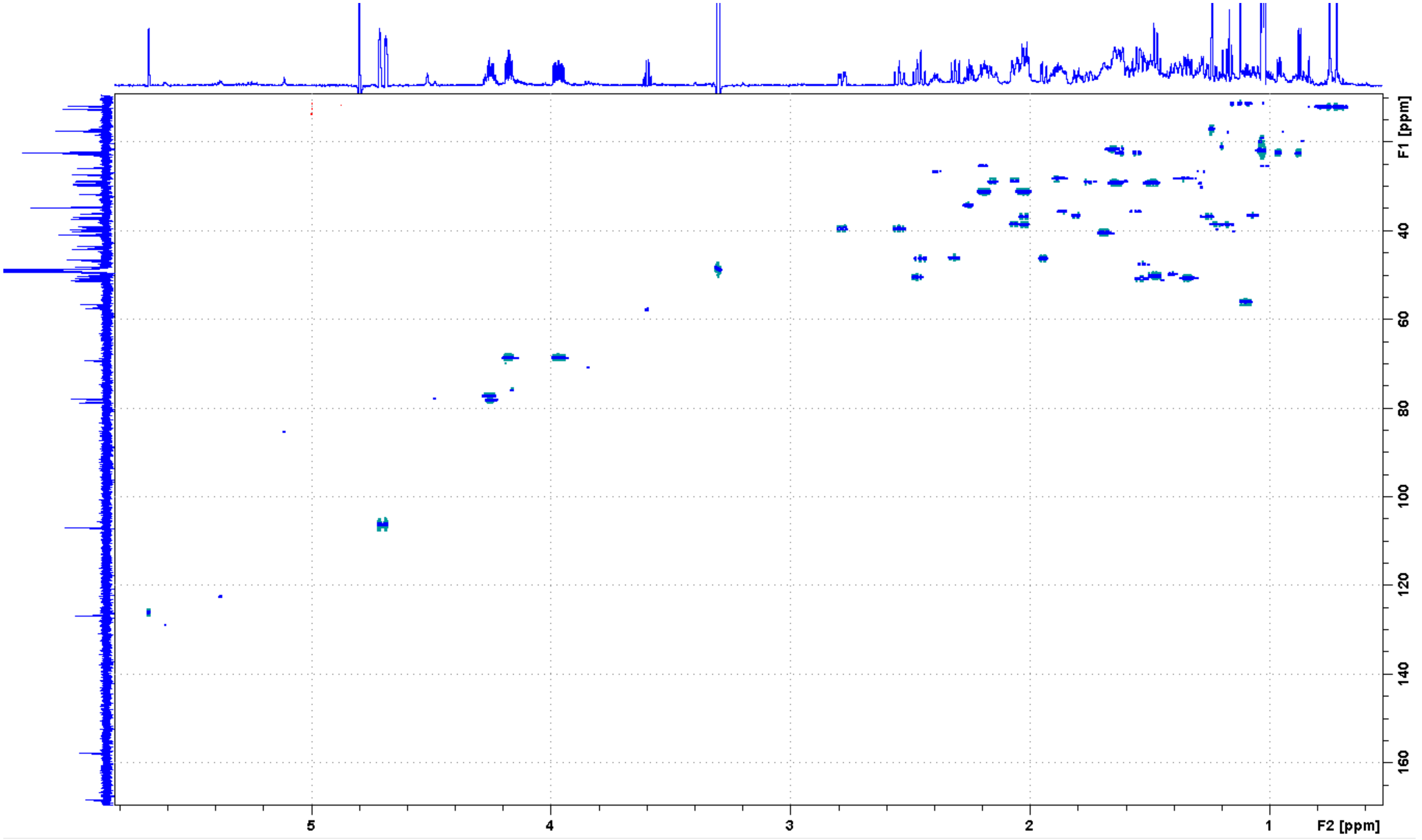

Figure S12. HMBC (700.13 MHz, CD_3OD) spectrum of the mixture of **2** and **3**.

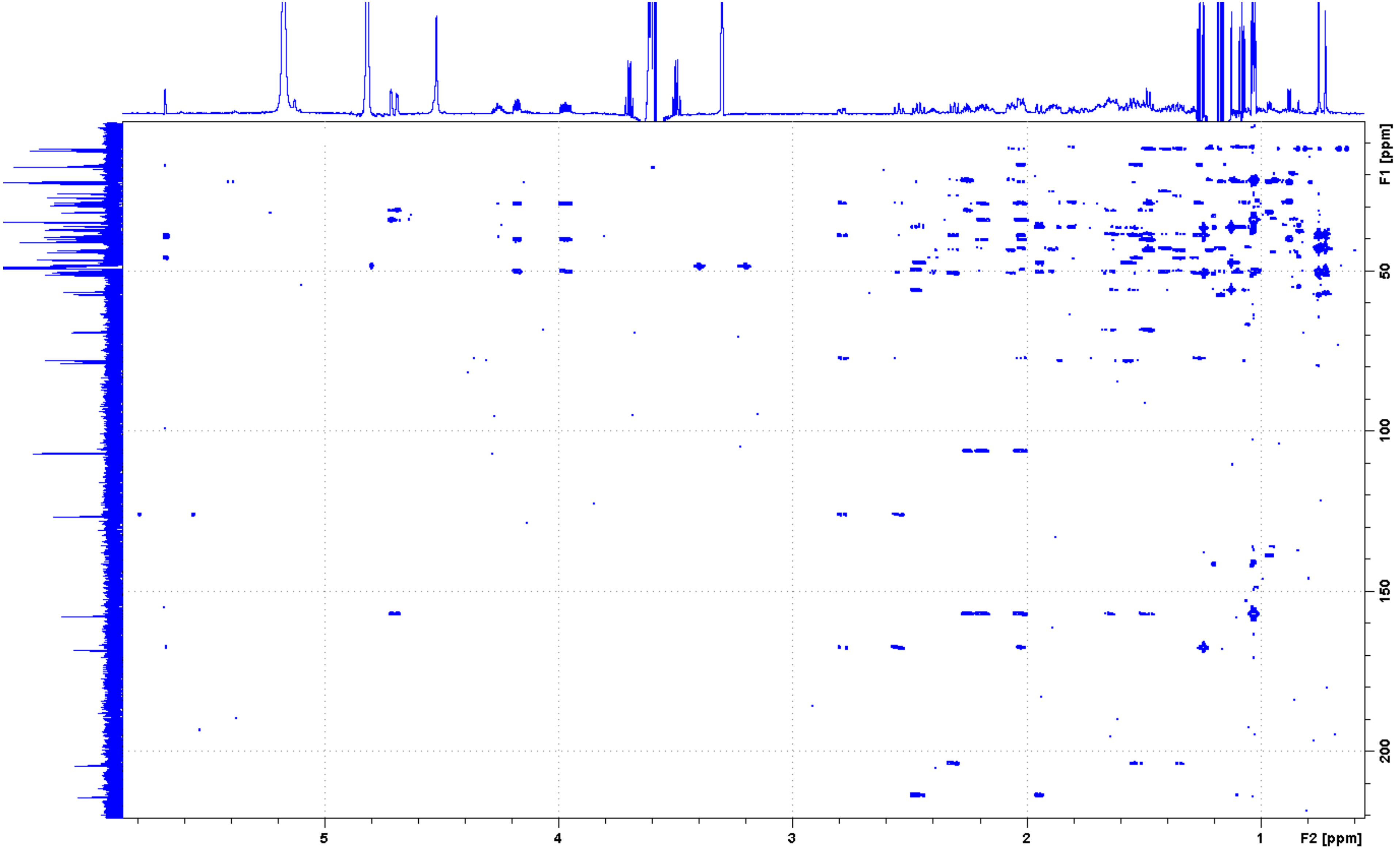

Figure S13. ROESY (700.13 MHz, CD_3OD) spectrum of the mixture of **2** and **3**.

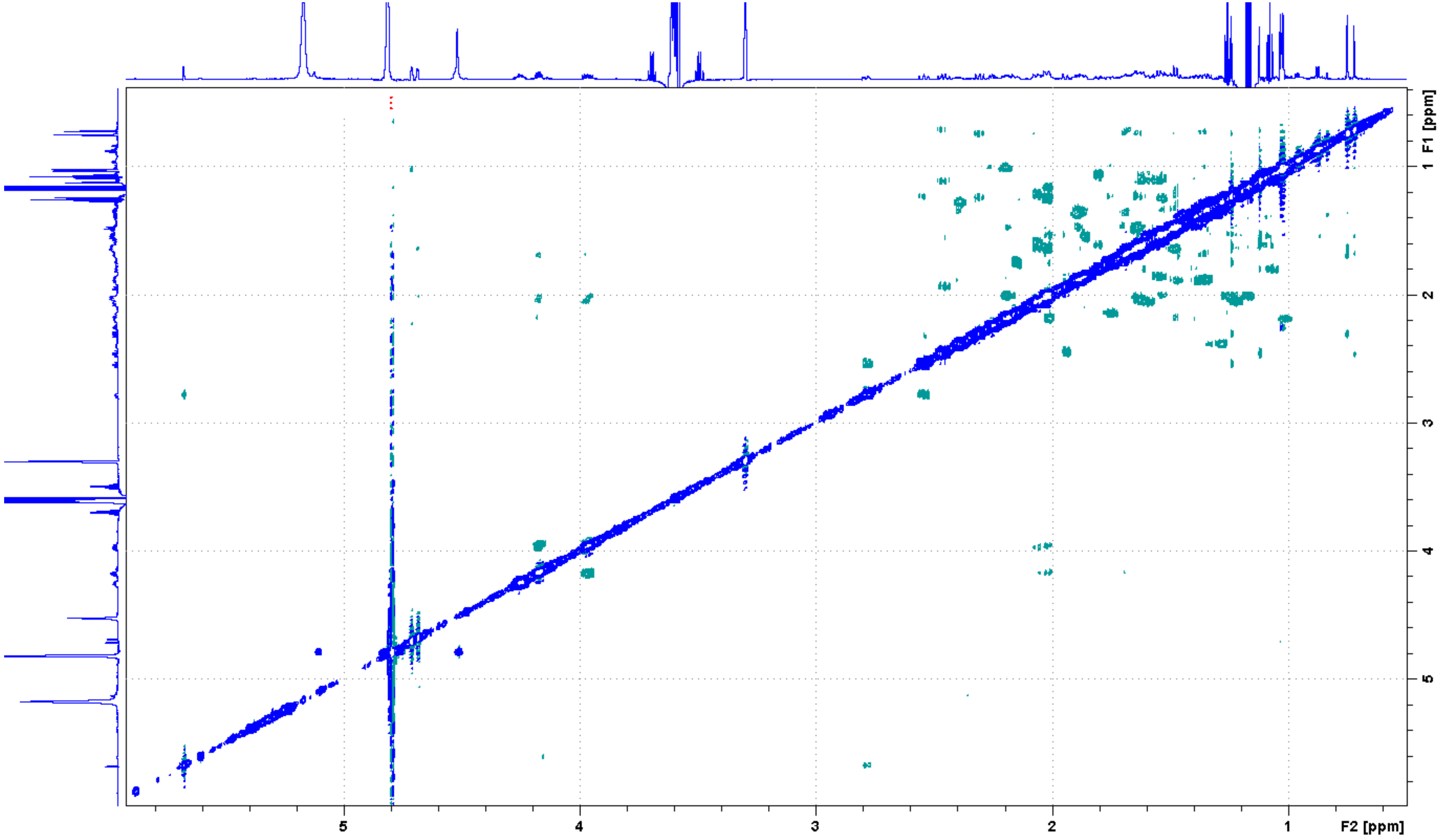

Figure S14. HRESIMS spectra of the mixture of **2** and **3**.

(–)HRESIMS [M – Na]⁻ ion

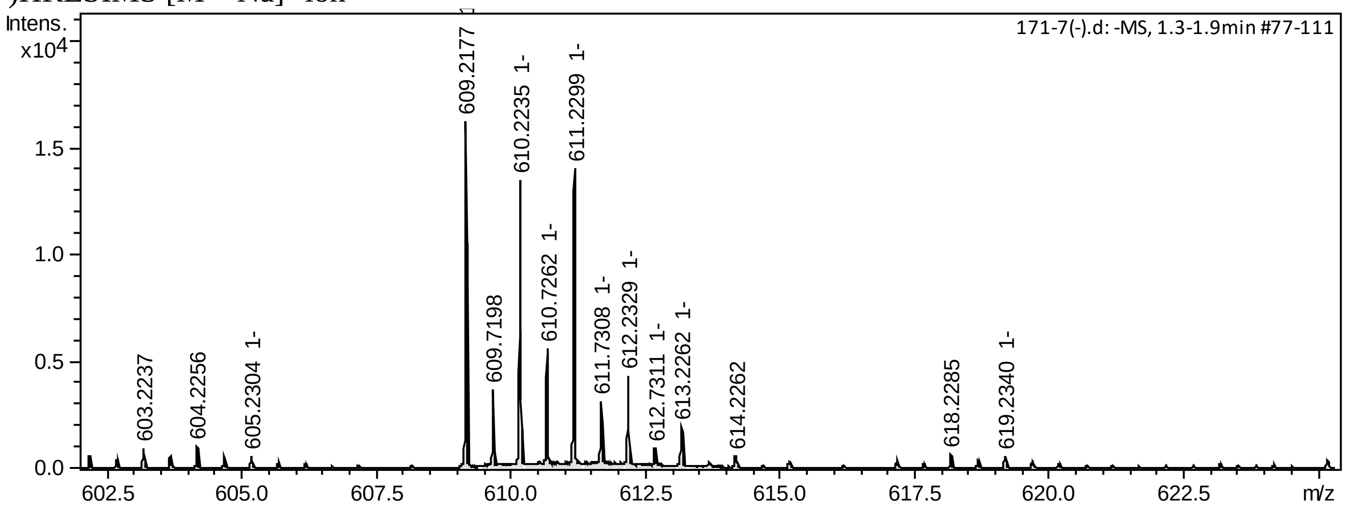

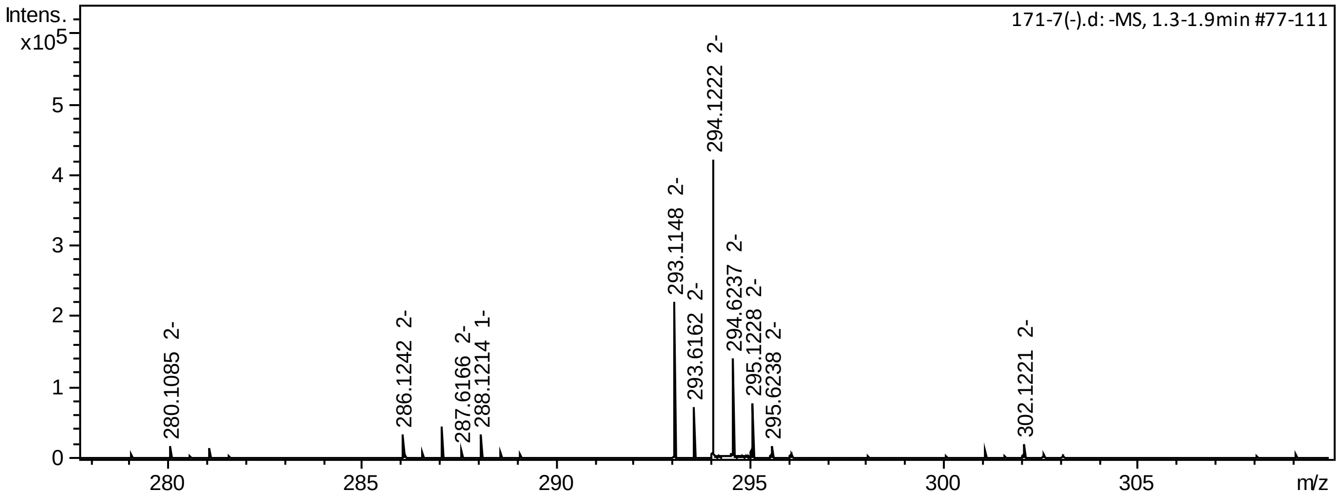

(+)HRESIMS [M + Na]⁺ ion

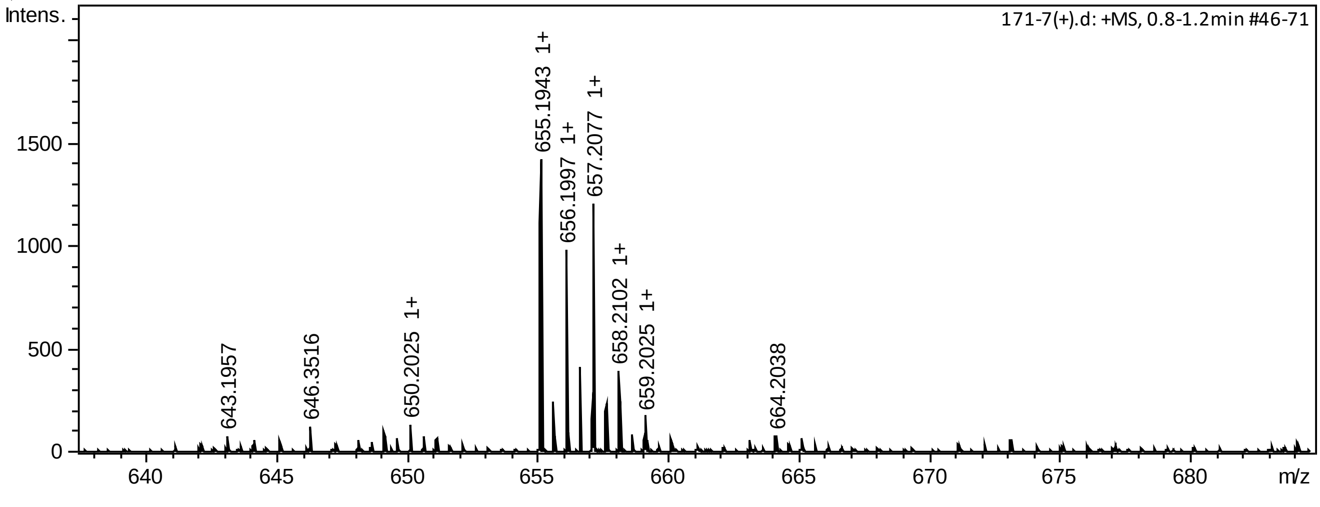

Figure S15. HRESIMS spectra of compound **2a**.

(–)HRESIMS [M – Na]$^-$ ion

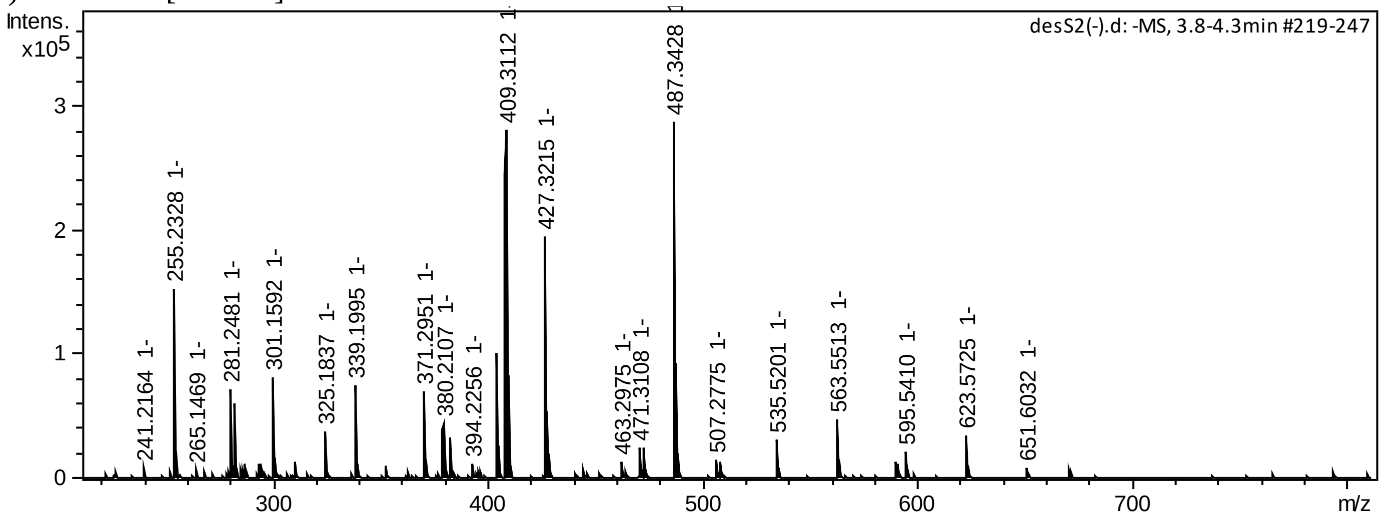

(+)HRESIMS [M + Na]$^+$ ion

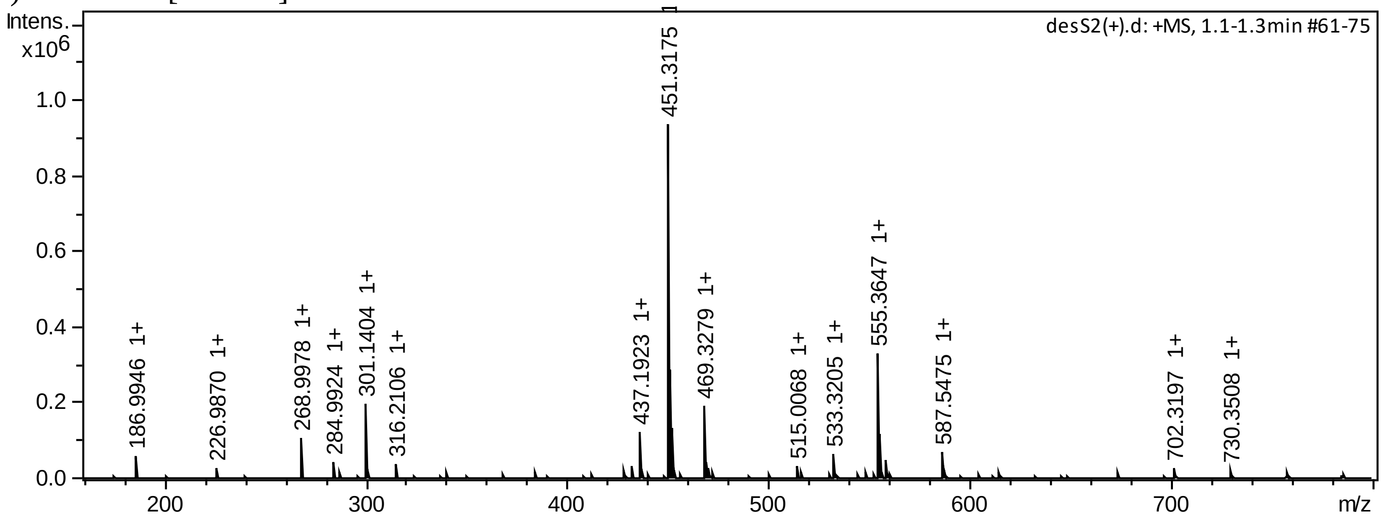

Figure S16. HRESIMS spectra of compound **3a**.

(–)HRESIMS [M – Na]$^{–}$ ion

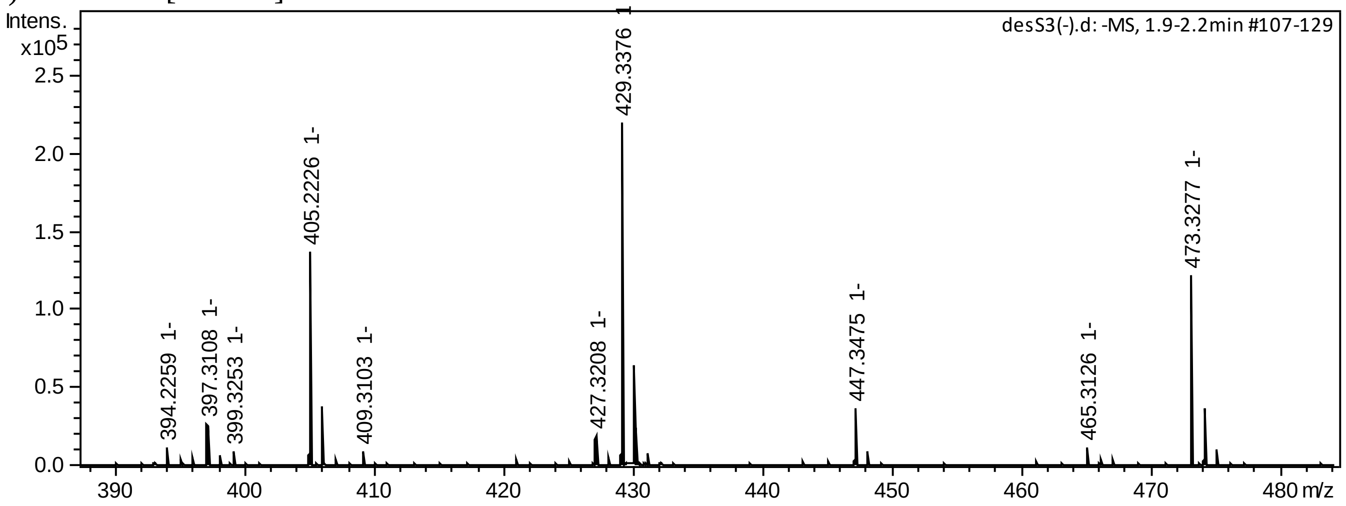

(+)HRESIMS [M + Na]$^{+}$ ion

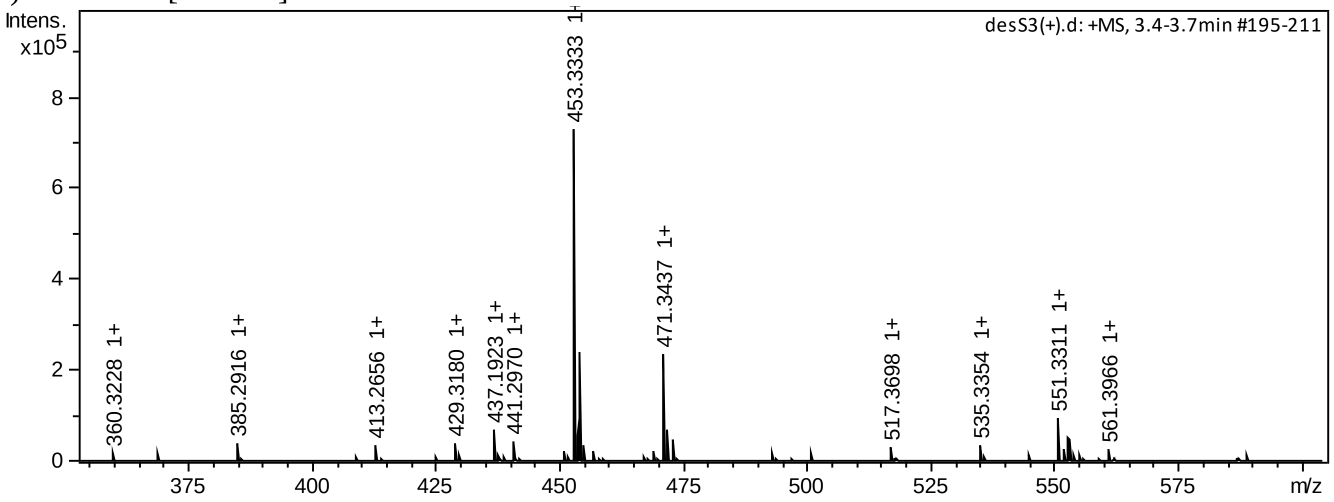

Figure S17. (**A**) COSY and key HMBC correlations of the mixture of compounds **2a** and **3a**. (**B**) Key ROESY correlations of the mixture of compounds **2a** and **3a**.

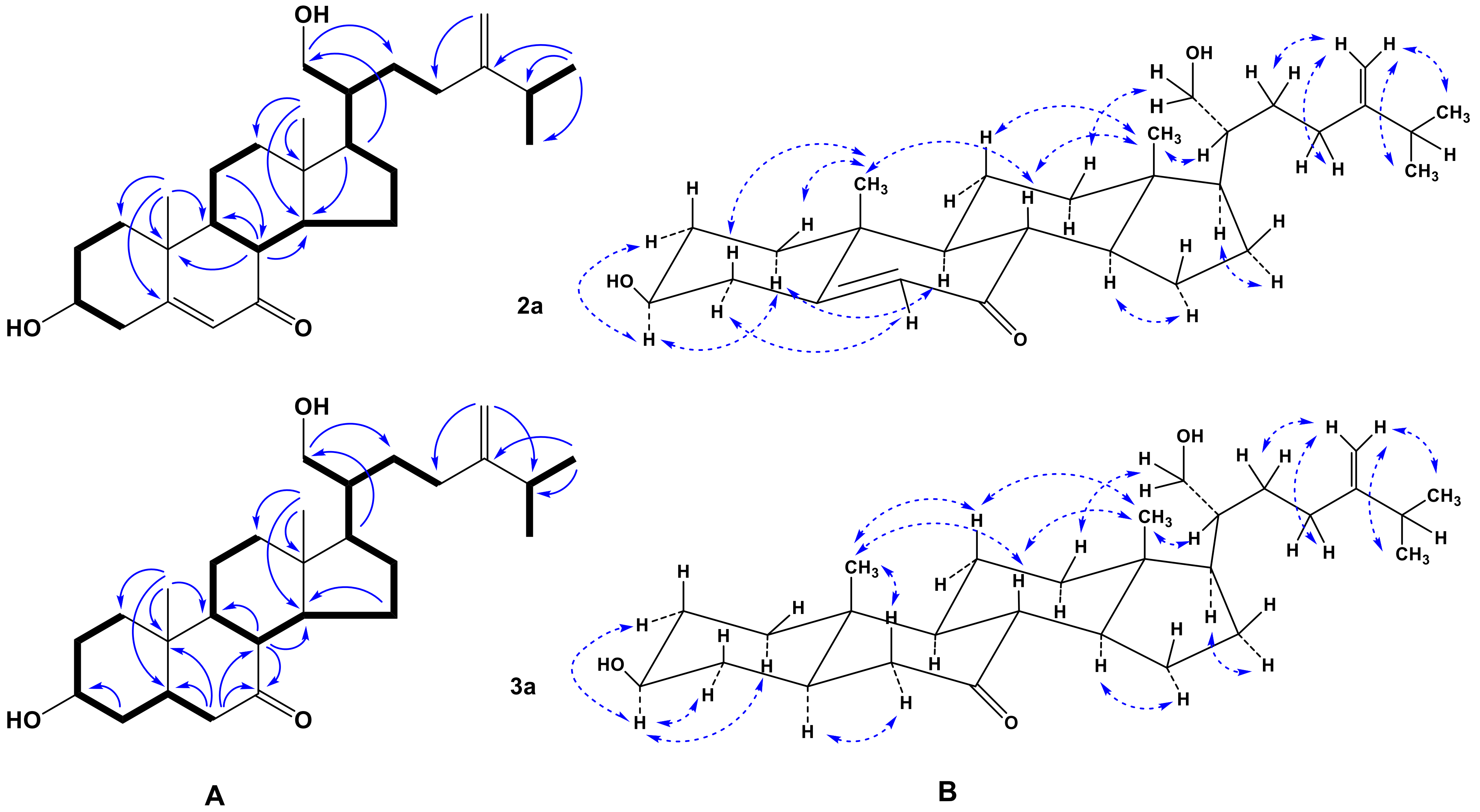

Figure S18. ^{1}H NMR (700.13 MHz, CD_3OD) spectrum of compound **2a**.

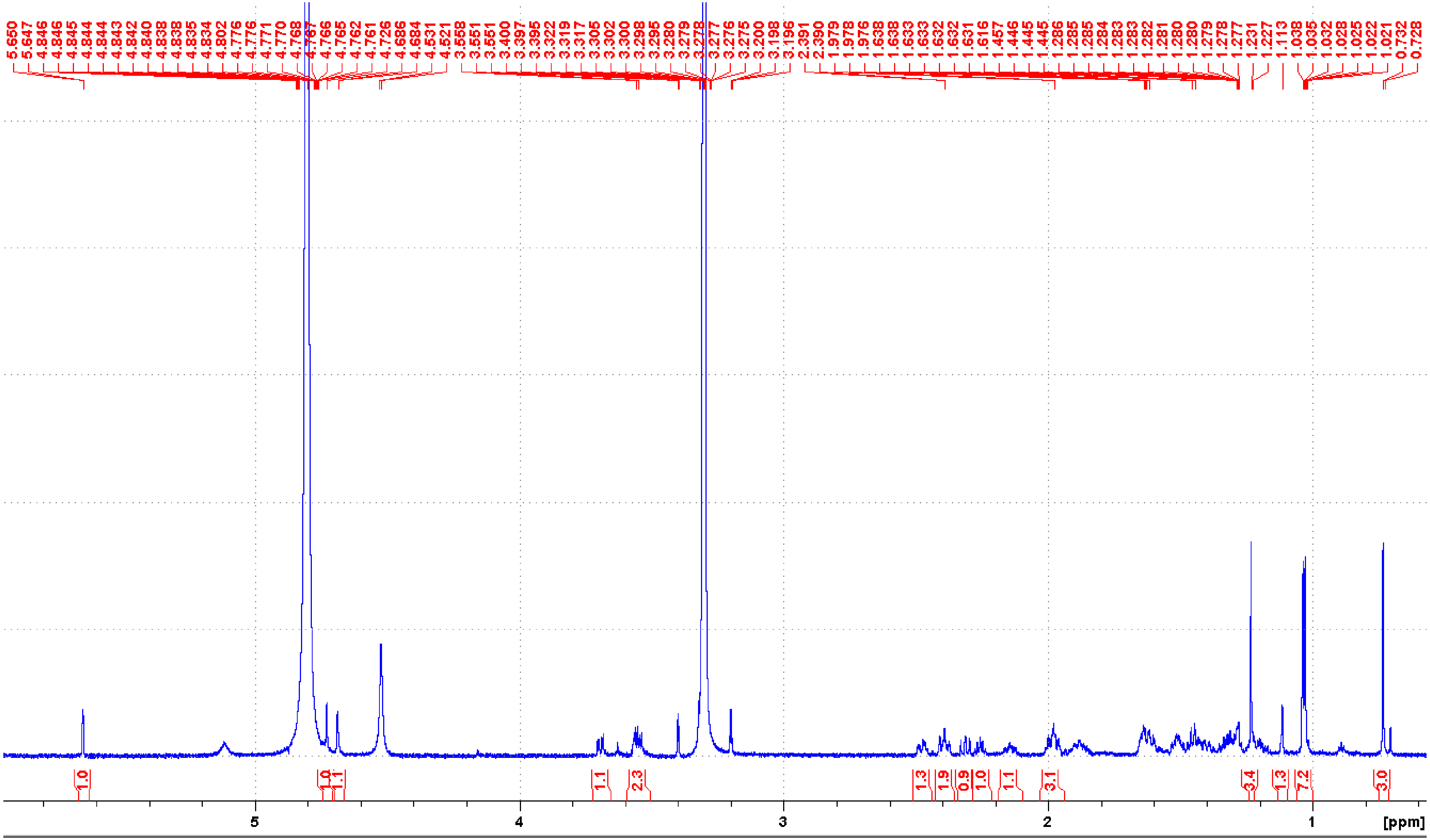

Figure S19. ^{13}C NMR (176.04 MHz, CD_3OD) spectrum of compound **2a**.

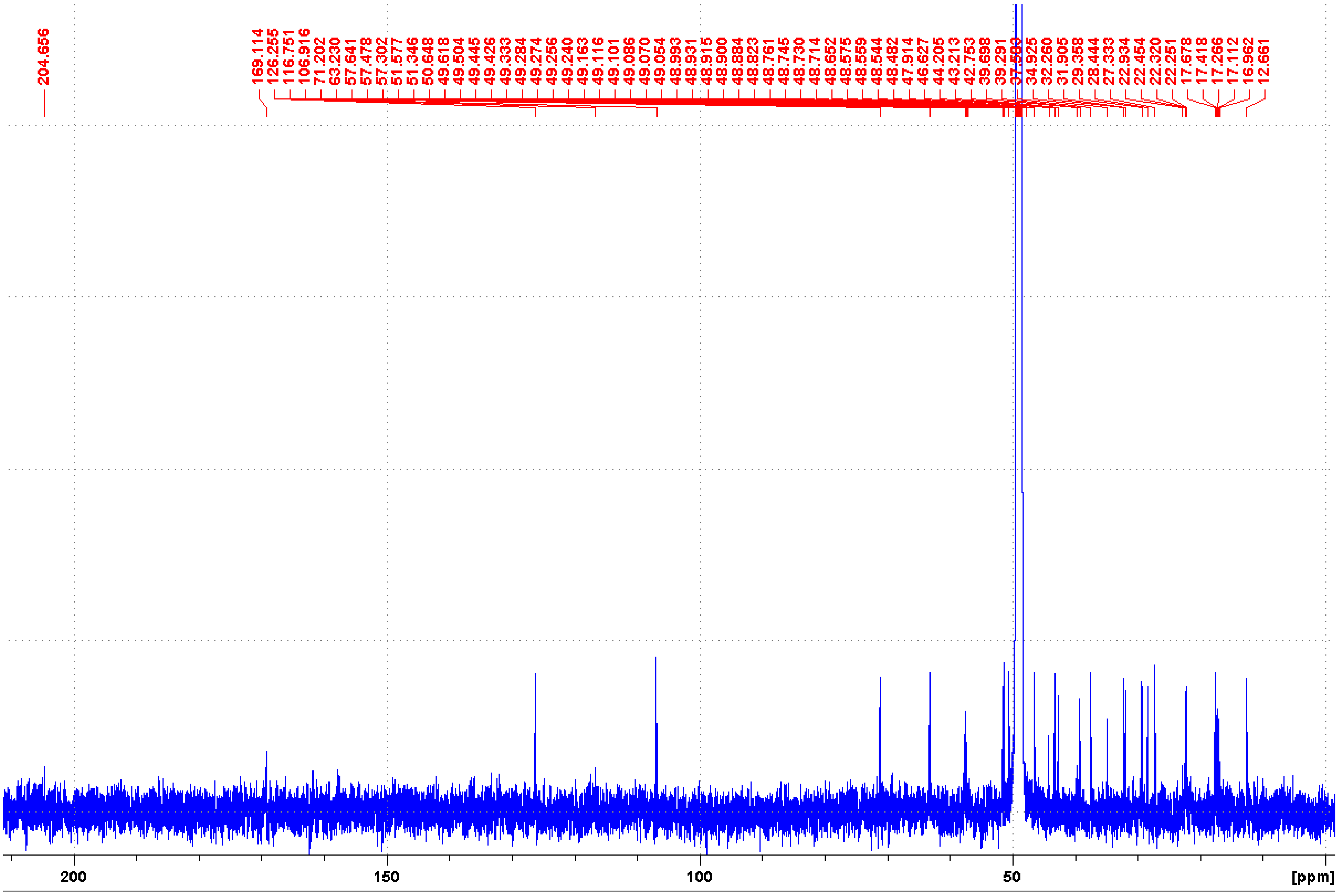

Figure S20. COSY (700.13 MHz, CD_3OD) spectrum of compound **2a**.

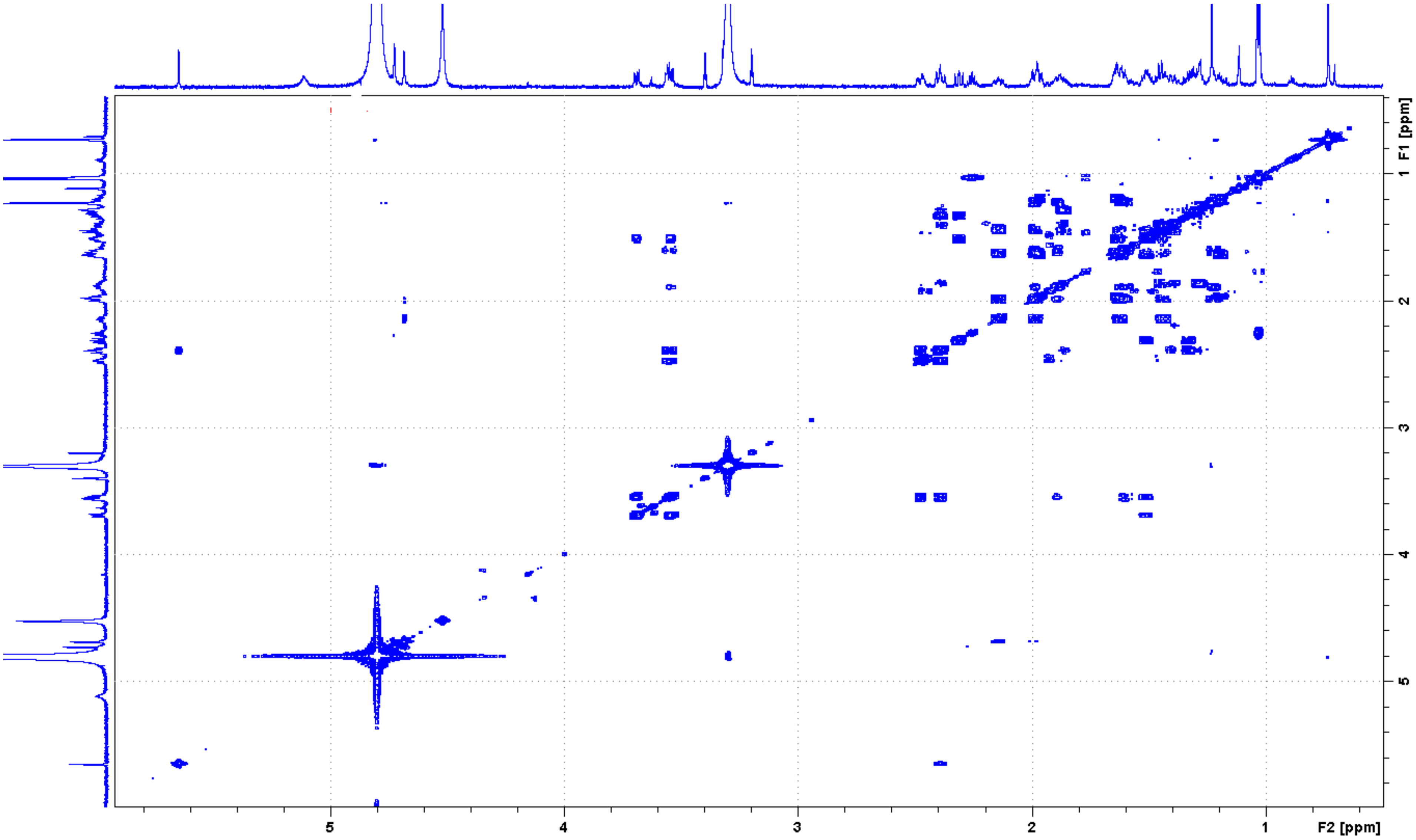

Figure S21. HSQC (700.13 MHz, CD_3OD) spectrum of compound **2a**.

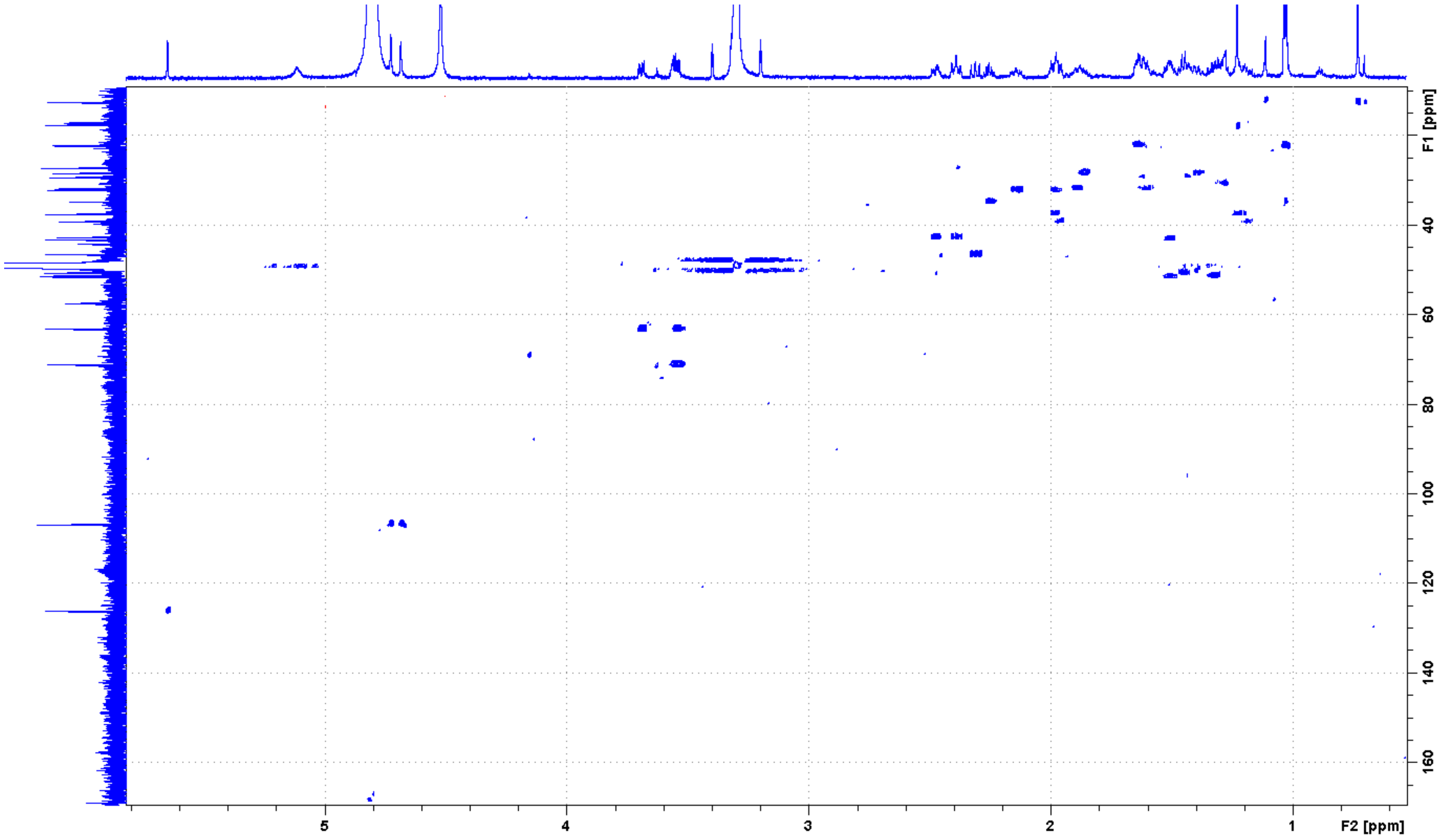

Figure S22. HMBC (700.13 MHz, CD_3OD) spectrum of compound **2a**.

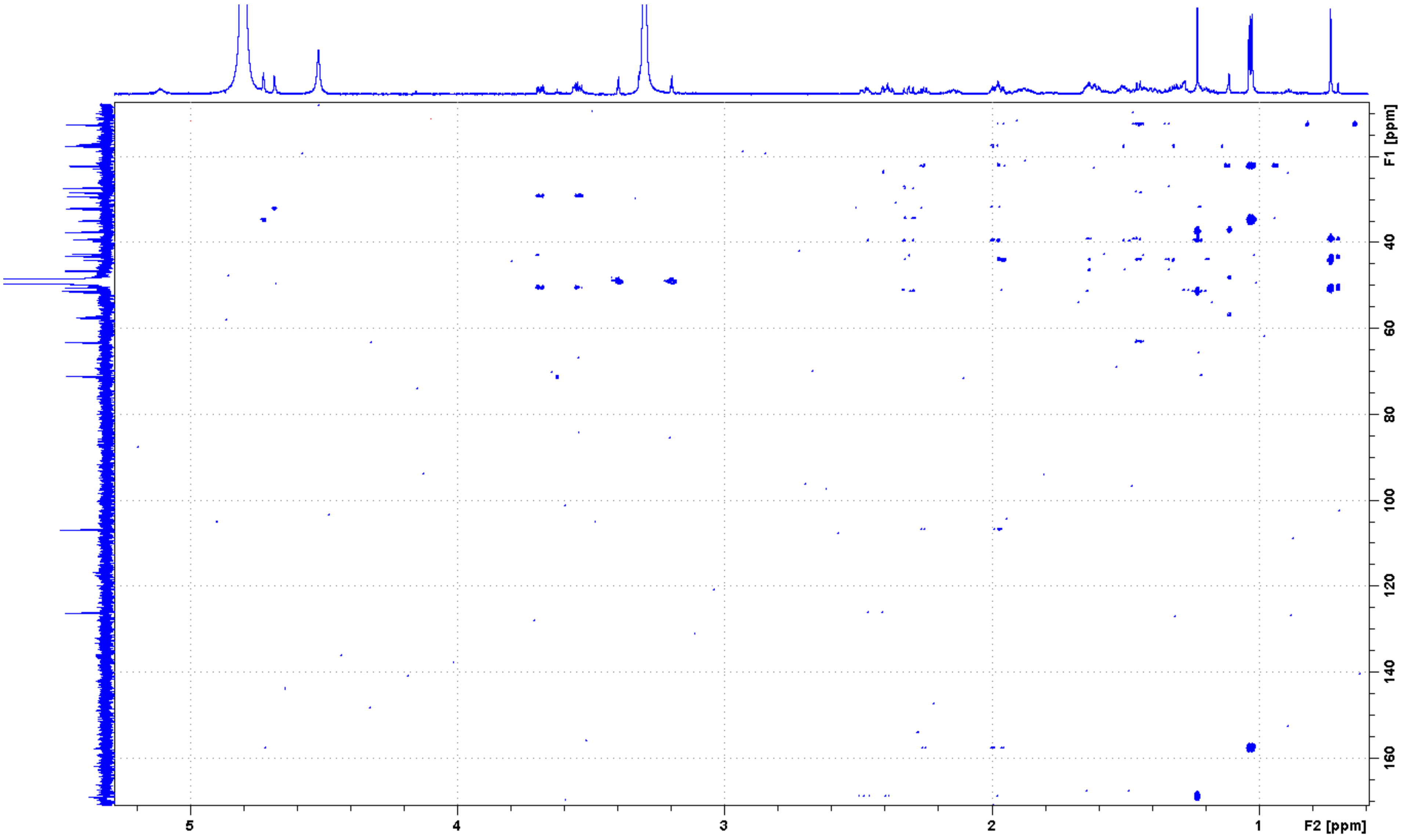

Figure S23. ROESY (700.13 MHz, CD_3OD) spectrum of compound **2a**.

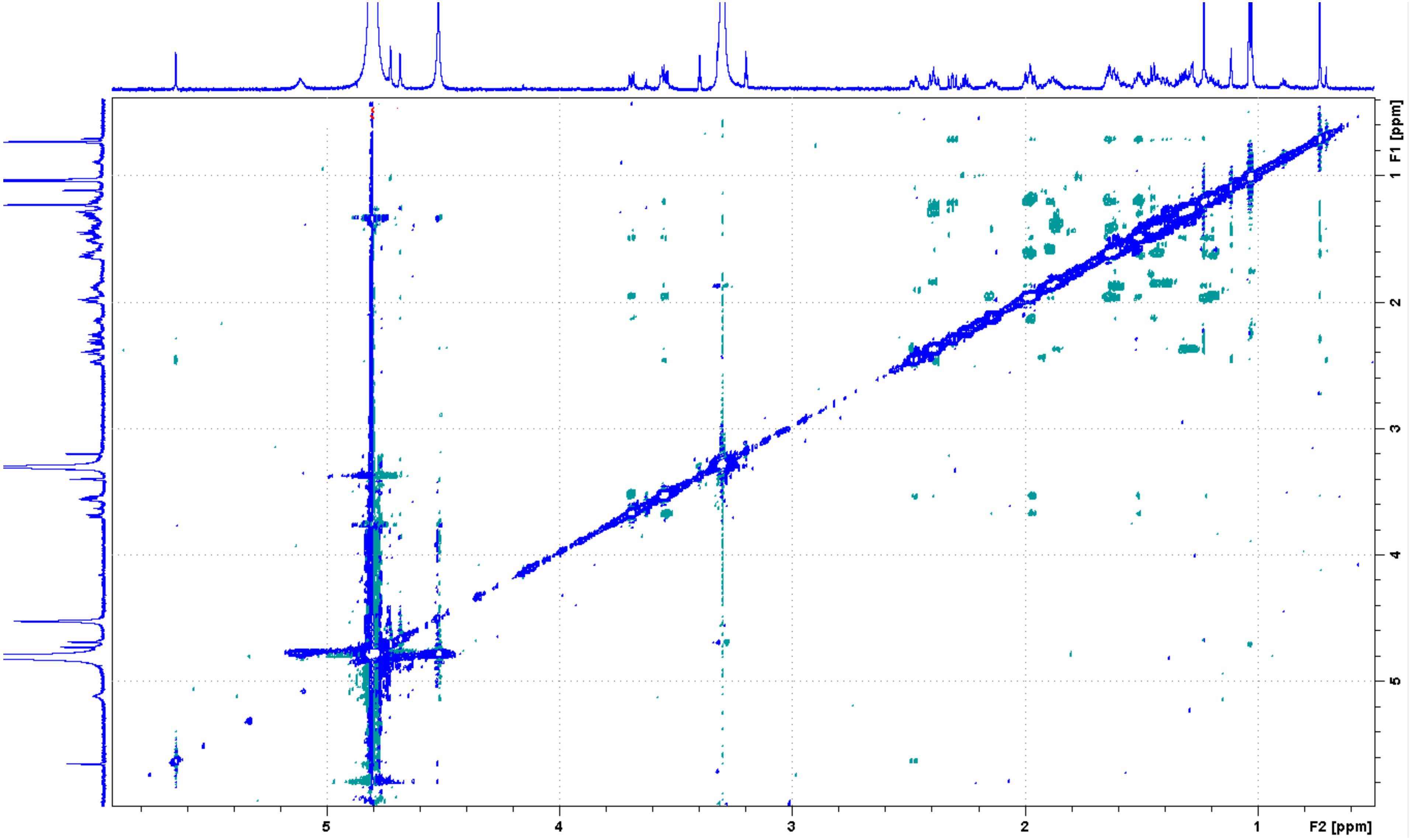

Figure S24. ^{1}H NMR (700.13 MHz, CD_3OD) spectrum of compound **3a**.

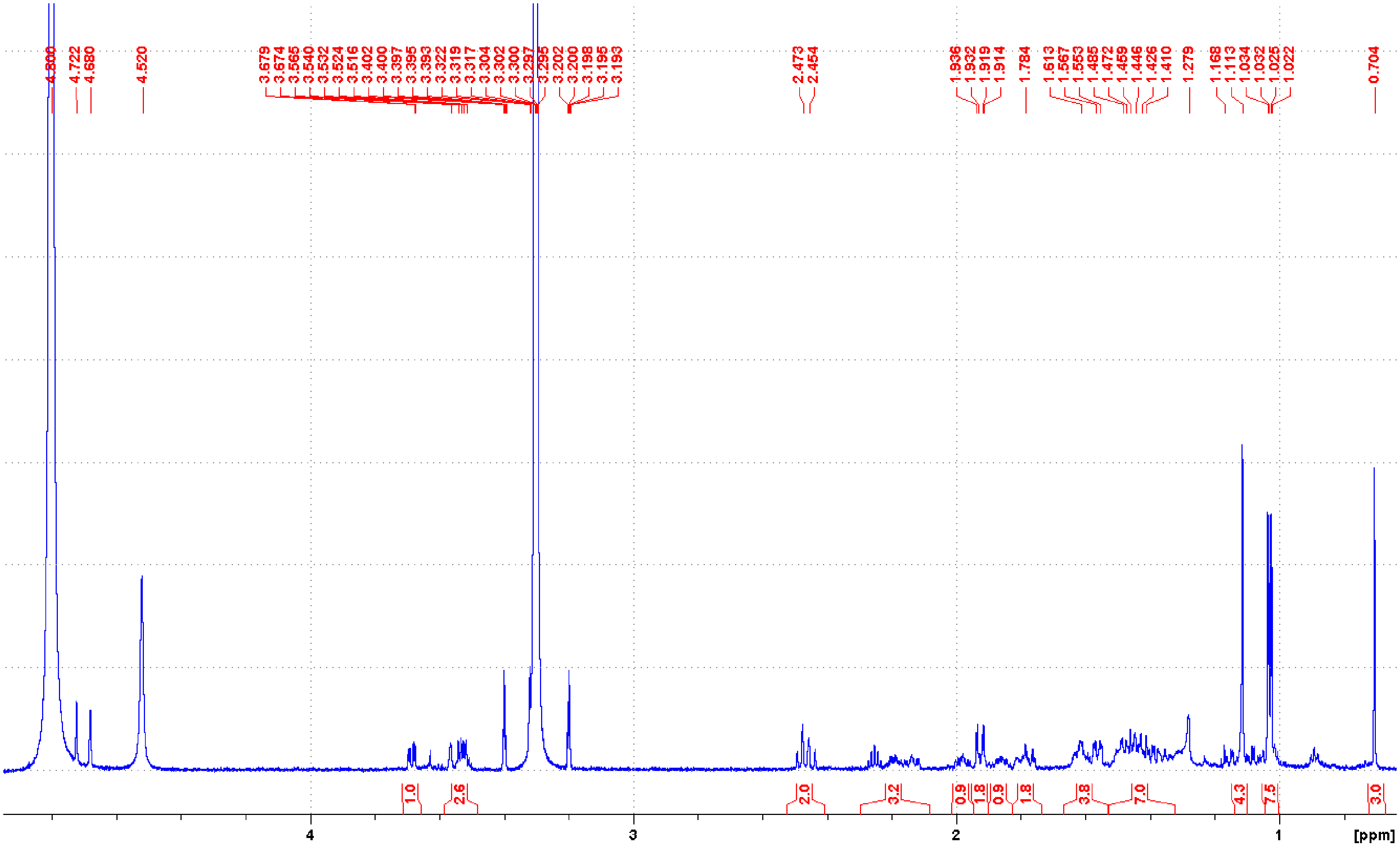

Figure S25. ^{13}C NMR (176.04 MHz, CD_3OD) spectrum of compound **3a**.

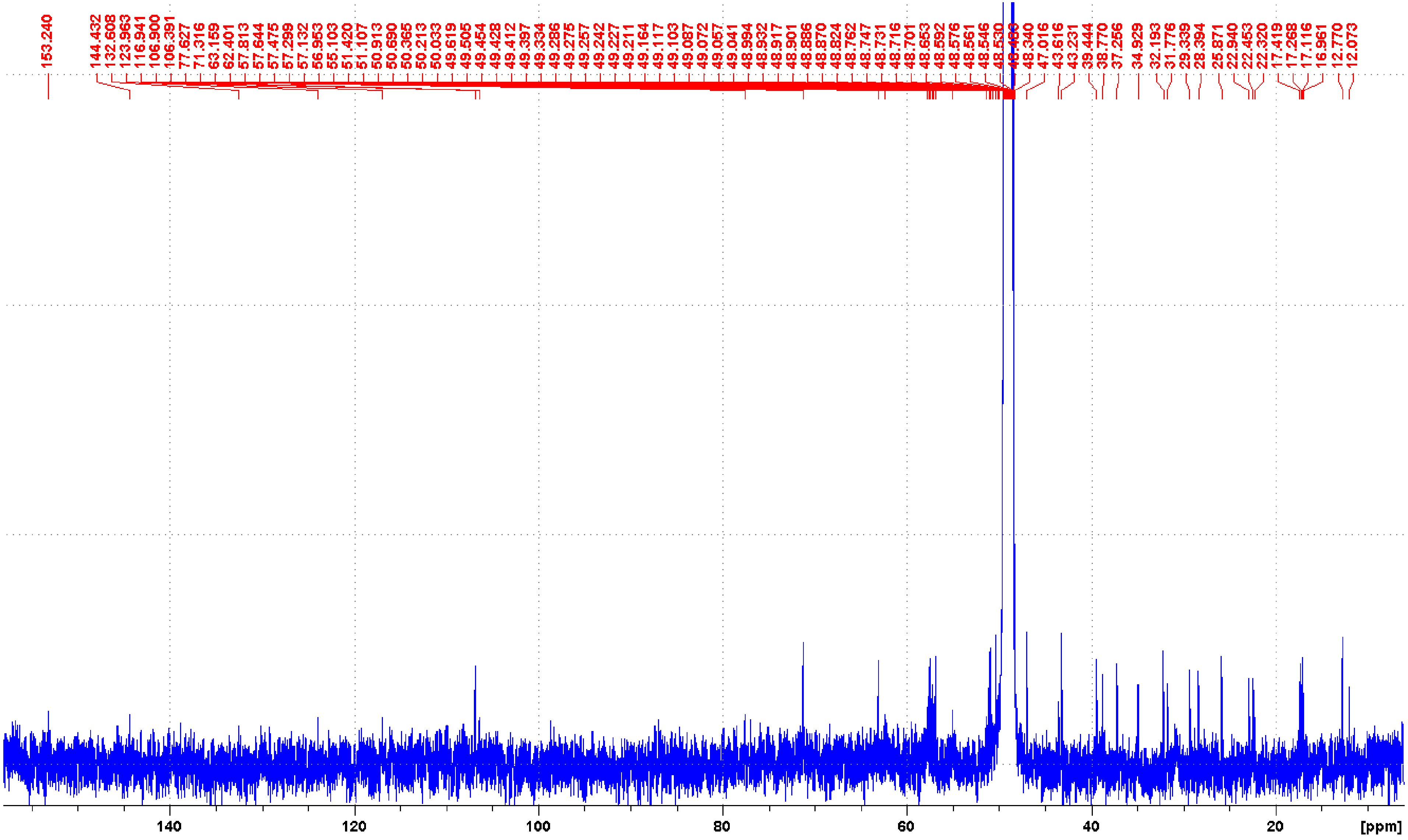

Figure S26. COSY (700.13 MHz, CD_3OD) spectrum of compound **3a**.

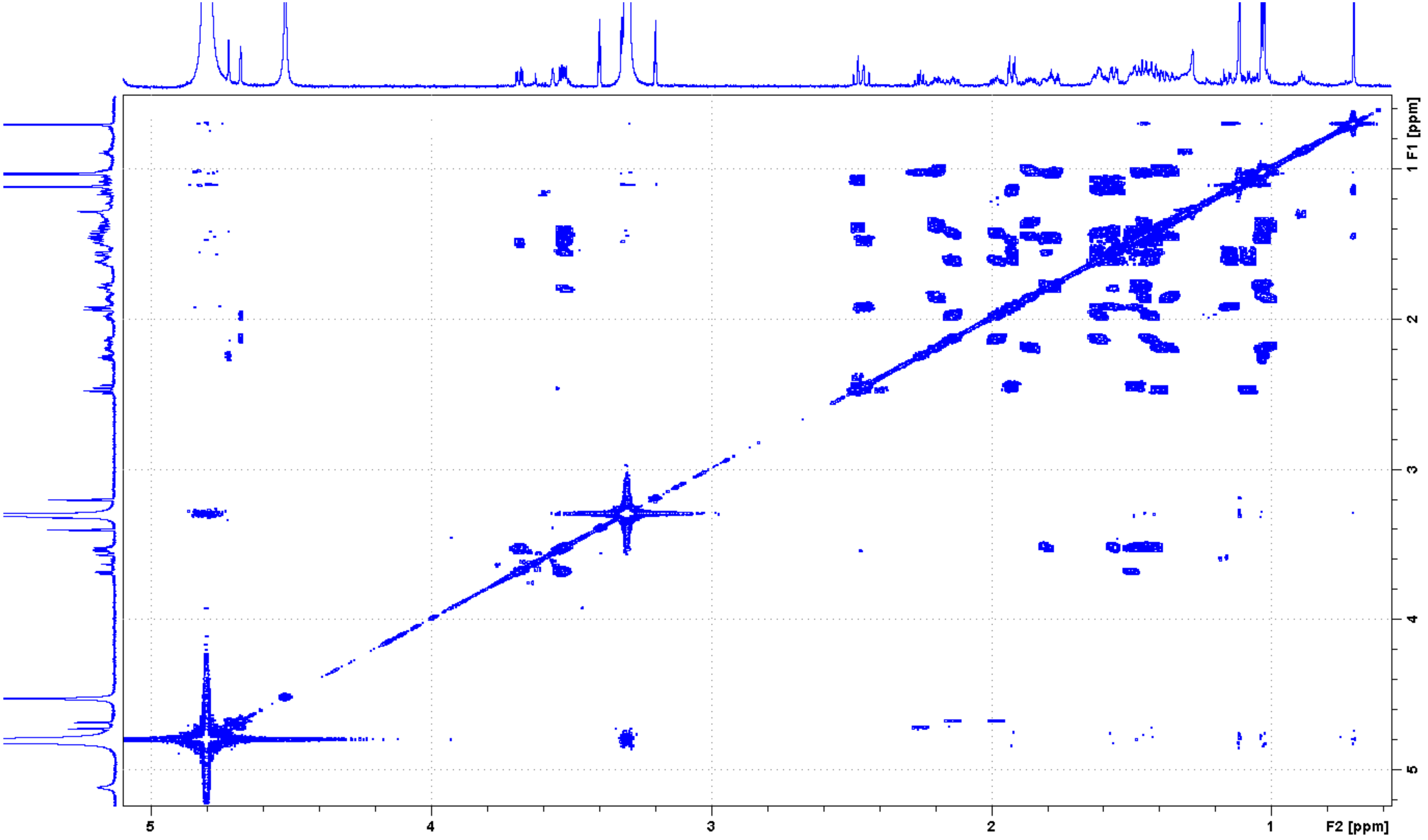

Figure S27. HSQC (700.13 MHz, CD_3OD) spectrum of compound **3a**.

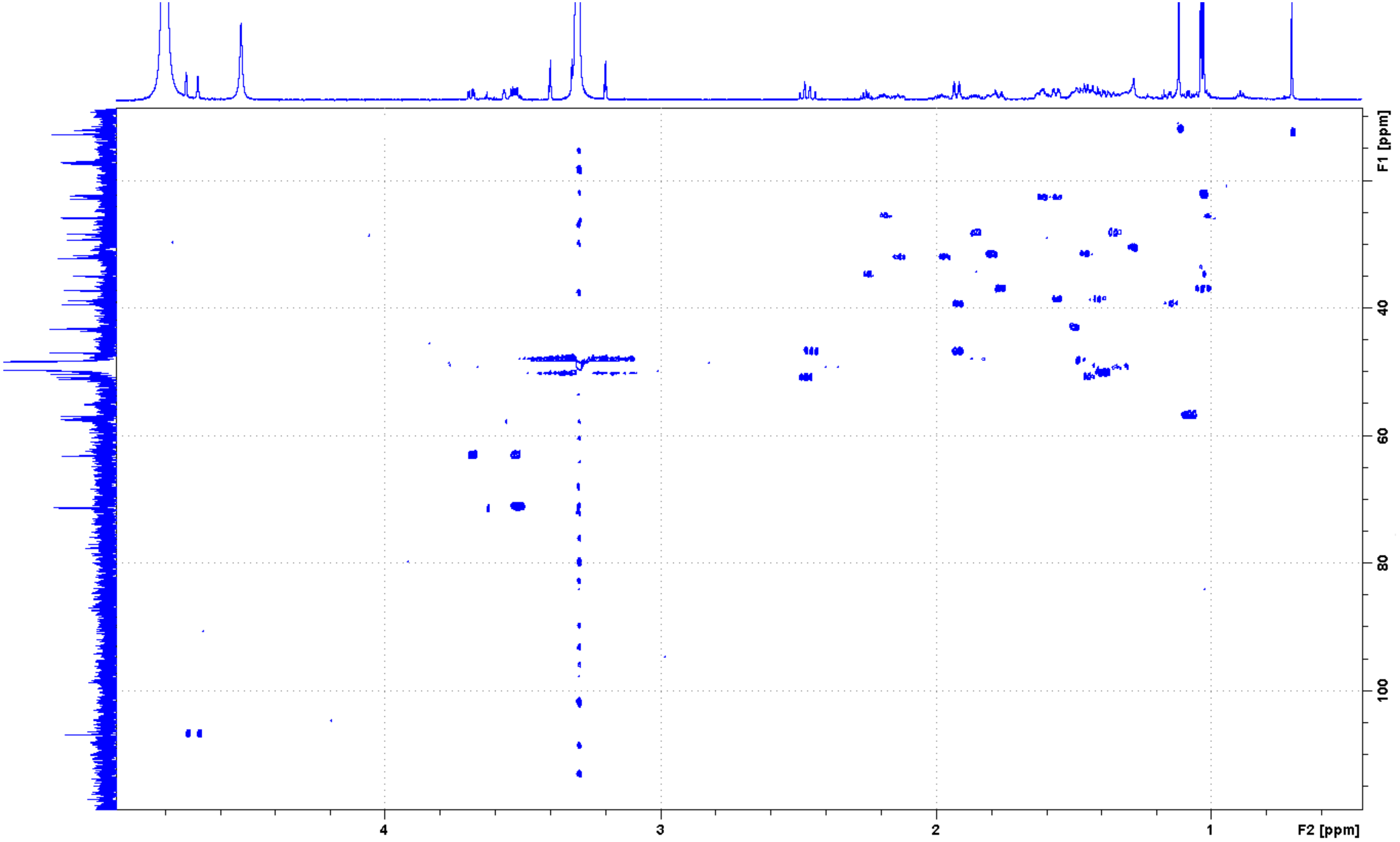

Figure S28. HMBC (700.13 MHz, CD_3OD) spectrum of compound **3a**.

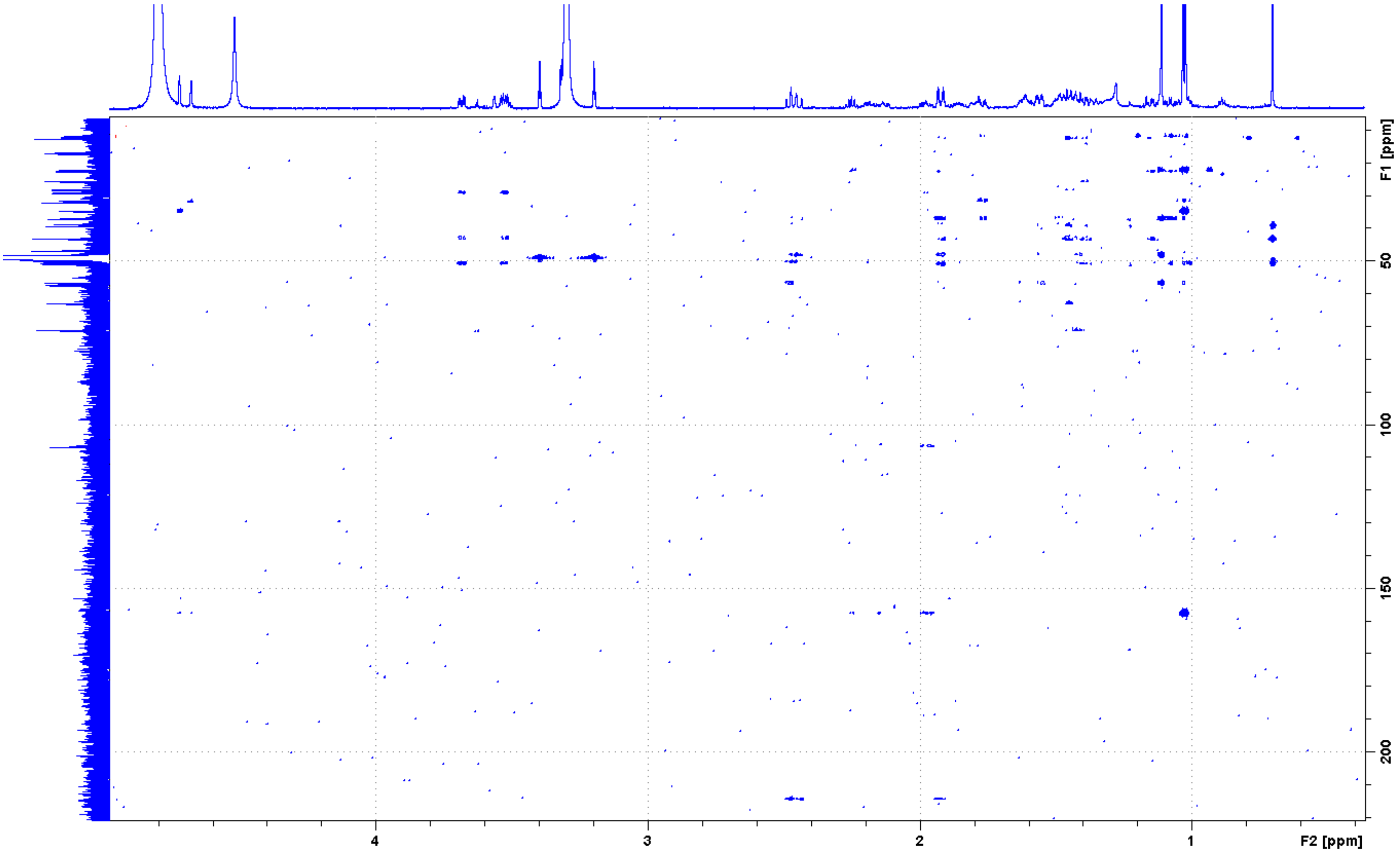

Figure S29. ROESY (700.13 MHz, CD_3OD) spectrum of compound **3a**.

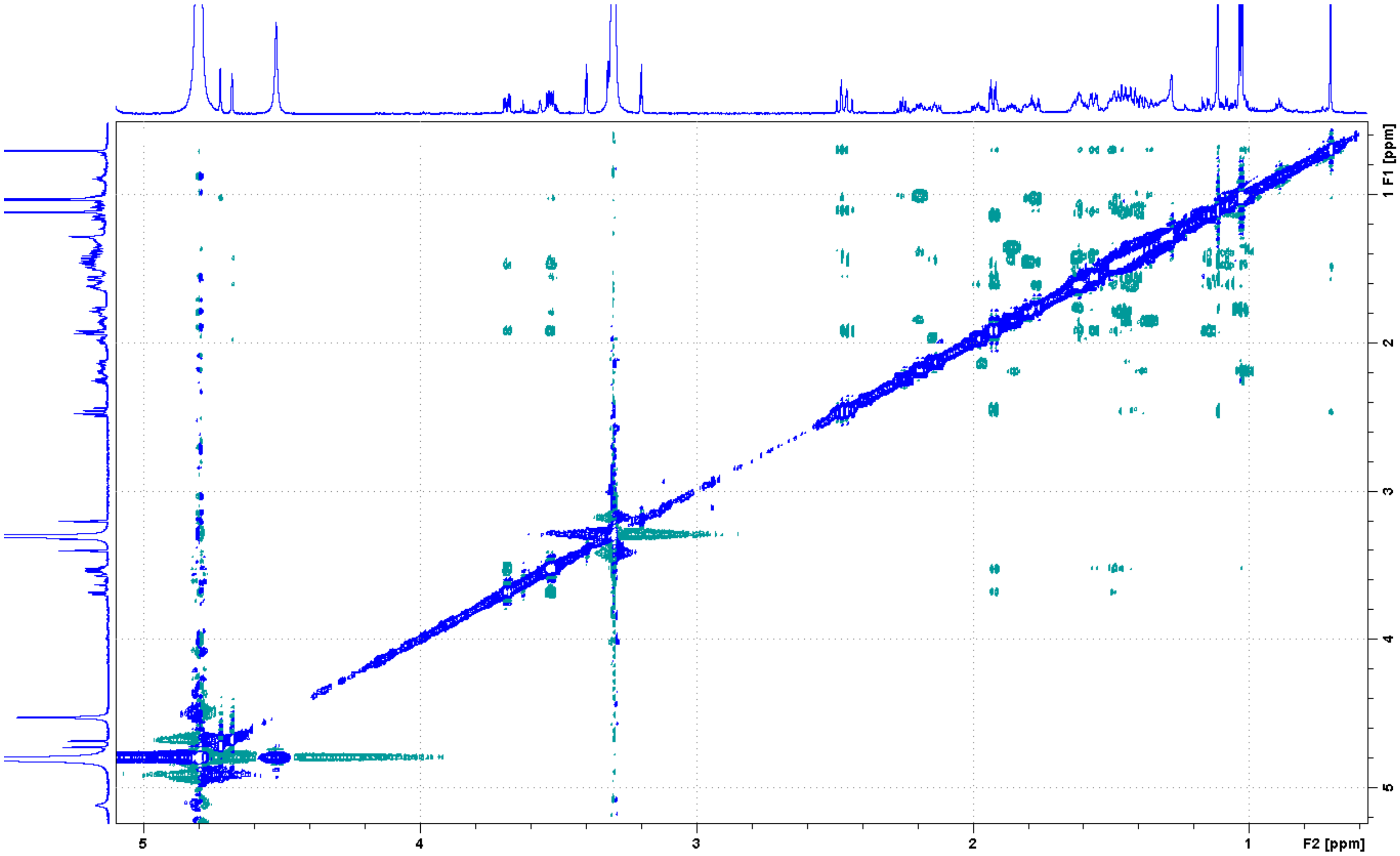

Figure S30. HRESIMS spectra of compound **4**

(–)HRESIMS [M – Na]⁻ ion

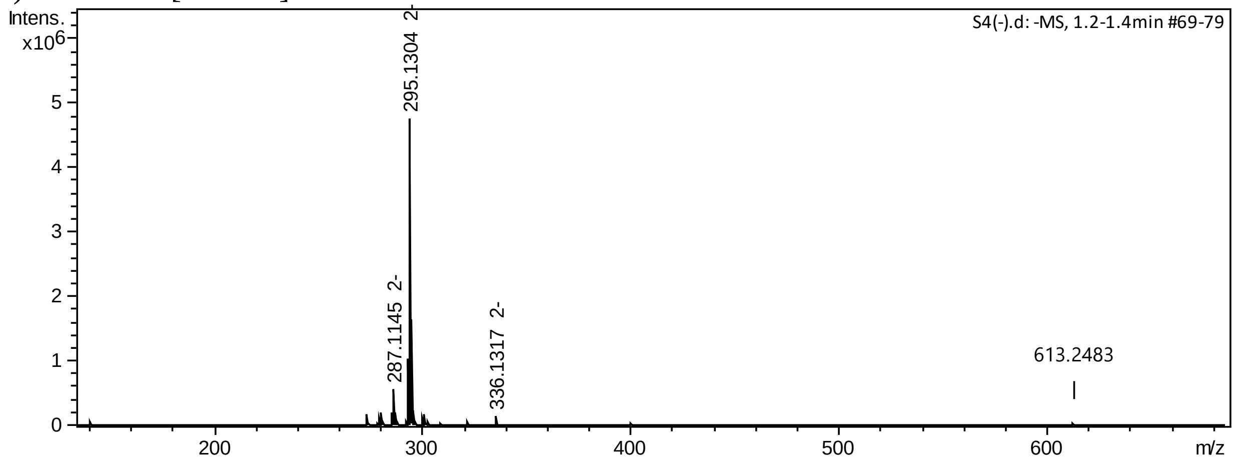

(+)HRESIMS [M + Na]$^+$ ion

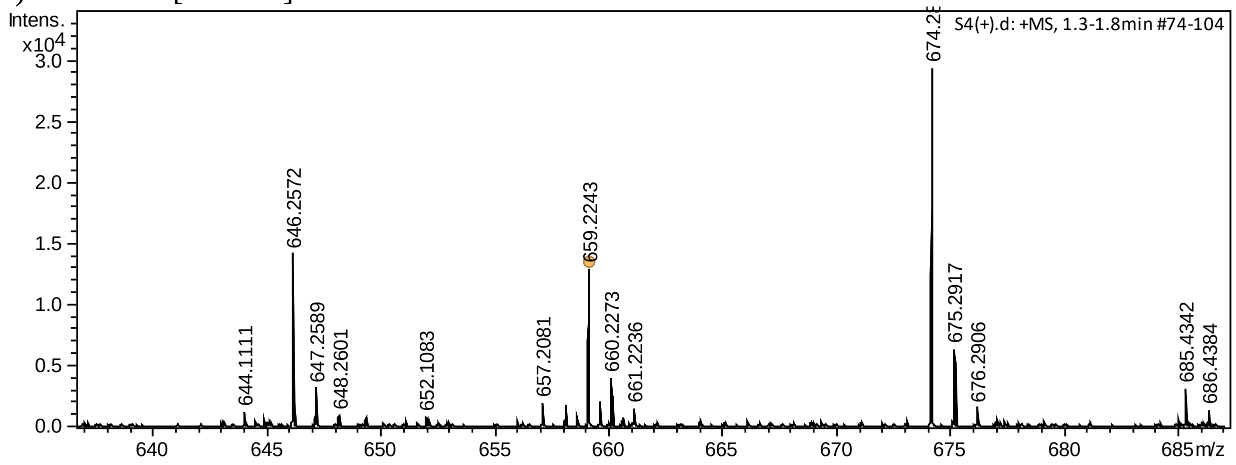

Figure S31. ^{1}H NMR (700.13 MHz, CD_3OD) spectrum of compound **4**

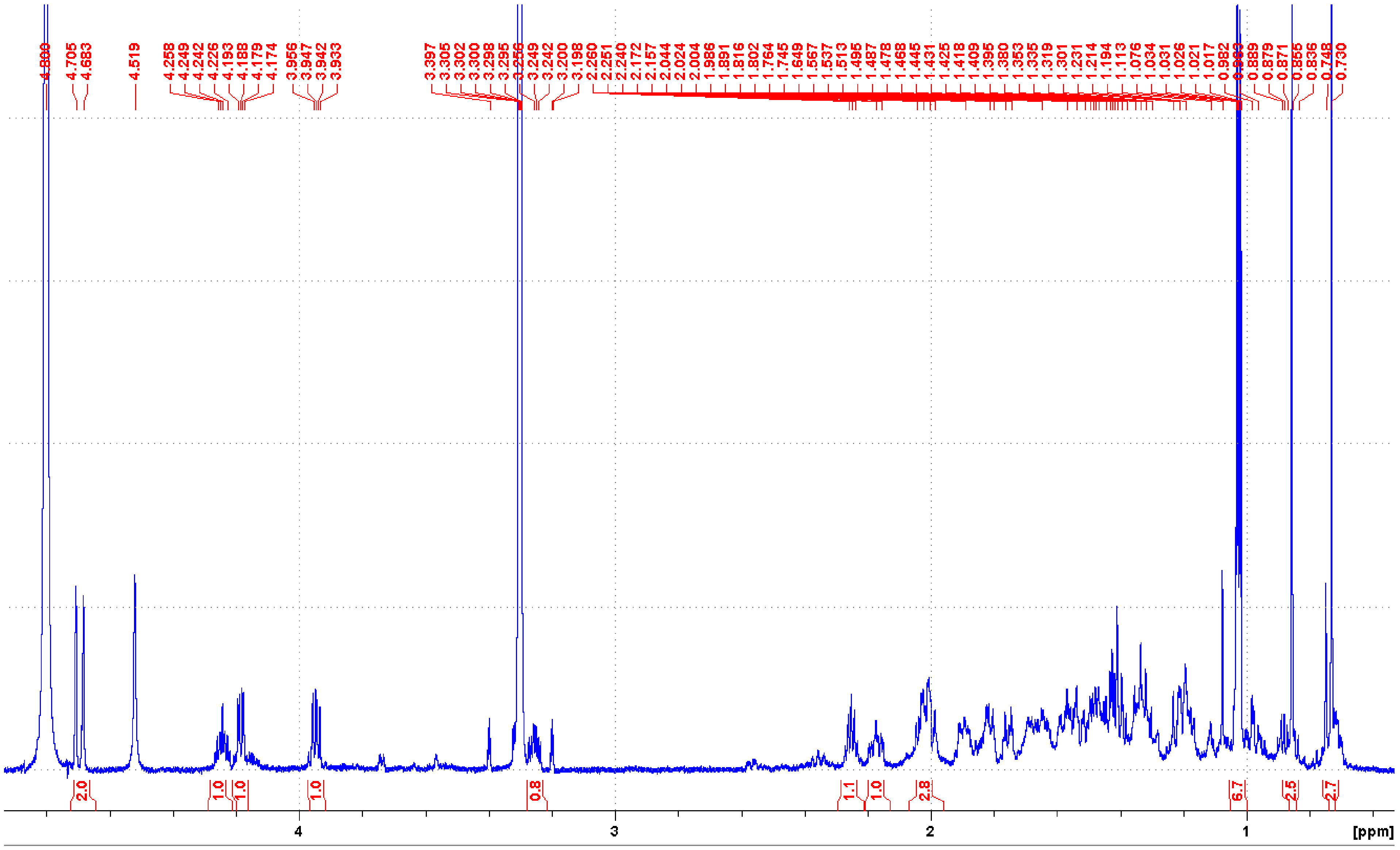

Figure S32. ^{13}C NMR (176.04 MHz, CD_3OD) spectrum of compound **4**

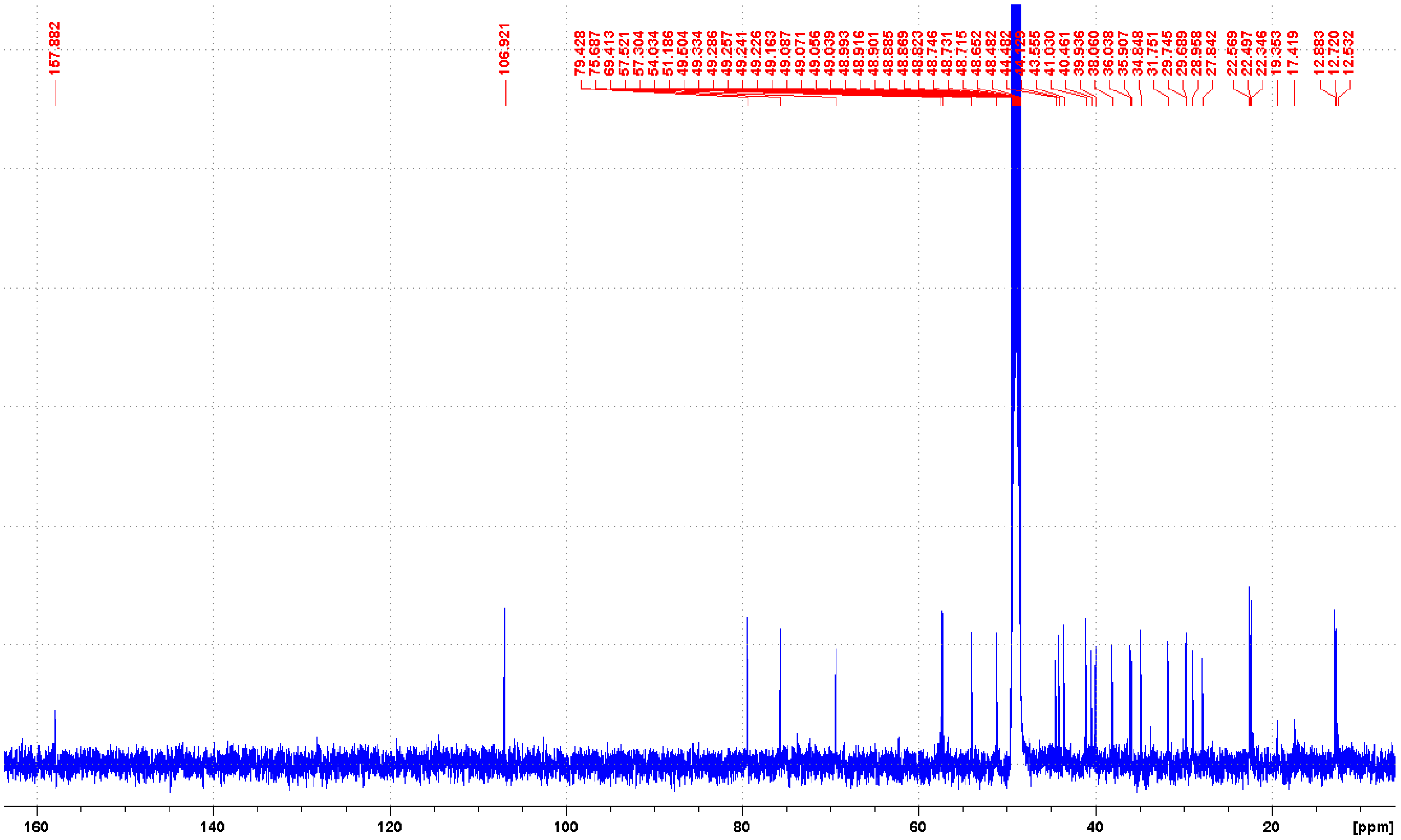

Figure S33. COSY (700.13 MHz, CD_3OD) spectrum of compound **4**

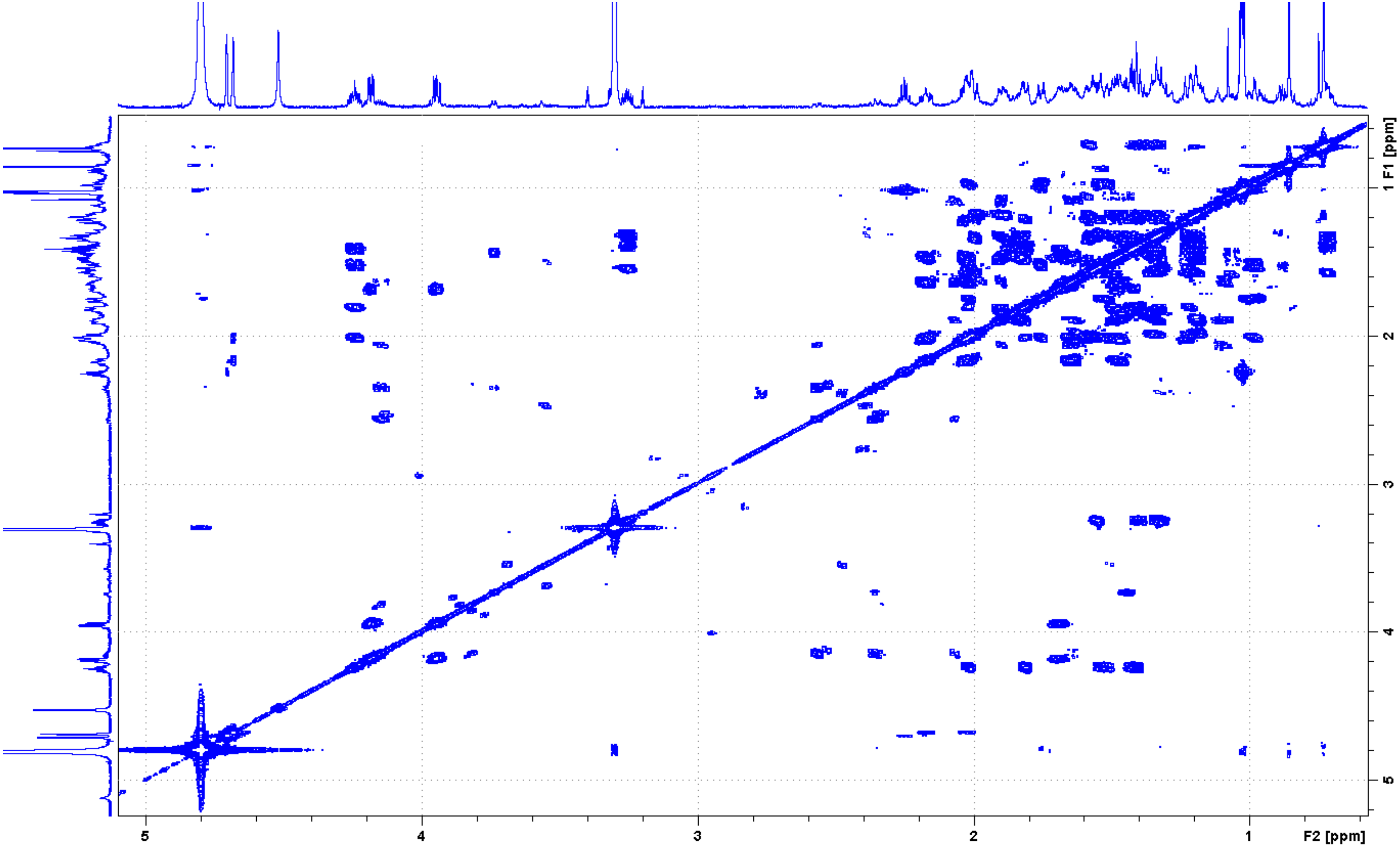

Figure S34. HSQC (700.13 MHz, CD_3OD) spectrum of compound **4**

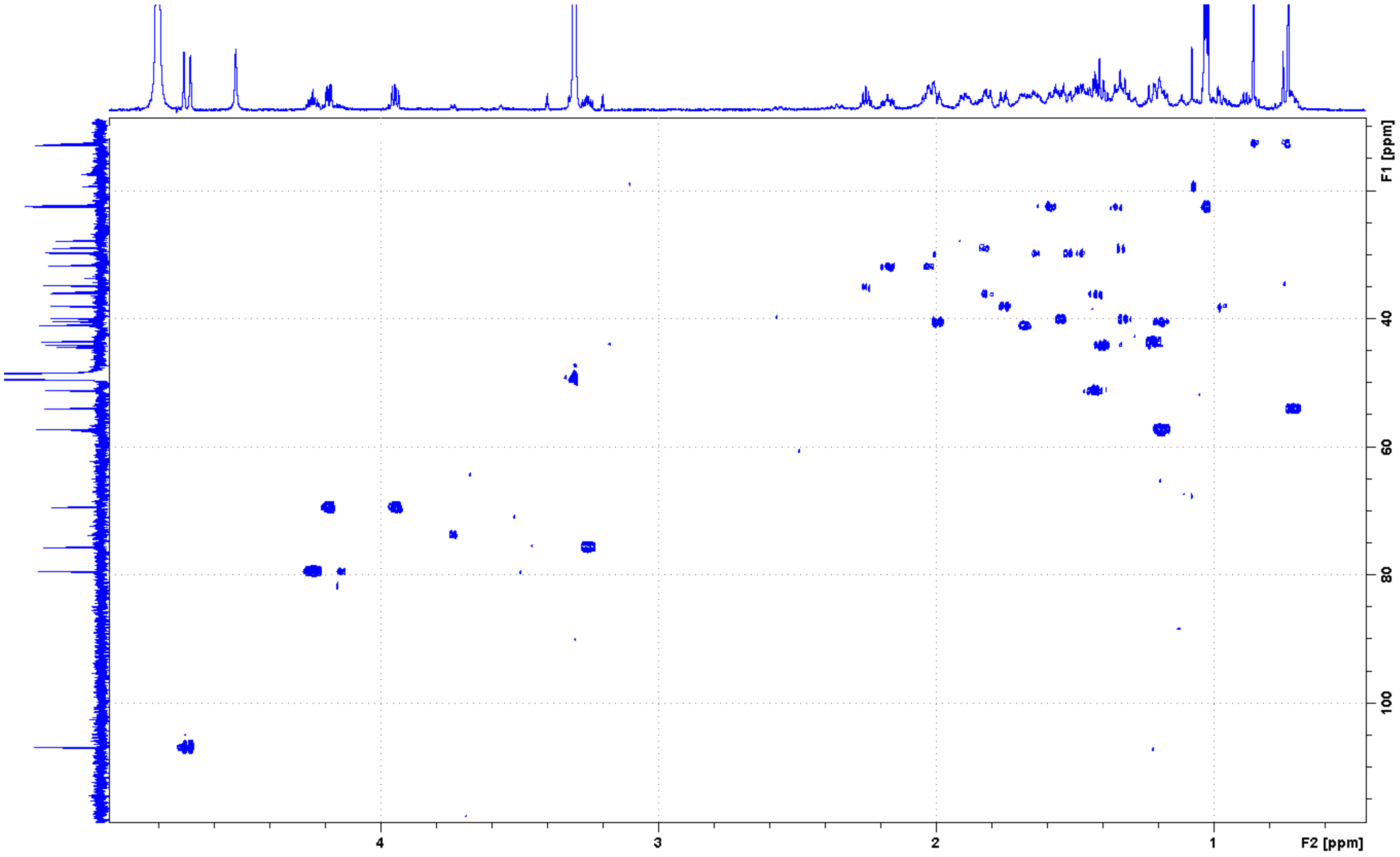

Figure S35. HMBC (700.13 MHz, CD_3OD) spectrum of compound **4**

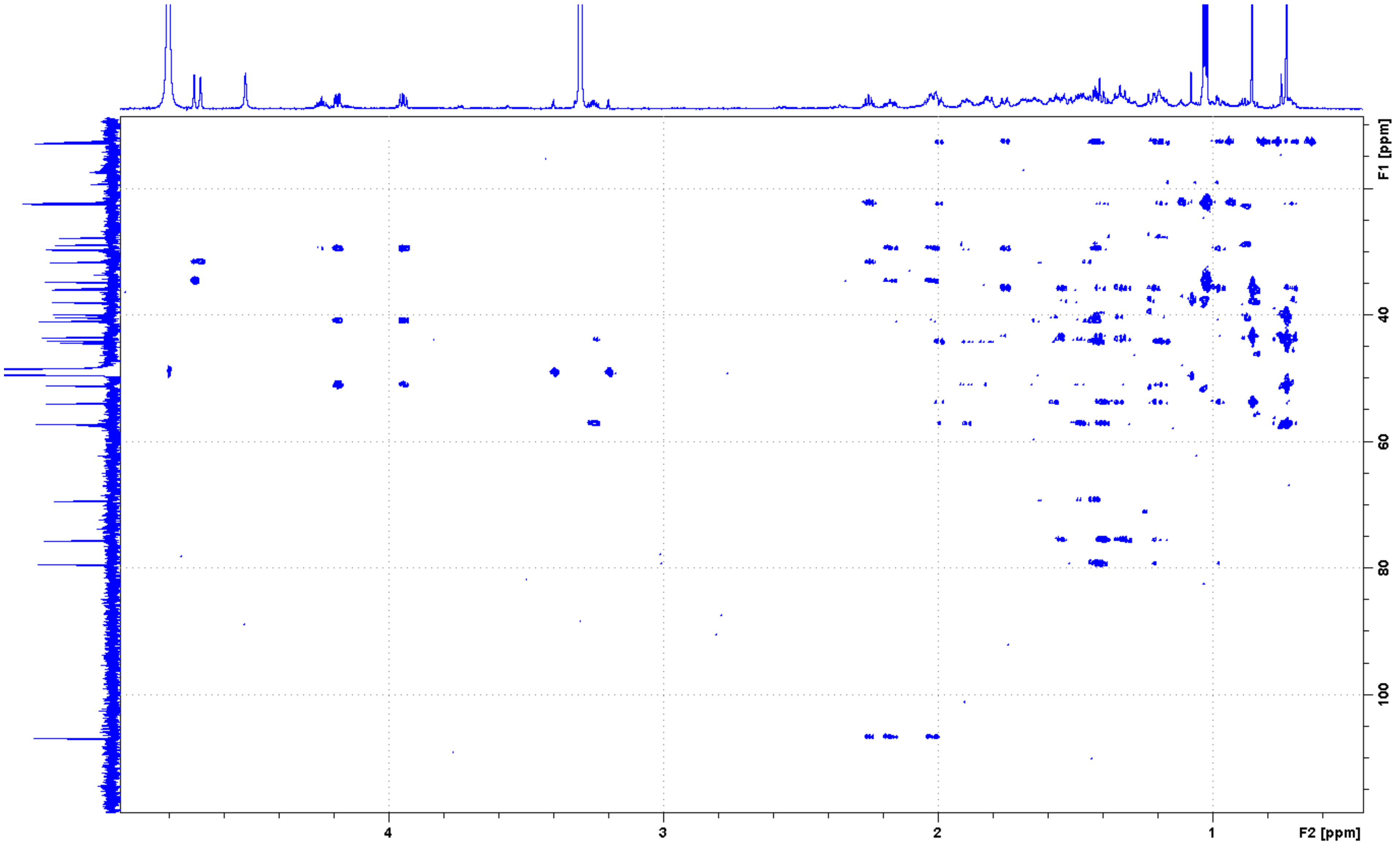

Figure S36. ROESY (700.13 MHz, CD_3OD) spectrum of compound **4**

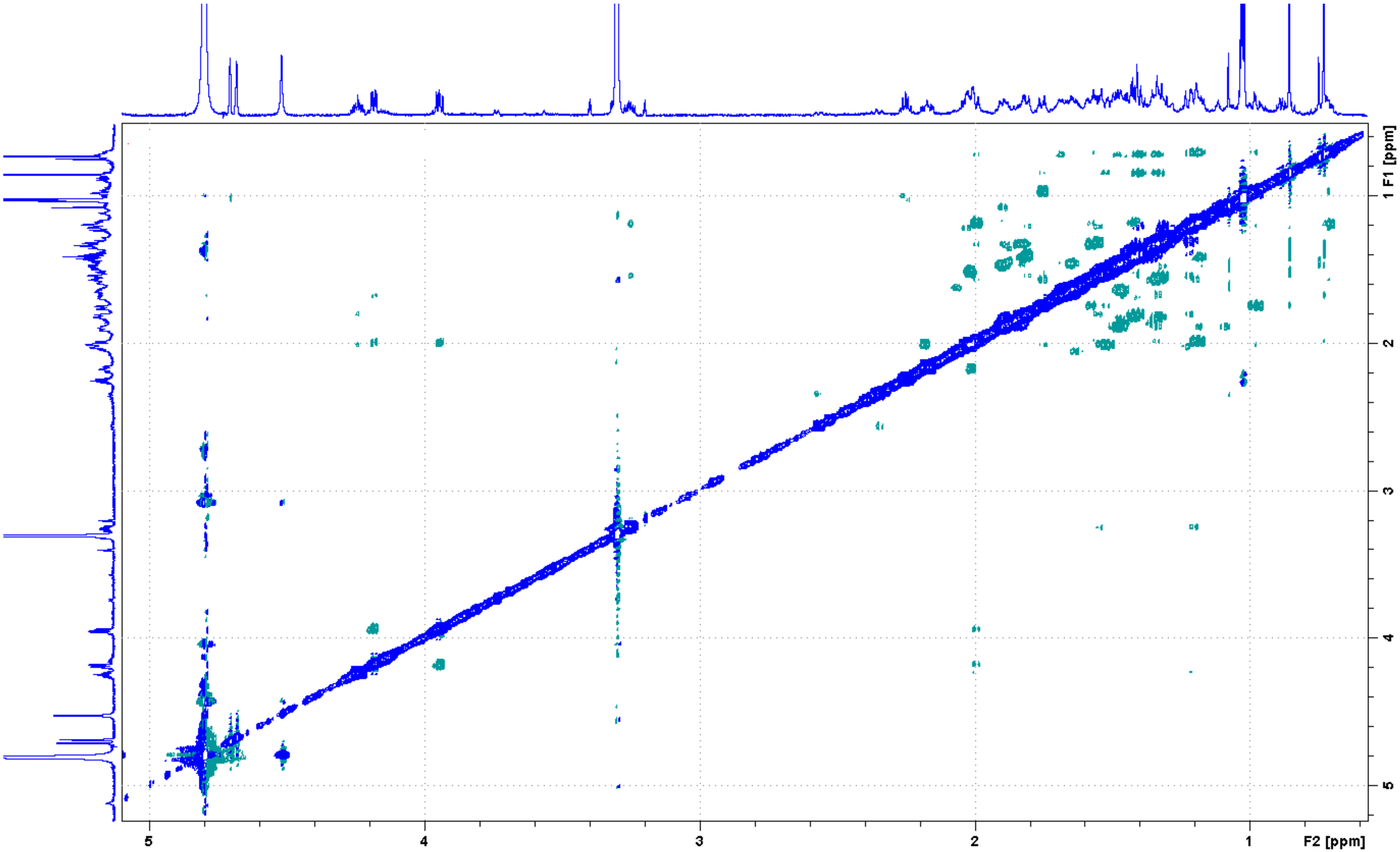

Figure S37. HRESIMS spectra of compound **5**

(–)HRESIMS [M – Na]$^{-}$ ion

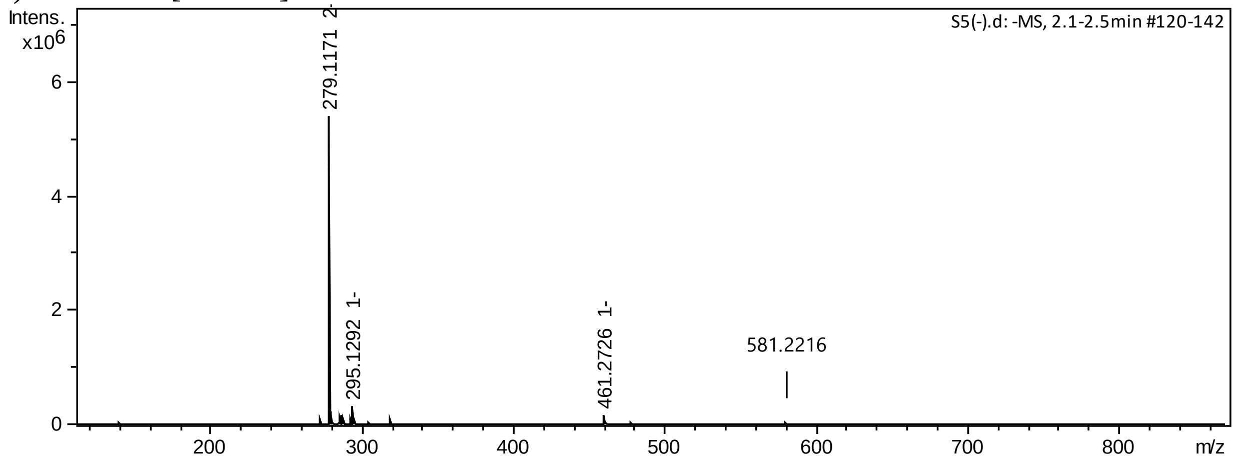

(+)HRESIMS [M + Na]$^{+}$ ion

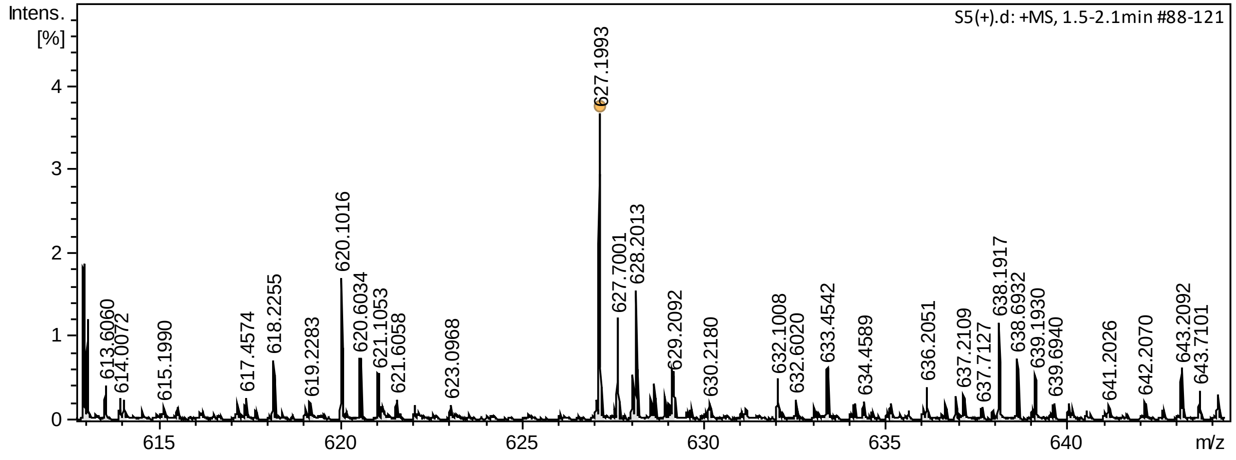

Figure S38. ^{1}H NMR (700.13 MHz, CD_3OD) spectrum of compound **5**

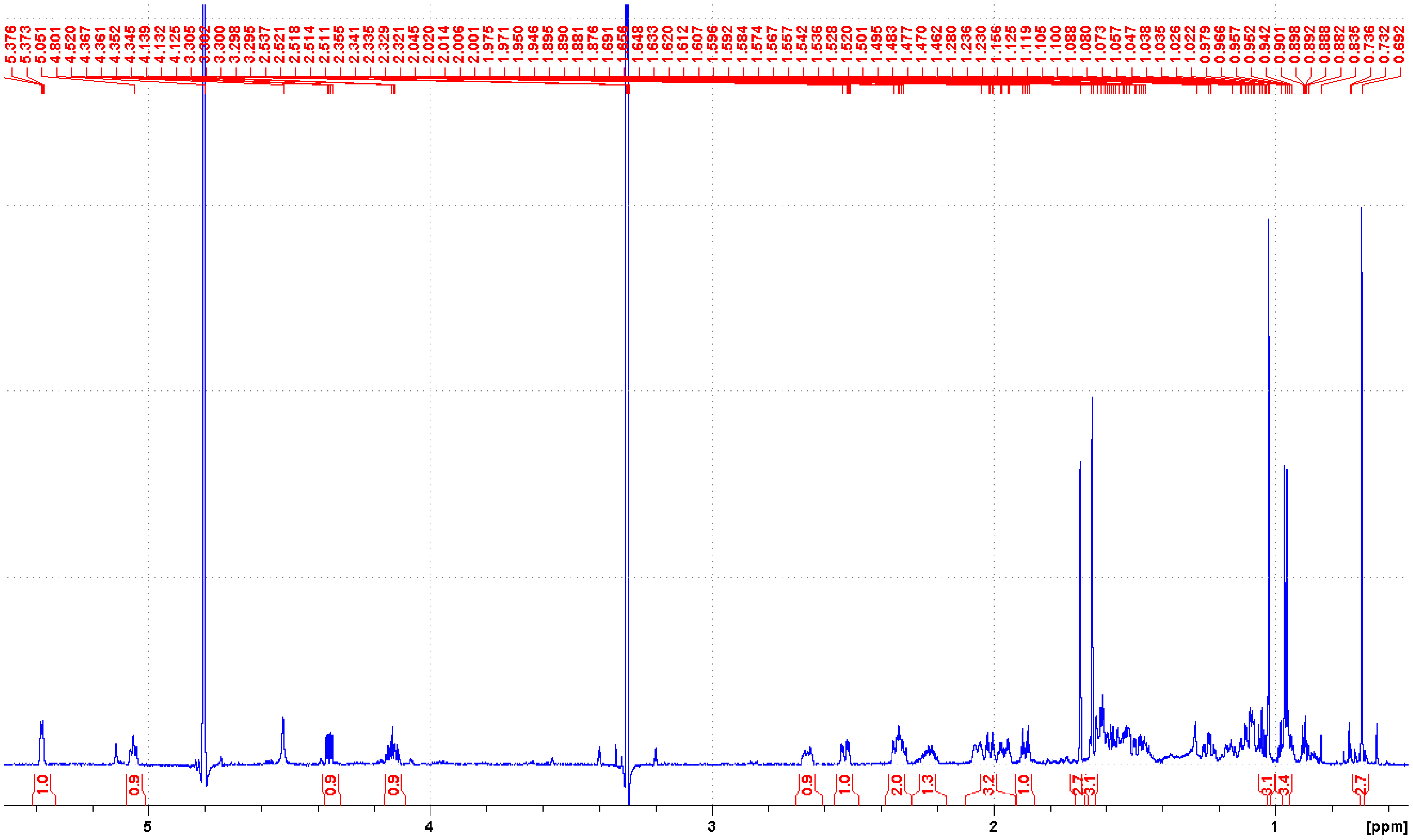

Figure S39. ^{13}C NMR (176.04 MHz, CD_3OD) spectrum of compound **5**

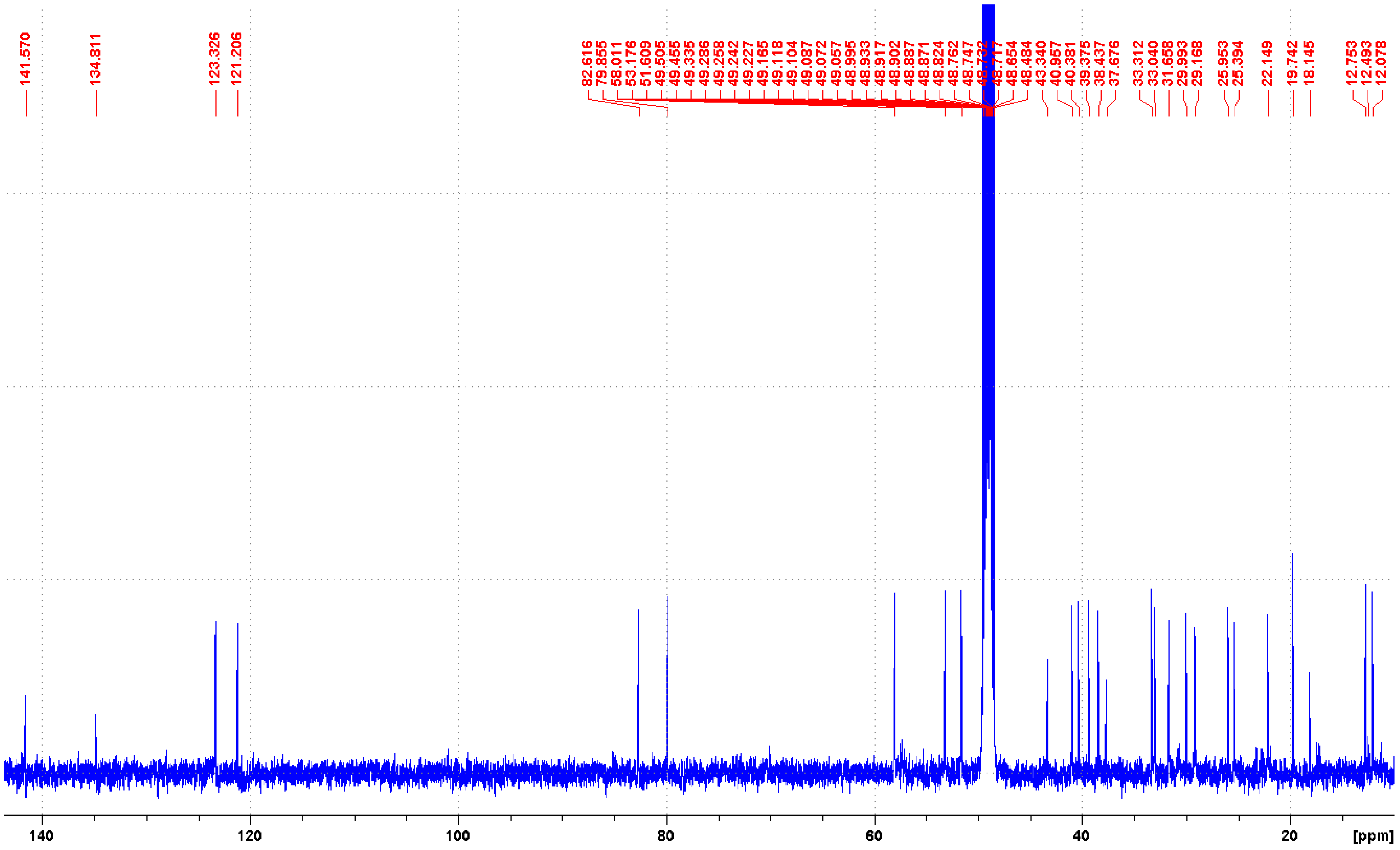

Figure S40. COSY (700.13 MHz, CD_3OD) spectrum of compound **5**

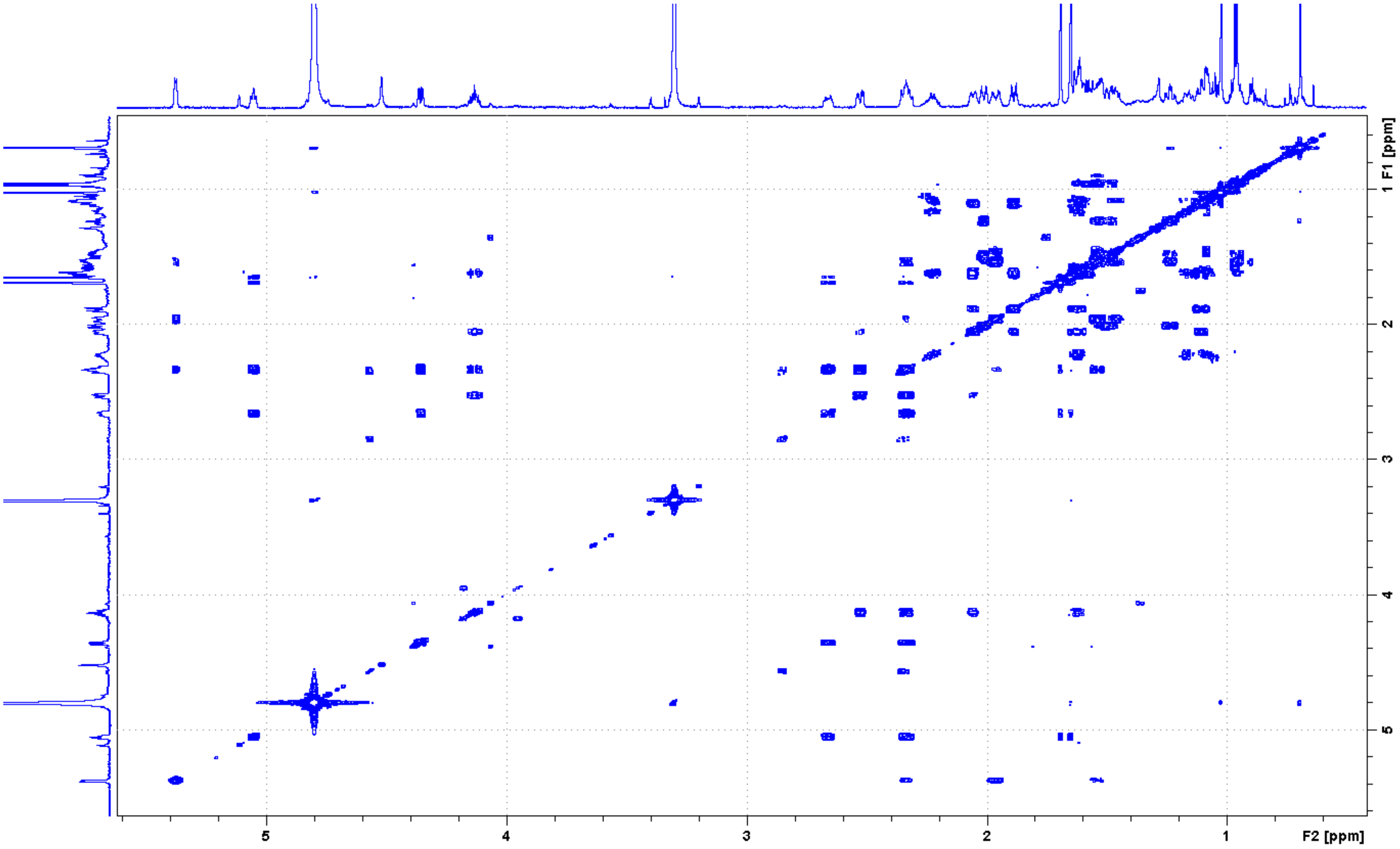

Figure S41. HSQC (700.13 MHz, CD_3OD) spectrum of compound **5**

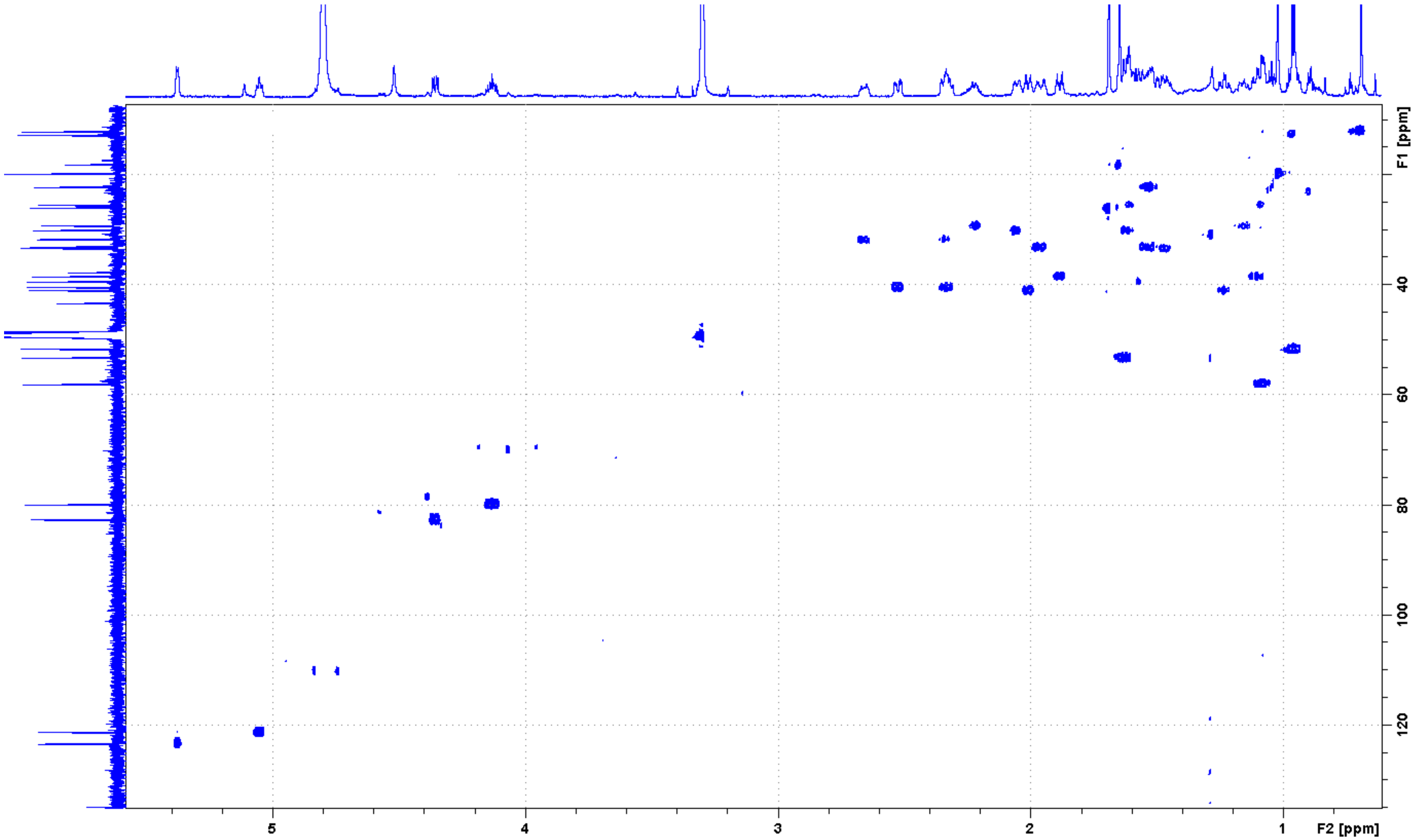

Figure S42. HMBC (700.13 MHz, CD_3OD) spectrum of compound **5**

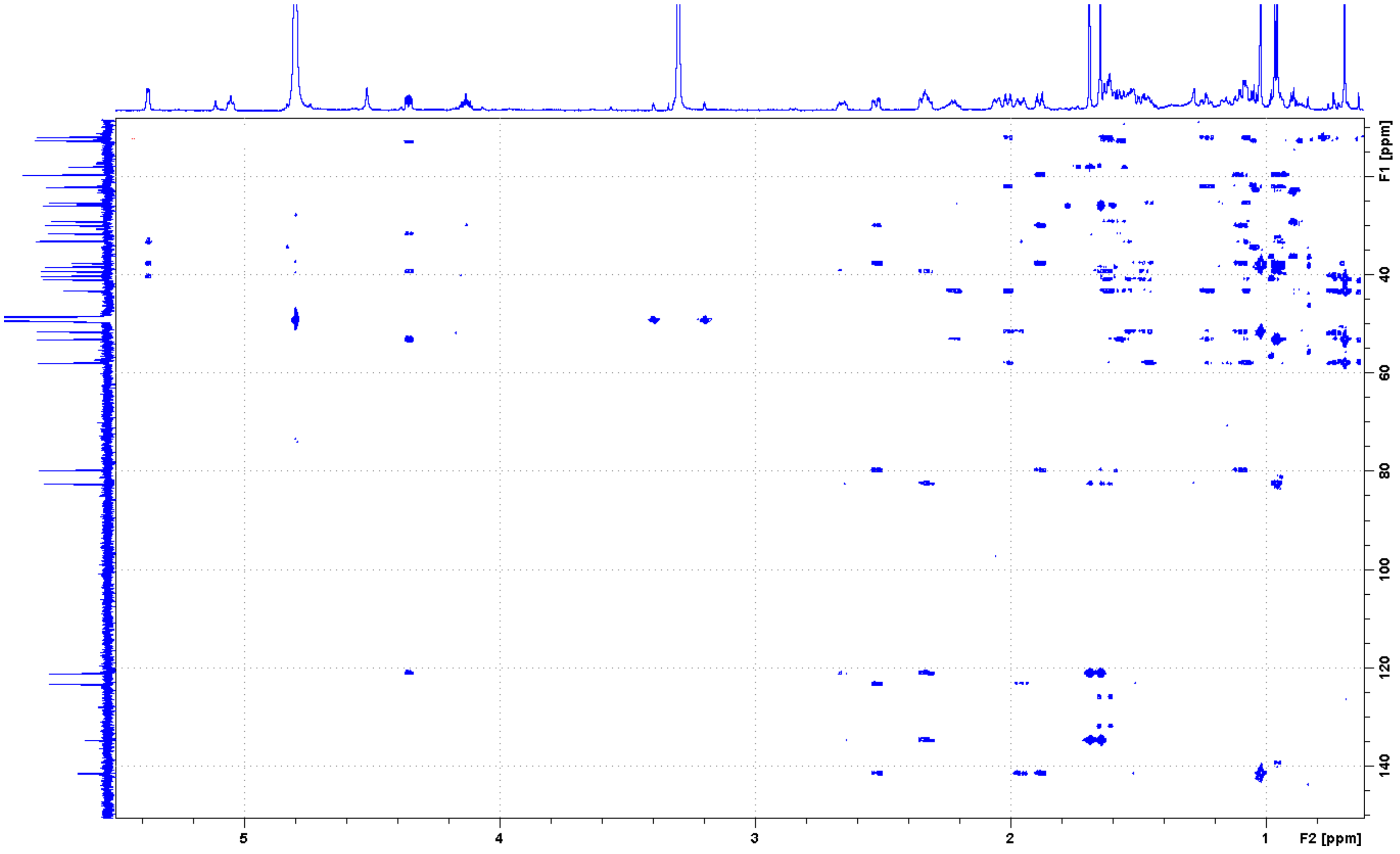

Figure S43. ROESY (700.13 MHz, CD_3OD) spectrum of compound **5**

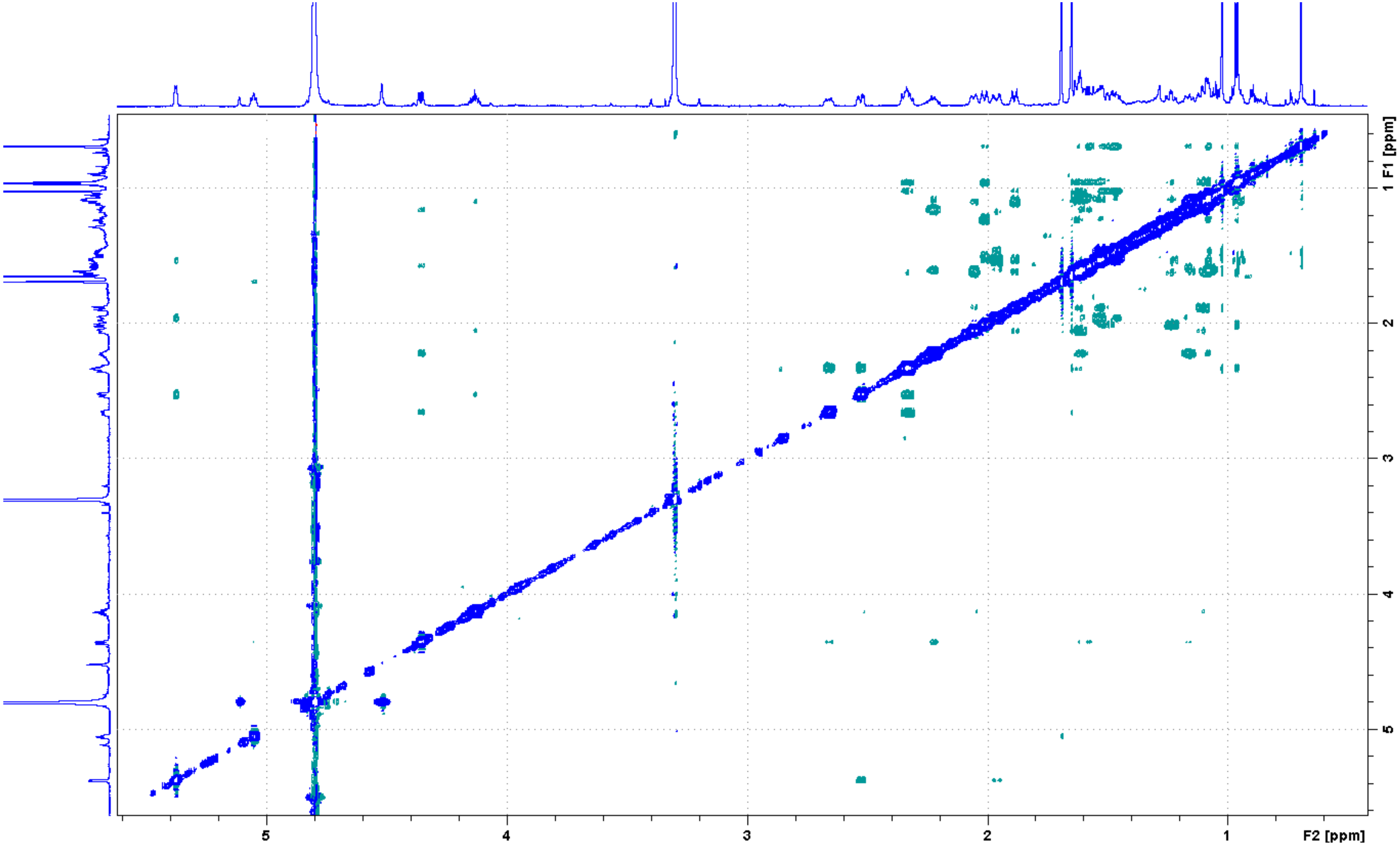

Supplement: Supplementary file 1 [file marinedrugs-20-00164-s001.zip › marinedrugs-1599373-supplementary.pdf]
